# Reversible Redox Chemistry of Anionic Imidazole-2-thione-Fused 1,4-Dihydro-1,4-diphosphinines

**DOI:** 10.1021/acs.inorgchem.1c03620

**Published:** 2022-03-08

**Authors:** Mridhul R. K. Ramachandran, Gregor Schnakenburg, Moumita Majumdar, Zsolt Kelemen, Dalma Gál, Laszlo Nyulászi, René T. Boeré, Rainer K. Streubel

**Affiliations:** † Institut für Anorganische Chemie, Rheinische Friedrich-Wilhelms-Universität Bonn, Gerhard-Domagk-Straße 1, D-53121 Bonn, Germany; ‡ Department of Chemistry, Indian Institute of Science Education and Research, Pune 411008, Maharashtra, India; § Department of Inorganic and Analytical Chemistry and MTA-BME Computation Driven Chemistry Research Group, Budapest University of Technology and Economics, Szt Gellert ter 4, 1111 Budapest, Hungary; ∥ Department of Chemistry and Biochemistry, University of Lethbridge, 4401 University Drive West, Lethbridge, AB T1K3M4, Canada

## Abstract

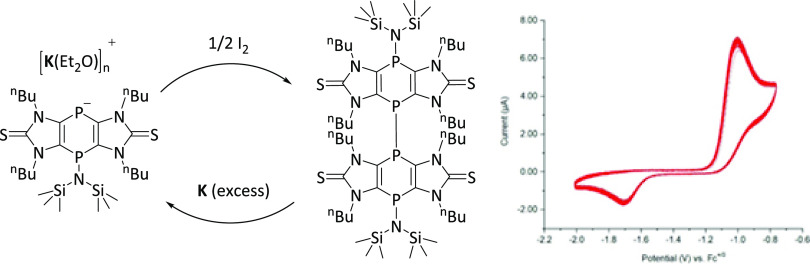

Anionic 1,4-dihydro-1,4-diphosphinines were synthesized from tricyclic 1,4-diphosphinines and isolated as blue powdery salts M[**2a**–**2c**]. Reaction of solutions of these monoanions with iodomethane led to *P*-methylated compounds **3a**–**3c**. An oxidation/reduction cycle was examined, starting from solutions of K[**2a**] via P–P coupled product **4a** and back to K[**2a**], and the recyclability and redox chemistry of this cycle were confirmed by experimental and simulated cyclic voltammetry analysis, which is proposed as a potential 2-electron cathode for rechargeable cells. TD-DFT studies were used to examine species that might be involved in the process.

## Abstract 2

Synthesis of a set of stable P-anionic 1,4-dihydro-1,4-diphosphinines is described, including an oxidation/reduction cycle of one example, confirmed by experimental and simulated cyclic voltammetry analysis as well as detailed DFT studies. The results indicate that main group phosphorus compounds have unexplored potential for the development of new cathode materials.

## Introduction

**Figure 1:**
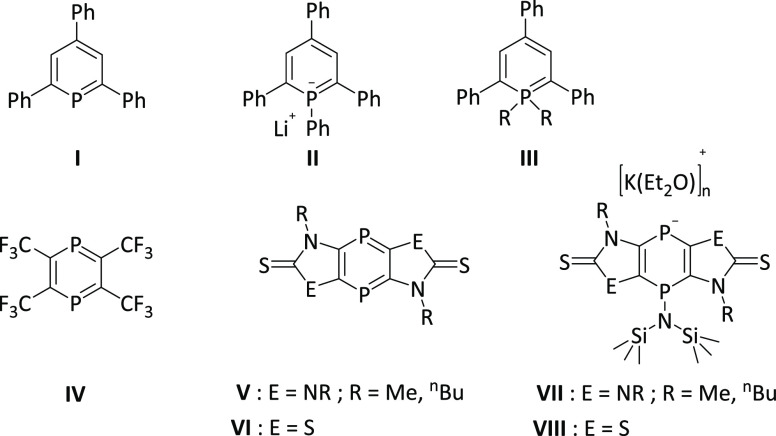
First P^III^-phosphinine **I**, anionic derivative **II**, P^V^-phosphinines **III**, first 1,4-diphosphinines **IV**–**VI**, and anionic 1,4-diphosphinines **VII** and **VIII**.

Since the landmark discovery of 2,4,6-triphenylphosphinine **I** by Märkl in 1966,^1^ its reactivity has been studied extensively.^2−5^ The electrophilic nature of the P center has been exploited to gain access to a variety of compounds, some of which via transformation of anionic derivatives **II** into **III** possess a four-coordinate P(V) center (Figure 1).^6^ A recent report by Müller et al. has also dealt with nucleophilic substitution using Grignard or organolithium reagents, resulting in λ^4^σ^3^-phosphinine anions.^7^ The reason for the selective reaction of (anionic) nucleophiles at the P atom is the large orbital coefficient of the low-lying LUMO.^8^ So far, the redox chemistry of λ^5^-phosphinines **III** has been investigated for optovoltaic applications in the design of organic light-emitting diodes (OLEDs).^9^ Similarly, P^V^-phospharhodamines have been extensively investigated for their photovoltaic applications.^10^ All these six-membered phosphorus heterocycles, mostly dominated by the P^V^-phospholes, share anodic photovoltaic behavior largely centered in the unsaturated hydrocarbon portion of the molecules with the P(V) centers uninvolved in redox processes.^11−15^

The chemistry of 1,4-diphosphinines started in 1976 with the first derivative **IV**, synthesized by Kobayashi et al.,^16^ but in contrast to **I**, it could not be isolated.^17−19^

The advent of stable tricyclic 1,4-diphosphinines fused to heterocyclic-2-thiones, such as **V**(20) and **VI**,^21^ has enabled systematic investigation of their chemistry.^22−24^ As one example, the reactivity of 1,4-diphosphinines **V** in [4 + 1] and [4 + 2] cycloaddition reactions has been reported, including unusual reactions with dichalcogenides.^23,24^ Additionally, the electrophilic P centers of **V** were explored by the addition of anionic nucleophiles, forming P-anionic species, which were not isolated but could be quenched with iodomethane to yield neutral *P*-Me substitution products.^22^ Using a thiazol-2-thione-based 1,4-diphosphinine, first access to an isolable anionic tricyclic 1,4-dihydro-1,4-diphosphinine derivative (**VIII**) was achieved, which could be oxidized by I_2_ to give a product with a P–P bond.^21^ Cyclic voltammetry studies on neutral **V** and **VI** disclosed a rich cathodic electrochemistry involving the P^III^ center, in contrast to λ^5^-phosphinines (*vide supra*).^20,21^

Beyond these rather intensively investigated five- and six-membered P-heterocycles, the redox chemistry of unsaturated three-membered rings, *i.e.*, 4,5,6-triphospha[3]radialene, was studied too, which could be reduced to structurally confirmed dianions. Furthermore, cyclic voltammetry studies showed a reversible initial one-electron reduction and, on further scanning, revealed a second reversible redox couple, but no experiments aimed at demonstrating suitability as electrode materials were performed such as multicycle voltammograms, *i.e*., the robustness of the cycles was not proven.^25−27^

Apart from cyclic P-systems, (acyclic) diphosphenes can be easily reduced to form anionic radical species, but again, no further studies were performed.^28^ Electrochemical investigations of P–P (single) bond formation have been reported from both anodic and cathodic processes involving dimerization of phosphaalkene radical anions, cationic 1-phosphabutadiene radicals, or even R_3_P^•+^ centers, going back as far as Märkl et al.^29−31^ Other main group element redox systems that store energy in element–element bonds such as S–S bonds are the lithium–sulfur and sodium–sulfur storage batteries.^32−35^ Herein, we report chemical and voltammetric investigations of the anionic 1,4-diphosphinine derivatives **VII** including their conversion into P–P coupled products and, subsequently, the successful chemical reduction to reform type **VII** salts, thus closing the redox cycle (also) in solution.

## Results and Discussion

**Scheme 1:**
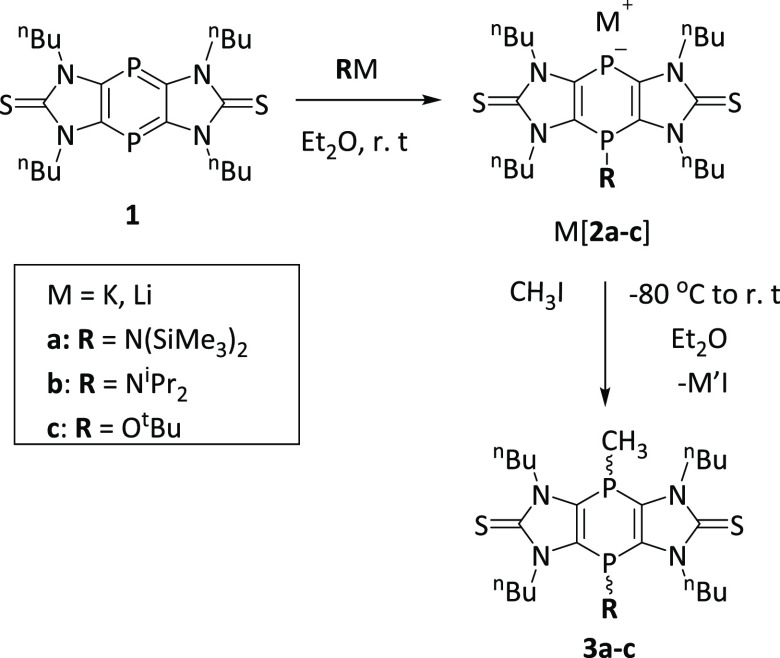
Synthesis and Reaction of M[**2a**–**2c**] to give *P*-Methylated Products **3a**–**3c**

When 1,4-diphosphinine **1**(20) was treated with KHMDS in diethyl ether, a drastic color change from red to deep blue occurred. Evaporation of the solvent *in vacuo* yielded K[**2a**] as a blue-violet powder (Scheme 1). Application of the same protocol, but using LDA and KO*t*Bu, afforded Li[**2b**] and K[**2c**] selectively, which, again, were obtained as blue-violet powders. The ^31^P{^1^H} NMR data of M[**2a**–**2c**] are presented in Table 1.

**Table 1:** ^31^P{^1^H} NMR Data of M[**2a**–**2c**] in Et_2_O-*d*_10_ (with and without the Presence of Crown Ethers), CH_3_CN, and THF (**R**-P and Anionic P Notations Are in Accordance with Scheme 1)

δ ^31^P/ppm								
	**R**-P				anionic P			
compound	Et_2_O	CH_3_CN	THF	Et_2_O′^*a*^	Et_2_O	CH_3_CN	THF	Et_2_O′^a^
K[**2a**]	–12.1	–11.3	–28.1	–11.8	–76.1	–78.2	–72.1	–75.7
Li[**2b**]	–30.8	–31.8	–32.2	–30.6	–78.0	–79.4	–79.5	–77.6
K[**2c**]	18.1	11.6	–28	18.3	–74.1	–70.1	–72.1	–74.3

a Et_2_O′ indicates the ethereal solutions in the presence of crown ethers, 18-C-6 ([K(18-C-6)]**2a** and [K(18-C-6)]**2c**) or 12-C-4 ([Li(12-C-4)]**2b**).

The assignment of the two resonances to the P centers was straightforward, but it should be noted that in none of M[**2a**–**2c**] do the two inequivalent nuclei show evidence of P–P coupling across the rings. The composition of the product anions was also confirmed via negative ESI-MS experiments (Table S1 in the Supporting Information).

The intense blue colors observed for solids and solutions of M[**2a**–**2c**] correspond to single intense absorptions with λ_max_ ≈ 517 nm in Et_2_O (Figures S5a, S9a, and S13a in the Supporting Information). By contrast, when K[**2a**] was dissolved in CH_3_CN for voltammetry (*vide infra*), the blue color rapidly changed to yellow (λ_max_ ≈ 372 nm, Figure S5b in the Supporting Information).

**Figure 2:**
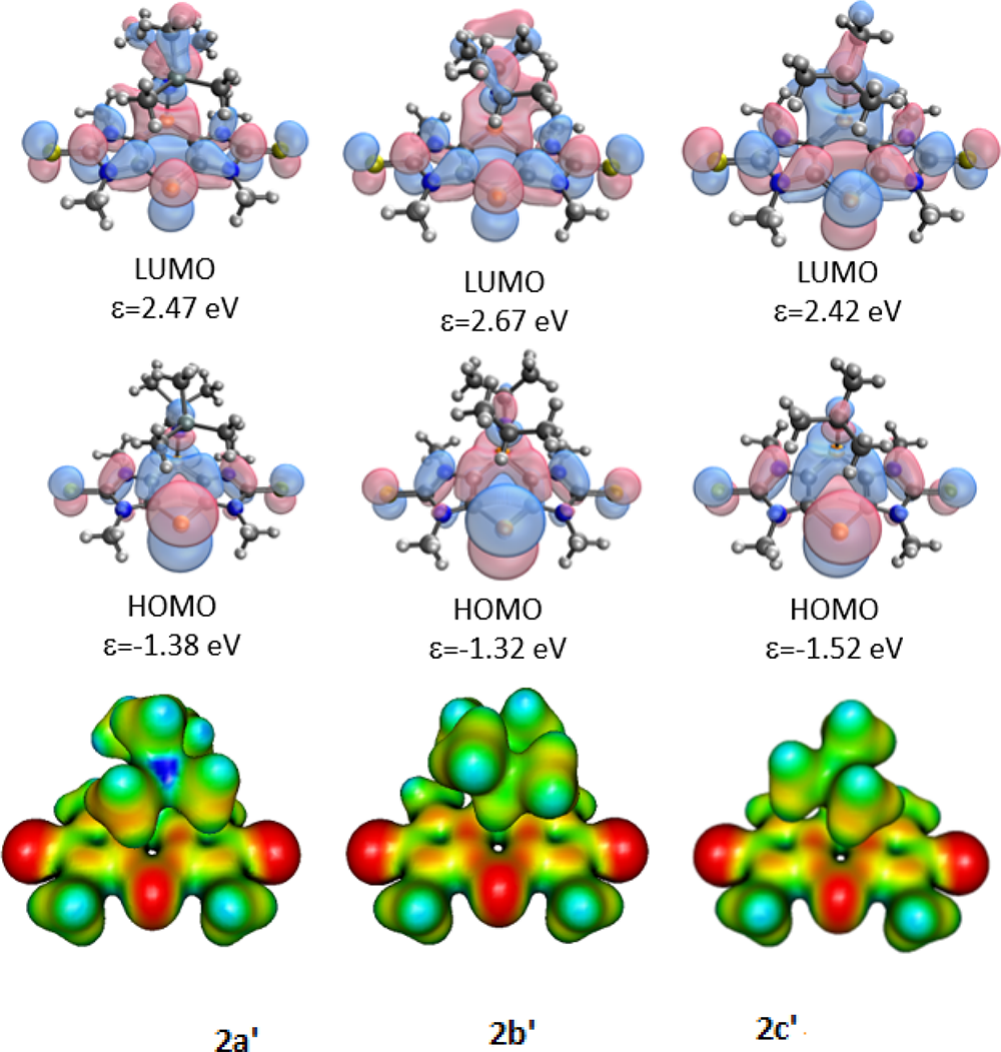
Kohn–Sham frontier orbitals, their energies (top), and electrostatic potential map (bottom; color code of electrostatic potential: red, <−0.1; yellow, −0.1 to −0.05; green, −0.05 to 0.05; light blue, 0.05–0.1; blue, >1.0).

To gain deeper insight into the electronic structures of **2a**–**2c**^–^, DFT calculations at the M06-2X/6-311+G** level of theory were performed on models wherein the *N*-^*n*^Bu groups are truncated to *N*-Me (indicated by ′). As expected, the middle rings of **2a′**–**2c′**^–^ exhibit lower aromatic character than in neutral **1a′** (NICS(0) values for **2a′**–**2c′**^–^ varied between −3.4 and −5.2; Tables S4, S8, and S12 in the Supporting Information), while the aromatic character of the outer ring remains high (NICS(0) varied between −8.8 and −9.4, Tables S4, S8, and S12 in the Supporting Information). The shapes and energies of the delocalized π frontier Kohn–Sham molecular orbitals (FMOs) are slightly affected by the variations in substituents (Figure 2). The HOMOs show large coefficients of the unsubstituted phosphorus on the center rings, but the sulfur p lone pairs have non-negligible contributions as well. On the electrostatic potential maps (Figure 2), the negative charges reside mainly on the unsubstituted phosphorus sites and the two sulfur atoms, in accordance with the HOMO coefficients. The TD-DFT calculations on **2a′**^–^ (maxima of the lowest excited states at 354 and 326 nm, Table 2) are in reasonably good agreement with the experimentally determined UV/vis absorption value in CH_3_CN but not with the deep blue colors observed in ether solvents (Et_2_O or THF). Presuming that the deep colors are from charge transfer (CT) bands associated with the formation of contact ion pairs due to the weaker solvation of K^+^ cations by ethers compared to CH_3_CN, additional TD-DFT calculations were performed on the optimized structures of the K^+^ salts (Table 2), which do indeed indicate transitions deep into the visible region. The charge transfer character of the transitions is clearly seen from the TD-DFT results. The lowest energy excitation of the calculated contact ion pair is the (anion-centered) HOMO-to-(K^+^ s-type centered) LUMO transition, indicative of CT. The similar ^31^P NMR shifts measured in Et_2_O and CH_3_CN (Table 1) and also the great similarity of the voltammetry results in THF (blue solutions) and CH_3_CN (yellow, *vide infra*) point to a small energy difference between the ether-solvated and ion-paired states.

**Table 2:** Important TD-DFT Results at the B3LYP/6-311G**//M06-2X/6-311+G** Level of Theory Calculated for **2a′**–**2c′**^–^ and the CAM-B3LYP/6-31G*//M06-2X/6-311+G** Level of Theory Calculated for the Contact Ion Pair M[**2a′–2c′**]

model	excited state	wavelength	oscillator strength	transition	contribution
**2a′^–^**	1	354 nm	0.1137	HOMO-LUMO	0.69524
	2	326 nm	0.1735	HOMO-1-LUMO	0.10945
				HOMO-LUMO+1	0.22399
				HOMO-LUMO+2	0.65285
K[**2a′**]	1	523 nm	0.0171	HOMO-LUMO	0.68460
				HOMO-LUMO+3	0.13519
**2b′^–^**	1	366 nm	0.1501	HOMO-LUMO	0.69593
	4	319 nm	0.0774	HOMO-LUMO+3	0.69343
Li[**2b′**]	1	393 nm	0.0231	HOMO-4-LUMO	0.15155
				HOMO-LUMO	0.68258
**2c′^–^**	1	354 nm	0.1235	HOMO-LUMO	0.69214
	4	294 nm	0.4978	HOMO-1-LUMO	0.65771
				HOMO-LUMO+1	0.17021
				HOMO-LUMO+2	0.12292
K[**2c′**]	1	419 nm	0.0178	HOMO-LUMO	0.67546
				HOMO-LUMO+4	–0.14105

To better support the notion that the color changes can be attributed to the presence/absence of CT bands, the encapsulation of K^+^ or Li^+^ by crown ether (18-crown-6 and 12-crown-4) was attempted. Indeed, in the presence of 18-crown-6 and 12-crown-4, compounds [K(18-C-6)]**2a** and [K(18-C-6)]**2c** could be isolated from Et_2_O as orange solids, but [Li(12-C-4)]**2b** remained purple and apparently showcased insignificant ^31^P NMR chemical shift changes in the cases of CH_3_CN and Et_2_O (Table 1).

**Figure 3:**
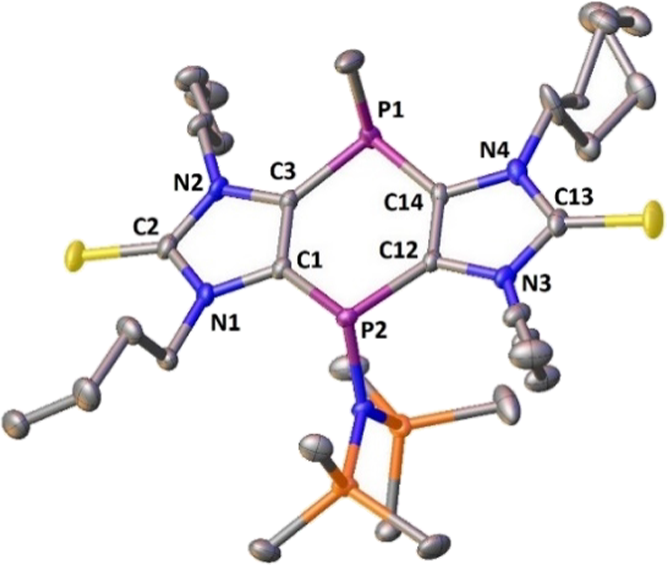
Molecular structure of *trans*-**3a**; hydrogen atoms are omitted for clarity (50% probability level). Selected bond lengths [Å] and angles [°]: P1-N5, 1.7161(16); P2-C29, 1.846(3); P1-C1, 1.8240(19); P1-C12, 1.816(2); P2-C3, 1.803(2); P2-C14, 1.8033(19); Σ < °P1 307.86 and Σ < °P2 297.08.

Reactions of salts M[**2a**–**2c**] with iodomethane at low temperatures yielded products **3a**–**3c** as clearly revealed by their ^31^P{^1^H} NMR spectra. All products were isolated as white powders (see the Supporting Information), and their constitutions were confirmed by NMR experiments (selected data are given in Table 3). While, for **3b** and **3c**, mixtures of *cis*/*trans* isomers (Scheme 1) were obtained, in the case of **3a** (see Table 3; for the structure of *trans*-**3a**, see Figure 3), only the *trans* product was detected. While it was surmised that the steric demand of the bis(trimethylsilyl)amino group leads to the selective formation of *trans*-**3a**, DFT calculations reveal the energy difference between the *cis* and *trans* isomers, Δ*E* (*cis*/*trans* values are 0.2, 1.1, and 0.9 kcal/mol for **3a′**, **3b′**, and **3c′**, respectively). In addition, very high and similar inversion barriers were determined (Figure S38 and Table S20 in the Supporting Information).

**Table 3:** ^31^P{^1^H} NMR Data (C_6_D_6_) of **3a**–**3c**

δ ^31^P/ppm					
	**R**-P	*CH*_3_-P	^3^*J*_P,P_/Hz	cis and trans ratio	Δ*E*(*cis*-*trans*)/kcal·mol^–1^
**3a**	–4.7	–72.3	16.6	only *trans* isomer	0.2
**3b**	–17.2 and −19.7	–75.2 and −69.3	9.1, 11.1	1:3.1	1.1
**3c**	25.6 and 26.5	–79.6 and −69.4	7.2, 13.4	1:4.1	0.9

Since all of these energy differences are small, it is possible that the selective formation of the *trans* isomer in the case of **3a** is due to kinetic control. This supposition is supported also by the calculated structures as salts K[**2a′**], K[**2b′**], and K[**2c′**], which are presented in the Supporting Information. Clearly, the bulky N(SiMe_3_)_2_ group occupies much more space below the central ring than the other two substituents, raising a barrier for the formation of the *cis* isomer. Single crystals of compound **3a**, suitable for X-ray diffraction analysis, were grown from a saturated diethyl ether solution.The structure confirmed the *trans* position of the amino and methyl groups at the central ring (Figure 3) having sums of angles at P1 and P2 of 307.9° and 297.1°, respectively. The endocyclic angle at C1-P2-C12 of 95.19(9)° and that at C3-P1-C14 of 96.11(9)° are rather acute.

**Scheme 2:**
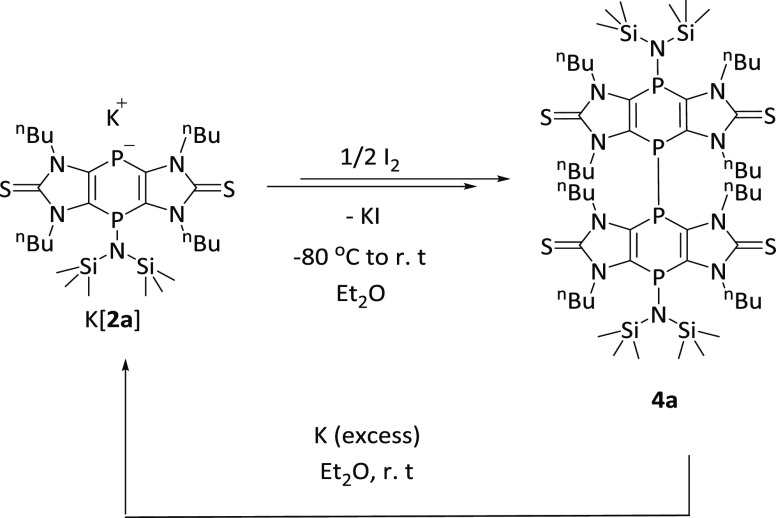
Oxidation of K[**2a**] to **4a** and Subsequent Reduction

Compound K[**2a**] was then selected for further chemical redox reactions because it has shown promising robustness under various reaction conditions. When an Et_2_O solution of I_2_ was added dropwise at −80 °C to a freshly prepared solution of K[**2a**] in Et_2_O (Scheme 2), a dark green color appeared and rapidly disappeared (within a few seconds) to finally give an orange solution containing product **4a** (42% yield).

The attribution of the transient green color to an intermediate free radical is supported by TD-DFT calculations on **2a′^•^** (Table S14 in the Supporting Information; lowest transition calculated at 1005 nm), while the orange color of **4** fits with calculations on **4a′** (computed as 509 nm).

The formation of **4a′** from two radicals was calculated to be exergonic (298 K, 1 bar), and the rather high reaction Gibbs free energy (28.7 kcal/mol) is in good agreement with the rapid changes in color. The reaction mixture was filtered via cannula to remove the KI salt and product **4a** isolated as an orange powder. The ^31^P{^1^H} NMR spectrum of **4a** (CDCl_3_) displays a pseudo-triplet signal at −0.4 (^3/4^*J*_P,P_ = 25.6 Hz, *P*-N(SiMe_3_)_2_) and −50.9 (^3/4^*J*_P,P_ = 25.6 Hz, P–P) ppm.

**Figure 4:**
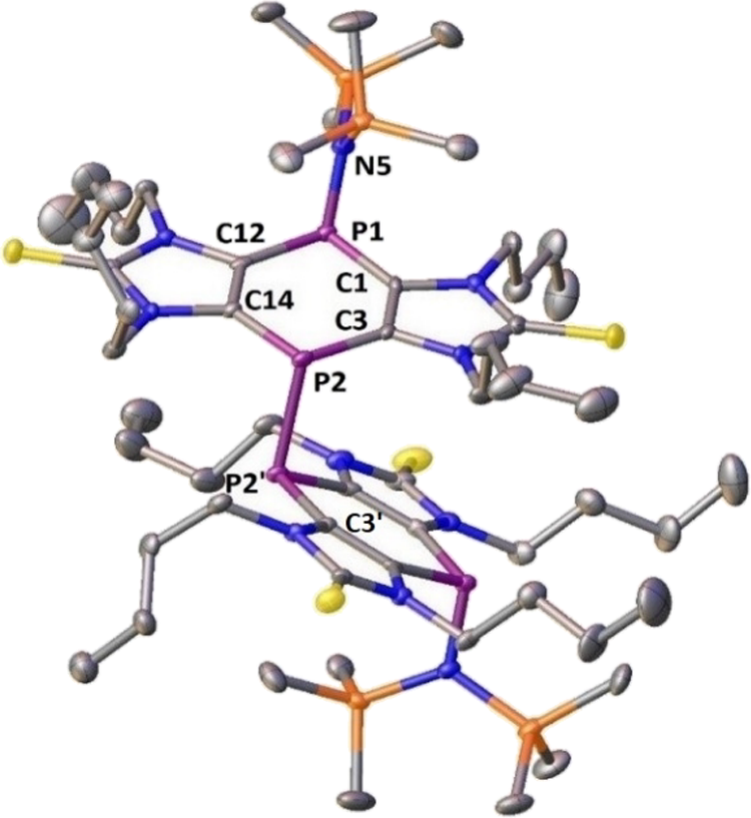
Molecular structure of compound **4a**; hydrogen atoms are omitted for clarity (50% probability level). Selected bond lengths [Å] and angles [°]: P1-C1, 1.807(4); P1-C12, 1.817(4); P2-C3, 1.791(4); P2-C14, 1.806(4); C12-C14, 1.360(5); C1-C3, 1.376(5); P1-N5, 1.718(3); P2-P2′, 2.303(2); C1-P1-C12, 94.43(18); C3-P2-C14, 96.40(18); Σ < °P1 304.48 and Σ < °P2 300.79.

Clear orange crystals of compound **4a**, suitable for X-ray diffraction analysis, were grown from a saturated diethyl ether solution (Figure 4). The analysis revealed a monoclinic crystal system with the space group *C*2/*c*. The structure shows a twisted arrangement along the P–P single bond. The C2-P2-P2′-C3′ torsion angle of 96.3° between the two tricyclic units is greater than the torsion angle observed previously for the sterically less demanding thiazol-2-thione-based tricycle.^21^ Most probably, also in this case, dispersion force-induced orientation of the two tricyclic units is present, which may also help in the aggregation and preorganization during the formation of the P–P bond.

To check if compound **4a** can be used to reform 2 equiv of K[**2a**] in a clean fashion, *i.e*., to formally reverse the oxidation with I_2_, compound **4a** was treated with an excess of potassium in Et_2_O at room temperature to avoid desulfurization of the thione functionality, which usually takes place at higher temperatures (Scheme 2). After 10 min of stirring, the color of the solution turned dark blue, indicating that the anionic species K[**2a**] was formed, which was additionally confirmed by the ^31^P{^1^H} NMR spectrum of the reaction mixture showing two singlets at −13 and −78 ppm.

**Figure 5:**
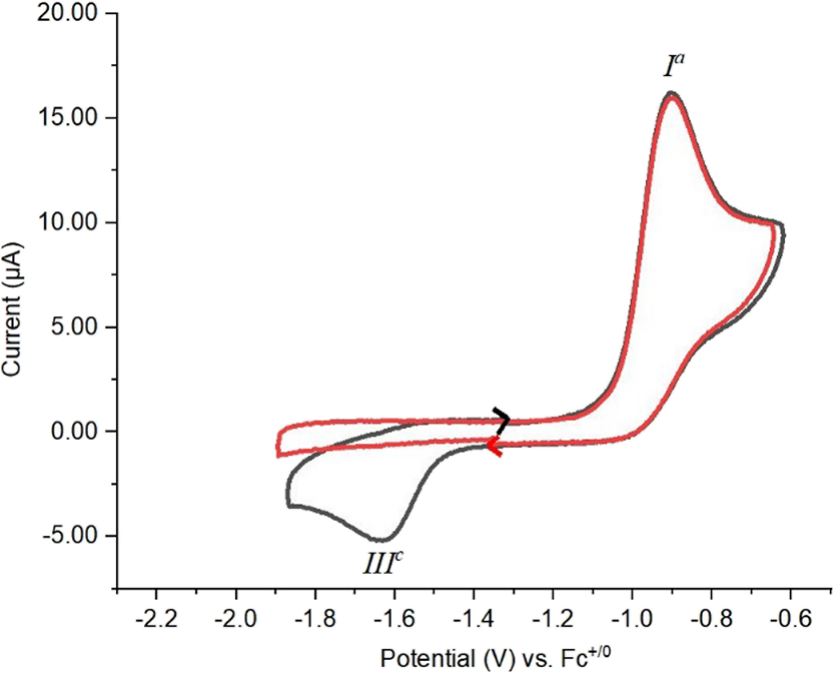
Cyclic voltammograms of K[**2a**] (2.59 mM) at a Pt electrode in a 0.1 M ^*n*^Bu_4_NPF_6_/CH_3_CN solution; red solid line, cathodic initial scan direction; black solid line, anodic initial scan direction; scan rates = 200 mV/s.

The redox chemistry of K[**2a**] and **4a** was subsequently investigated electrochemically via interfacial voltammetry at ceramic screen-printed Pt composite electrodes (also incorporating counter and Ag/AgCl solid-dot reference electrodes; details in the Supporting Information). Cyclic voltammetry (CV) on K[**2a**] in CH_3_CN/[^*n*^Bu_4_N][PF_6_] identifies a chemically irreversible (IRR) oxidation process *E*_p_^Ia^ = −0.90 V and a similarly chemically irreversible reduction process *E*_p_^IIIc^ = −1.63 V (Figure 5, blue trace) vs the ferrocene/ferrocenium redox couple (Fc^+/0^). But, when the initial scan direction was cathodic, no reduction signal occurs on the first cycle (Figure 5, red trace). Thus, the species responsible for *E*_p_^IIIc^ appears to be an electrolysis product of the process *E*_p_^Ia^.

The repeatability of the cyclic voltammograms was examined by carrying out multicycle experiments. Even after 50 cycles, the oxidation peak and reduction peak positions remain invariant and hardly any attenuation in peak intensities is observed (Figure S31a in the Supporting Information). The scan rate dependence was examined from 0.05 to 2.5 V/s with the expected current increase with scan rate and incremental increases in the potential peak positions as expected for IRR processes (Figure S31b in the Supporting Information and Table 4). The current ratios, moreover, remain quite similar over all the scan rates.

**Table 4:** Peak Potentials and Currents for Cyclic Voltammograms of K[**2a**] at Different Scan Rates^a^

scan rate (mV/s)	*E*_p_^IIIc^ (V)	*E*_p_^Ia^ (V)	*I*_p_^IIIc^ (μA)	*I*_p_^Ia^ (μA)	|*I*_p_^IIIc^/*I*_p_^Ia^|
2500	–1.81	–0.92	–19.52	57.28	0.34
1000	–1.79	–0.94	–10.37	30.17	0.34
500	–1.77	–0.95	–6.65	20.46	0.32
200	–1.63	–0.90	–5.20	16.20	0.32
50	–1.62	–0.91	–1.70	6.65	0.25

a Potentials are in V vs the Fc^+/0^ redox couple.

**Figure 6:**
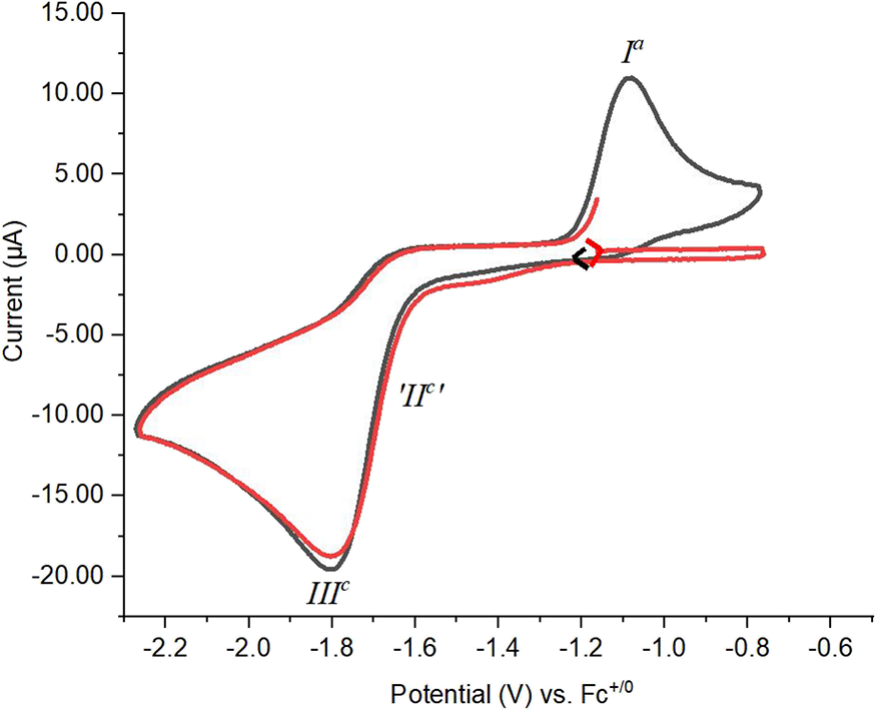
Cyclic voltammograms of **4a** (2.59 mM) at a Pt electrode in a 0.1 M ^*n*^Bu_4_NPF_6_/CH_3_CN solution; red solid line, anodic initial scan direction; black solid line, cathodic initial scan direction; scan rates = 200 mV/s.

CV experiments (Figure 6) were also conducted on solutions of **4a** under similar experimental conditions to K[**2a**]. The results appear as almost an inverse of the latter trace: a large, IRR reduction peak labeled *E*_p_^IIIc^ is found at −1.80 V and an equally IRR oxidation process labeled *E*_p_^Ia^ occurs at −1.08 V (Figure 6, blue trace) when the initial scan direction is cathodic. Scans starting in the anodic direction do not display *E*_p_^Ia^ in the first cycle (Figure 6, red trace). Notably, however, the current ratios of the two processes are distinctly different, with the relative size of *I*_p_^IIIc^ compared to the anodic peak appearing much larger in Figure 6 compared to Figure 5. Here too, the multicycle experiments corroborated the robust repeatability of the CV processes (Figure S32a in the Supporting Information), while variable scan rate experiments from 0.05 to 2.5 V/s also fit expectations for increased currents with scan rates and incrementing of the potentials with faster scans (Figure S32b in the Supporting Information and Table 5).

**Table 5:** Peak Potentials and Currents for Cyclic Voltammograms of **4a** at Different Scan Rates^a^

scan rate (mV/s)	*E*_p_^IIIc^ (V)	*E*_p_^Ia^ (V)	*I*_p_^IIIc^ (μA)	*I*_p_^Ia^ (μA)	|*I*_p_^Ia^/*I*_p_^IIIc^|
2500	–1.82	–0.85	–65.42	42.22	0.64
1000	–1.88	–0.90	–39.71	20.50	0.51
500	–1.85	–0.91	–27.60	13.99	0.50
200	–1.80	–1.08	–19.55	10.96	0.56
50	–1.73	–0.99	–8.45	3.69	0.43

a Potentials are in V vs the Fc^+/0^ redox couple.

These CV experiments, as voltammetric monitors, are fully consistent with the redox interconversion of K[**2a**] and **4a** already demonstrated in the chemical oxidation with I_2_ and reduction by elemental potassium. A plausible mechanism for the electrochemical processes based on the CV results is**:**
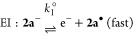

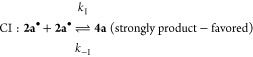

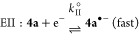

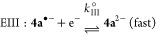

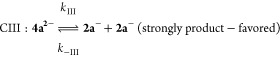


Here (according to the standard notation), E are electrochemical steps and C are (rapid, following) chemical steps. Process I represents the oxidation of K[**2a**] to form **4a** via dimerization of a short-lived P-centered radical species **2a**•. The reduction of **4a** involves cleavage of a P–P bond and is almost certainly a two-step process, denoted II and III. There is some evidence for process II in very fast scan rate cyclic voltammograms at ∼−1.6 V (see the Supporting Information), but under most CV conditions, II and III appear as a merged peak of double intensity, which accounts for the larger relative size of *I*_p_^IIIc^ when **4a** is the bulk analyte at the electrode interface.

Digital simulations of the cyclic voltammograms were undertaken using this common mechanism applied to K[**2a**] and **4a**, modifying only the analyte concentrations and the starting points and initial scan directions of the cyclic voltammograms (Figures S34 and S35 in the Supporting Information). Satisfactory agreement is obtained from such simulations when the forward rate constants for product formation, both *k*_III_ for the cleavage of **4a**^2–^ and *k*_I_ for fusing two **2a**^•^ to form the P–P bond, are much (10^6^ times) larger than the respective reverse reactions, at the relative applied potentials. Simulation, along with chemical redox cycling, provides strong support for robust redox shuttling between K[**2a**] and **4a**.

There is a strong need for new battery technologies to enable modern culture to address the impacts of energy and co-related climate challenges.^36^ The explosive growth of lithium-ion battery production and the ongoing strong demand for such devices have placed the sustainability of this technology under scrutiny.^37,38^ Lithium itself is a relatively scarce element—relative to the other alkali metals—but of major concern are the metals (Mn, Fe, Co, and Ni), especially Co, required for battery cathode construction.^39−41^ To the best of our knowledge, organophosphorus materials have not been considered for battery design. The low theoretical charge density of the **4a**/K[**2a**] redox shuttle and the rather negative cathode potential are obvious disadvantages, but further research in this direction may open the door to other phosphorus compounds with better properties.

An interesting feature of our system is its compatability with potassium, an attractive alternative to the current overdemand on Li.^42^ Considerable progress has already been reported for K/C_8_ (i.e., graphite intercalated) anodes.^43−45^ A consideration of cell potentials indicates that such anodes are at approximately −3.4 V vs Fc^+/0^, which, coupled with the average voltage of the **4a**/K[**2a**] redox cycle of −1.3 V, indicates an attractive nominal cell voltage approaching 2 V for the proposed cells, albeit that the whole redox cycle would operate at strongly negative potentials.^46^ Moreover, secondary cells based on this technology should be able to operate efficiently at ambient temperatures, unlike the elevated temperatures contemplated for sodium/sulfur battery technologies.^35^

## Conclusions

In this study, we have demonstrated the chemical two-electron switching between the anionic imidazole-2-thione-fused 1,4-dihydro-1,4-diphosphinine K[**2a**] and the P–P bonded oxidized dimeric form **4a**. Both K[**2a**] and **4a** show robust responses in multicycle cyclic voltammetry, which agree well with digital simulations. Based on the facile synthesis of the starting material (also in larger batches including tunability of N- and P-substituents) and the quantitative formation of their respective anions, these findings prompt us to propose this organophosphorus system as a potential cathode material for further research on the suitability of phosphorus compounds for secondary battery engineering.

## Supporting Information Available

General synthetic methods, NMR spectra, UV–Vis spectra, mass spectra, X-ray diffraction studies, electrochemistry experiments, digital CV simulations, and results of the TD-DFT calculations of compounds M[**2a′**–**2c′**] , anions **2a′**–**2c′**^–^ , compounds **4a′** and **2b′**, and free radical intermediates **2a′^•^**–**2c′^•^** (PDF)

## Accession Codes

CCDC 2001346–2001347 contain the supplementary crystallographic data for this paper. These data can be obtained free of charge via www.ccdc.cam.ac.uk/data_request/cif, or by emailing data_request@ccdc.cam.ac.uk, or by contacting The Cambridge Crystallographic Data Centre, 12 Union Road, Cambridge CB2 1EZ, UK; fax: +44 1223 336033.

## References

[ref1] Märkl G. 2,4,6-Triphenylphosphabenzene. Angew. Chem., Int. Ed. 1966, 5, 846–847. 10.1002/anie.196608463.

[ref2] Le Floch P. in Phosphorus-Carbon Heterocyclic Chemistry, ed. F., Mathey. Elsevier Science Ltd, Oxford 2001, 485–533.

[ref3] Dillon K. B.; Mathey F.; Nixon J. F. Phosphorus: the carbon copy: from organophosphorus to phospha-organic chemistry. John Wiley and Sons, Chichester 2001.

[ref4] Streubel R. 1λ^5^-Phosphinines. Sci. Synth. 2005, 1157–1179. 10.1055/sos-SD-015-01896.

[ref5] Black D. S. C.; Ihmels H.; Alvarez M.; Bergsträßer U.; Joule J. A. Science of Synthesis: Houben-Weyl Methods of Molecular Transformations Vol. 15: Six-Membered Hetarenes with One Nitrogen or Phosphorus Atom, Thieme. Thieme 2014.

[ref6] Märkl G.; Merz A. Ein Beitrag zum Problem der “nichtklassischen” Phosphabenzole. Tetrahedron Lett. 1968, 9, 3611–3614. 10.1016/S0040-4039(00)75513-4.

[ref7] Bruce M.; Meissner G.; Weber M.; Wiecko J.; Müller C. Lithium Salts of 2,4,6-Triaryl-λ^4^ -phosphinine Anions - A Comparison Study. Eur. J. Inorg. Chem. 2014, 2014, 1719–1726. 10.1002/ejic.201301259.

[ref8] Müller C.; Broeckx L. E. E.; de Krom I.; Weemers J. J. M. Developments in the Coordination Chemistry of Phosphinines. Eur. J. Inorg. Chem. 2013, 2013, 187–202. 10.1002/ejic.201200912.

[ref9] Pfeifer G.; Chahdoura F.; Papke M.; Weber M.; Szűcs R.; Geffroy B.; Tondelier D.; Nyulászi L.; Hissler M.; Müller C. Synthesis, Electronic Properties and OLED Devices of Chromophores Based on λ^5^-Phosphinines. Chem. Eur. J. 2020, 26, 10534–10543. 10.1002/chem.202000932. 32092780 PMC7496645

[ref10] Regulska E.; Hindenberg P.; Romero-Nieto C. From Phosphaphenalenes to Diphosphahexaarenes: An Overview of Linearly Fused Six-Membered Phosphorus Heterocycles. Eur. J. Inorg. Chem. 2019, 2019, 1519–1528. 10.1002/ejic.201801340.

[ref11] Hay C.; Hissler M.; Fischmeister C.; Rault-Berthelot J.; Toupet L.; Nyulászi L.; Réau R. Phosphole-Containing π-Conjugated Systems: From Model Molecules to Polymer Films on Electrodes. Chem. Eur. J. 2001, 7, 4222–4236. 10.1002/1521-3765(20011001)7:19<4222::AID-CHEM4222>3.0.CO;2-3. 11686602

[ref12] Baumgartner T.; Réau R. Organophosphorus π-conjugated materials. Chem. Rev. 2006, 106, 4681–4727. 10.1021/cr040179m. 17091932

[ref13] Joly D.; Tondelier D.; Deborde V.; Delaunay W.; Thomas A.; Bhanuprakash K.; Geffroy B.; Hissler M.; Réau R. White Organic Light-Emitting Diodes Based on Quench-Resistant Fluorescent Organophosphorus Dopants. Adv. Funct. Mater. 2012, 22, 567–576. 10.1002/adfm.201102005.

[ref14] Shameem M. A.; Orthaber A. Organophosphorus Compounds in Organic Electronics. Chem. Eur. J. 2016, 22, 10718–10735. 10.1002/chem.201600005. 27276233

[ref15] Larrañaga O.; Romero-Nieto C.; de Cózar A. Dismantling the Hyperconjugation of π-Conjugated Phosphorus Heterocycles. Chem. – Eur. J. 2019, 25, 9035–9044. 10.1002/chem.201900225. 31033030

[ref16] Kobayashi Y.; Kumadaki I.; Ohsawa A.; Hamana H. 2,3,5,6-Tetrakis (trifluoromethyl)-1,4-diphosphabenzene. Tetrahedron Lett. 1976, 17, 3715–3716. 10.1016/S0040-4039(00)93089-2.

[ref17] Downie I. M.; Lee J. B.; Matough M. F. S. The reaction of alcohols with carbon tetrachloride and phosphorus trisdimethylamide. Chem. Commun. 1968, 1350b 10.1039/C1968001350B.

[ref18] Kobayashi Y.; Hamana H.; Fujino S.; Ohsawa A.; Kumadaki I. Studies on organic fluorine compounds. 28. Synthesis and some reactions of tetrakis(trifluoromethyl)-1,4-diphosphabenzene. J. Am. Chem. Soc. 1980, 102, 252–255. 10.1021/ja00521a039.

[ref19] Kobayashi Y.; Fujino S.; Kumadaki I. Syntheses of trifluoromethylated thiadiphosphanorbornadiene and thiadiphosphole. J. Am. Chem. Soc. 1981, 103, 2465–2466. 10.1021/ja00399a079.

[ref20] Koner A.; Pfeifer G.; Kelemen Z.; Schnakenburg G.; Nyulászi L.; Sasamori T.; Streubel R. 1,4-Diphosphinines from Imidazole-2-thiones. Angew. Chem., Int. Ed. 2017, 56, 9231–9235. 10.1002/anie.201704070. 28586154

[ref21] Begum I.; Schnakenburg G.; Kelemen Z.; Nyulászi L.; Boeré R. T.; Streubel R. Expanding the chemistry of ring-fused 1,4-diphosphinines by stable mono anion formation. Chem. Commun. 2018, 54, 13555–13558. 10.1039/C8CC08158A. 30444243

[ref22] Koner A.; Kunz M.; Schnakenburg G.; Streubel R. The Quest for Twofold Reductive P-C Bond Cleavage of *P*-Ph Substituted 1,4-Dihydro-1,4-diphosphinine Derivatives. Eur. J. Inorg. Chem. 2018, 2018, 3778–3784. 10.1002/ejic.201800753.

[ref23] Koner A.; Kelemen Z.; Schnakenburg G.; Nyulászi L.; Streubel R. 1,4-Additions of tricyclic 1,4-diphosphinines - a novel system to study σ-bond activation and π-π dispersion interactions. Chem. Commun. 2018, 54, 1182–1184. 10.1039/C7CC09349G. 29328346

[ref24] Koner A.; Gabidullin B. M.; Kelemen Z.; Nyulászi L.; Nikonov G. I.; Streubel R. 7-Metalla-1,4-diphosphanorbornadienes: cycloaddition of monovalent group 13 NacNac complexes to a stable 1,4-diphosphinine. Dalton Trans. 2019, 48, 8248–8253. 10.1039/C9DT01425J. 31094383

[ref25] Miyake H.; Sasamori T.; Wu J. I.-C.; Schleyer P. v. R.; Tokitoh N. The 4,5,6-triphospha[3]radialene dianion: a phosphorus analogue of the deltate dianion. A NICS(0)_πzz_ examination of their aromaticity. Chem. Commun. 2012, 48, 11440–11442. 10.1039/c2cc35978b. 23090183

[ref26] Miyake H.; Sasamori T.; Tokitoh N. Synthesis and properties of 4,5,6-triphospha[3]radialene. Angew. Chem., Int. Ed. 2012, 51, 3458–3461. 10.1002/anie.201200374. 22359370

[ref27] Sasamori T.; Tokitoh N.; Streubel R. π-Electron Redox Systems of Heavier Group 15 Elements, in Organic Redox Systems, Synthesis, Properties and Applications (ed. T., Nishinaga). Wiley, Hoboken, New Jersey 2016, 563–578.

[ref28] Shah S.; Burdette S. C.; Swavey S.; Urbach F. L.; Protasiewicz J. D. Alkali Metal Induced Rupture of a Phosphorus–Phosphorus Double Bond. Electrochemical and EPR Investigations of New Sterically Protected Diphosphenes and Radical Anions [ArPPAr]^•-^. Organometallics 1997, 16, 3395–3400. 10.1021/om970025f.

[ref29] Tohmé A.; Grelaud G.; Argouarch G.; Roisnel T.; Labouille S.; Carmichael D.; Paul F. Redox-induced reversible P-P bond formation to generate an organometallic σ^4^λ^4^-1,2-biphosphane dication. Angew. Chem., Int. Ed. 2013, 52, 4445–4448. 10.1002/anie.201208682. 23495219

[ref30] Lejeune M.; Grosshans P.; Berclaz T.; Sidorenkova H.; Besnard C.; Pattison P.; Geoffroy M. Role of the aromatic bridge on radical ions formation during reduction of diphosphaalkenes. New J. Chem. 2011, 35, 2510 10.1039/c1nj20314b.

[ref31] Märkl G.; Kreitmeier P.; Daffner R. Oxidation von 4-N,N-dimethylaminophenyl-1-phosphabutatrienen zu diphosphanen mit “Malachitgrün”-Chromophoren. Tetrahedron Lett. 1993, 34, 7045–7048. 10.1016/S0040-4039(00)61593-9.

[ref32] Manan N. S. A.; Aldous L.; Alias Y.; Murray P.; Yellowlees L. J.; Lagunas M. C.; Hardacre C. Electrochemistry of sulfur and polysulfides in ionic liquids. J. Phys. Chem. B 2011, 115, 13873–13879. 10.1021/jp208159v. 21992687

[ref33] Evans A.; Montenegro M. I.; Pletcher D. The mechanism for the cathodic reduction of sulphur in dimethylformamide: low temperature voltammetry. Electrochem. Commun. 2001, 3, 514–518. 10.1016/S1388-2481(01)00203-X.

[ref34] Yamin H.; Gorenshtein A.; Penciner J.; Sternberg Y.; Peled E. Lithium Sulfur Battery: Oxidation/Reduction Mechanisms of Polysulfides in THF Solutions. J. Electrochem. Soc. 1988, 135, 1045–1048. 10.1149/1.2095868.

[ref35] Steudel R.; Chivers T. The role of polysulfide dianions and radical anions in the chemical, physical and biological sciences, including sulfur-based batteries. Chem. Soc. Rev. 2019, 48, 3279–3319. 10.1039/C8CS00826D. 31123728

[ref36] Armand M.; Tarascon J.-M. Building better batteries. Nature 2008, 451, 652–657. 10.1038/451652a. 18256660

[ref37] Dai Q.; Kelly J. C.; Gaines L.; Wang M. Life Cycle Analysis of Lithium-Ion Batteries for Automotive Applications. Batteries 2019, 5, 48 10.3390/batteries5020048.

[ref38] Aichberger C.; Jungmeier G. Environmental Life Cycle Impacts of Automotive Batteries Based on a Literature Review. Energies 2020, 13, 6345 10.3390/en13236345.

[ref39] Massé R. C.; Liu C.; Li Y.; Mai L.; Cao G. Energy storage through intercalation reactions: electrodes for rechargeable batteries. Natl. Sci. Rev. 2017, 4, 26–53. 10.1093/nsr/nww093.

[ref40] Liu C.; Neale Z. G.; Cao G. Understanding electrochemical potentials of cathode materials in rechargeable batteries. Mater. Today 2016, 19, 109–123. 10.1016/j.mattod.2015.10.009.

[ref41] Tarascon J. M.; Armand M. Issues and challenges facing rechargeable lithium batteries. Nature 2001, 414, 359–367. 10.1038/35104644. 11713543

[ref42] Bhide A.; Hofmann J.; Dürr A. K.; Janek J.; Adelhelm P. Electrochemical stability of non-aqueous electrolytes for sodium-ion batteries and their compatibility with Na(0.7)CoO_2_. Phys. Chem. Chem. Phys. 2014, 16, 1987–1998. 10.1039/C3CP53077A. 24336408

[ref43] Kapaev R. R.; Troshin P. A. Organic-based active electrode materials for potassium batteries: status and perspectives. J. Mater. Chem. A 2020, 8, 17296–17325. 10.1039/D0TA04741D.

[ref44] Carboni M.; Naylor A. J.; Valvo M.; Younesi R. Unlocking high capacities of graphite anodes for potassium-ion batteries. RSC Adv. 2019, 9, 21070–21074. 10.1039/C9RA01931F. PMC9065985 35515520

[ref45] Komaba S.; Hasegawa T.; Dahbi M.; Kubota K. Potassium intercalation into graphite to realize high-voltage/high-power potassium-ion batteries and potassium-ion capacitors. Electrochem. Commun. 2015, 60, 172–175. 10.1016/j.elecom.2015.09.002.

[ref46] Hodge S. A.; Tay H. H.; Anthony D. B.; Menzel R.; Buckley D. J.; Cullen P. L.; Skipper N. T.; Howard C. A.; Shaffer M. S. P. Probing the charging mechanisms of carbon nanomaterial polyelectrolytes. Faraday Discuss. 2014, 172, 311–325. 10.1039/C4FD00043A. 25427072

