# Supplementary material for: Reversible Redox Chemistry of Anionic Imidazole-2-thione-Fused 1,4-Dihydro-1,4-diphosphinines
Source: Inorg Chem. 2022 Mar 8;61(11):4639–46. doi: 10.1021/acs.inorgchem.1c03620 (PMC8941515; doi:10.1021/acs.inorgchem.1c03620)
Supplement: Supplementary file 1 — ic1c03620_si_001.pdf [file ic1c03620_si_001.pdf]

Supplementary material

Reversible redox chemistry of anionic imidazole-2-thione-fused 1,4-dihydro-1,4-diphosphinines

*Mridhul. R. K. Ramachandran,[a] Gregor Schnakenburg,[a] Moumita Majumdar,[b] Zsolt Kelemen,[c] Dalma Gál,[c] Laszlo Nyulászi,[*c] René. T. Boeré,[*d] and Rainer. K. Streubel[*a]*

[a.]Institut für Anorganische Chemie, Rheinische Friedrich-Wilhelms-Universität Bonn, Gerhard-Domagk-Straße 1, D-53121 Bonn, Germany. E-mail: r.streubel@uni-bonn.de.

[b.]Department of Chemistry, Indian Institute of Science Education and Research, Pune-411008, Maharashtra, India. E-mai: moumitam@iiserpune.ac.in.

[c].Department of Inorganic and Analytical Chemistry, and MTA-BME Computation Driven Chemistry Research Group, Budapest University of Technology and Economics Szt Gellert ter 4, 1111 Budapest (Hungary). E-mail: nyulaszi@mail.bme.hu.

[d.]Department of Chemistry and Biochemistry, University of Lethbridge, 4401 University Drive West, Lethbridge, AB, T1K3M4, Canada. E-mail: boere@uleth.ca.

* Corresponding author. Email: r.streubel@uni-bonn.de

Contents

1.Experimental Section……………………………………………………………………………………2

2.Spectra for compound K[2a]…………………………………………………………………………5

3.Spectra for compound Li[2b]…………………………………………………………………………8

4.Spectra for compound K[2c]…………………………………………………………………………10

5.Spectra for compound 3a……………………………………………………………………………12

6.Spectra for compound 3b……………………………………………………………………………14

7.Spectra for compound 3c……………………………………………………………………………16

8.Spectra for compound 4a……………………………………………………………………………17

9.X-ray diffraction studies……………………………………………………………………………21

10.Electrochemistry experiments and supplementary results……………………………………22

11. Computational Details……………………………………………………………………………26

1. Experimental Section

General considerations

All manipulations and reactions of air and moisture sensitive compounds were performed under inert gas atmosphere (argon) using standard Schlenk line apparatus or working in a glove box. Removal of oxygen traces from Argon gas was done by a copper catalyst (BTS) while silica gel and phosphorus pentoxide was used for drying. Purification of solvents was ensured by boiling them over Na wire and benzophenone (in the case of dichloromethane over calcium hydride) under argon atmosphere prior to use. A MAT 90 or MAT 95 XL spectrometer (70 eV) was used to record electron ionization (EI) mass spectra. The progress of the reactions was monitored via $^{31}P\{^1H\}$ NMR spectroscopy. NMR measurements were performed on a Bruker AVI (300 MHz for ^1H) and Bruker AVI (400 MHz for ^1H). 85% H_3PO_4 was used as external standard for ^{31}P NMR spectra while 1H and ^{13}C NMR spectra were referenced to the residual protons of the deuterated solvents. UV/Vis spectroscopic measurements were done using a UV-1650PC Shimadzu, while infrared spectra were recorded on a Nicolet 380 (FT-IR) instrument. For single crystal X-ray diffraction studies a Bruker D8-Venture diffractometer was used. Crystallographic data for the structures reported in this paper have been deposited with the Cambridge Crystallographic Data Centre as supplementary publication no. CCDC-2001346 (**3a**), CCDC-2001347 (**4a**) which can be obtained free of charge via www.ccdc.cam.ac.uk/data_request/cif.

General synthetic method for M[2a-c] :
To 1,4-diphosphinine **1** (100 mg, 0. 206 mmol) the nucleophile (each 0.206 mmol) was added (in a Schlenk tube) followed by the addition of 3mL of dry Et_2O under ambient conditions. A rapid colour change was observed from red to bluish-violet. The reaction mixture was then stirred for an additional hour at room, the solvent then removed under reduced pressure (3.8 x 10^{-2} mbar) to obtain blue-violet powders. The raw products M[**2a-c**] were washed with *n*-pentane (3 x 2 mL) to remove other impurities and then dried under reduced pressure for an hour (3.8 x 10^{-2} mbar).

K[**2a**]

Yield (without coordinated solvent) : 78 mg (0.11 mmol, 53 %), deep-blue powder. ^1H NMR (300 MHz, Et_2O-*d*10): δ = -0.38-0.57 (m, 18H, *N-SiMe₃*), 0.95-1.08 (m, 1H, $^3J_{H,H}$ = 7.3 Hz, $NCH_2CH_2CH_2$*Me*), 1.39-1.57 (m, 8H, NCH_2CH_2*CH_2*Me), 1.81-1.99 (m, 8H, NCH_2*CH_2*CH_2Me), 3.78-3.92 (m, 2H, N*CH_2*CH_2CH_2Me), 4.01-4.16 (m, 2H, N*CH_2*CH_2CH_2Me), 4.27-4.43 (m, 2H, N*CH_2*CH_2CH_2Me), 4.54-4.74 (m, 2H, N*CH_2*CH_2CH_2Me). ^{13}C NMR (75 MHz, Et_2O-*d*10): δ = 0.34 (br s, *N-SiMe_3*), 1.6 (br s, *N-SiMe_3*), 20 & 20.1 (s, $NCH_2CH_2CH_2$*Me*), 29.6 & 31.3 (s, NCH_2CH_2*CH_2*Me), 30.6 (br, NCH_2*CH_2*CH_2Me), 45.0 (d, $^3J_{P,C}$ = 15.0 Hz, N*CH_2*CH_2CH_2Me), 45.6 (d, $^3J_{P,C}$ = 9.6 Hz, N$CH_2CH_2CH_2$Me), 117.2 (broad s, *P-C* of the middle ring), 160.9 (s, *C=S*). $^{31}P\{^1H\}$-NMR (121.5 Hz, Et_2O-*d*10): δ = -12.1 (s, *P*-N($SiMe_3)_2$), -77.9 (s, anionic P). Neg. ESI-MS: for $C_{28}H_{54}N_5P_2S_2Si_2$ theor./exp. 642.2838/642.2840. IR: ṽ (cm^{-1}) = 2960 (*w*), 2929 (*w*), 2864 (*w*), 1400 (*w*), 1380 (*s*), 1250 (*m*), 1210 (*m*), 870 (br. *s*). UV/Vis (Et_2O): λ_{max} in nm (ε in $Lmol^{-1}cm^{-1}$) = 517 (ε = 1580), 385 (ε = 650), 327 (ε = 1370). EA (without coordinated solvent, expected chemical formula : $C_{28}H_{54}N_5P_2S_2Si_2K$): exp. C 48.97, H 7.88, N 9.84, S 9.31; Calc. C 49.30, H 7.97, N 10.26, S 9.40.

Li[**2b**]

Yield (without coordinated solvent) : 75 mg (0.13 mmol, 63 %), deep-blue powder. ^1H NMR (300 MHz, Et_2O-*d*10): δ = 0.93 (t, 12H, $^2J_{H,H}$ = 7.4 Hz, $NCH_2CH_2CH_2$*Me*), 0.97 (d, 12H, $^2J_{H,H}$ = 6.1 Hz, *N-^{i}Pr*, N-CH_2-((*CH_3*)$_2$)$_2$), 1.94-2.15 (m, 8H, NCH_2CH_2*CH_2*Me), 2.92-3.15 (m, 2H, *N-^{i}Pr*, N-*CH_2*-Me_2), 3.19-3.38 (m, 4H, NCH_2*CH_2*CH_2Me), 3.53-3.79 (m, 4H, NCH_2*CH_2*CH_2Me), 4.10-4.18 (m, 2H, N*CH_2*CH_2CH_2Me), 3.88-4.07 (m, 2H, N*CH_2*CH_2CH_2Me), 4.12-4.41 (m, 2H, N*CH_2*CH_2CH_2Me), 4.62-5.0 (m, 2H, N*CH_2*CH_2CH_2Me). ^{13}C NMR (75 MHz, Et_2O-*d*10): δ = 17.6 (s, $NCH_2CH_2CH_2$*Me*), 20.6 (s, *N-^{i}Pr*, N-CH_2-((*CH_3*)$_2$)$_2$), 27.5 (s, NCH_2CH_2*CH_2*Me), 29 (s, NCH_2*CH_2*CH_2Me), 42.6 (s, N*CH_2*CH_2CH_2Me), 43.0 (m, *N-^{i}Pr*, N-*CH_2*-Me_2), 115.7 (br s, *P-C* of the middle ring), 156.8 (s, *C=S*). $^{31}P\{^1H\}$-NMR (121.5 MHz, Et_2O-*d*10): δ = -30.8 (s, *P*-N^{i}Pr), -78 (s, anionic P). neg-ESI-MS: m/z (%) = 563.167 (100), $[C_{27}H_{43}N_5P_2S_2]^+$, 614.287 (90), $[M+O_2]^+$. IR: ṽ (cm^{-1}) = 2960 (*w*), 2929 (*w*), 2864 (*w*), 1442 (*m*), 1408 (*s*), 1363 (*m*), 1217 (*s*), 1172 (*w*), 1150 (*w*), 879 (*s*), 770 (*m*), 666 (*w*). UV/Vis (Et_2O): λ_{max} in nm (ε in $Lmol^{-1}cm^{-1}$) = 517 (ε = 16690), 387 (ε = 5280), 350 (ε = 3540). EA (without coordinated solvent, expected chemical formula : $C_{28}H_{50}N_5P_2S_2Li$): exp. C 55.64, H 8.21, N 10.95, S 10.89 ; Calc. C 57.02, H 8.55, N 11.88, S 10.87.

K[**2c**]

Yield (without coordinated solvent) : 78 mg (0.13 mmol, 63 %), deep-blue powder. ^1H NMR(300 MHz, Et_2O-*d*10): δ = 1.0 (t, 12H, $^3J_{H,H}$ = 7.4 Hz, $NCH_2CH_2CH_2$*Me*), 1.1 (s, 9H, *O-^{t}Bu*), 1.38-1.52 (m, 8H, NCH_2CH_2*CH_2*Me), 1.78-1.97 (m, 8H, NCH_2*CH_2*CH_2Me), 4.07-4.21 (m, 4H, N*CH_2*CH_2CH_2Me), 4.22-4.31 (m, 2H, N*CH_2*CH_2CH_2Me), 4.61-4.72 (m, 2H, N*CH_2*CH_2CH_2Me). ^{13}C NMR (75 MHz, Et_2O-*d*10): δ = 20.6 & 20.8 (s, $NCH_2CH_2CH_2$*Me*), 29.8 (d, $J_{P,C}$ = 4.1Hz, NCH_2CH_2*CH_2*Me), 30.8 (d, $J_{P,C}$ = 6.8 Hz, NCH_2*CH_2*CH_2Me), 31.5 (m, NCH_2*CH_2*CH_2Me), 46.0 (d, $^3J_{P,C}$ = 10.6 Hz, N*CH_2*CH_2CH_2Me), 46.4 (d, $^3J_{P,C}$ = 14.3 Hz, N*CH_2*CH_2CH_2Me), 74.8 (s, *O-C*($CH_3)_3$), 120.5 (d, $J_{P,C}$ = 4.8 Hz, *P-C* of the middle ring), 120.4 (d, $J_{P,C}$ = 4.6 Hz, *P-C* of the middle ring), 164.2 (br, *C=S*). $^{31}P\{^1H\}$-NMR (Et_2O-*d*10): δ = 18.3 (s, *P*-O-^{t}Bu), -74.1 (s,

anionic P). neg-ESI-MS: m/z (%) = 499.189 (100), $[C_{22}H_{37}N_4OP_2S_2]^+$, 500.192 (26), $[C_{22}H_{38}N_4OP_2S_2]^+$. IR: ṽ ($cm^{-1}$) = 2963 (*w*), 2934 (*w*), 2870 (*w*), 1441 (*m*), 1402 (*s*), 1363(*m*), 1291(*w*), 1258 (*w*), 1217 (*m*), 1170 (*w*), 1150 (*w*), 773 (*m*), 669 (*m*), 628 (*w*). UV/Vis (Et_2O): λ_{max} in nm (ε in $Lmol^{-1}cm^{-1}$) = 517 (ε = 6260), 364 (ε = 4240). EA (without coordinated solvent, expected chemical formula : $C_{26}H_{45}N_4OP_2S_2K$): exp. C 49.34, H 7.40, N 9.14, S 10.58; Calc. C 52.49, H 7.62, N 9.41, S 10.78.

General synthetic method for 3a-c:

1,4-Diphosphinine **1** (100 mg, 0.206 mmol) was taken in a Schlenk tube and the base (KHMDS for **3a**, LDA for **3b** and KO*t*Bu for **3c**) (0.206 mmol) was added into it followed by the addition of 3mL dry Et_2O. A rapid colour change was observed from red to bluish violet. Then, the reaction mixture was stirred for an hour at room temperature. Then, the reaction mixture was brought to -80 °C by keeping it in Dewar bath. Then, methyl iodide (13μL, 0.206 mmol) was added into the reaction mixture, dropwise using a micro-syringe. After an hour, colour change was observed from bluish violet to bright orange. The colour became paler when it was kept for further 4 hours more. After stirring the reaction mixture for overnight at room temperature obtained white turbid solutions, in all the cases from **3a**-**3c**. The reaction mixture was filtered through a silica bed to remove the KI salt and concentrated under reduced pressure (6.3 x 10^{-2}) to get the compound **3a-3c** as white powders.

3a

Yield : 47.4 mg (0.07 mmol, 35 %), white powder. M.p. 139 °C. ^{1}H NMR(300 MHz, C_6D_6) : δ = -0.26 (s, 9H, *N-SiMe$_3$*), 0.31 (t, 9H, $^4J_{P,H}$ = 2.7 Hz, *N-SiMe$_3$*), 0.78 (d, $^3J_{H,H}$ = 5.1 Hz, *P-Me*), 0.84 (t, 6H, $^3J_{H,H}$ = 7.3 Hz, $NCH_2CH_2CH_2$*Me*), 0.88 (t, 6H, $^3J_{H,H}$ = 7.5 Hz, $NCH_2CH_2CH_2$*Me*), 1.24-1.48 (m, 8H, NCH_2CH_2*CH$_2$*Me), 1.85-2.26 (m, 8H, NCH_2*CH$_2$*CH_2Me), 3.55-3.63 (m, 2H, N*CH$_2$*CH_2CH_2Me), 3.98-4.06 (m, 2H, N*CH$_2$*CH_2CH_2Me), 4.61-4.68 (m, 2H, N*CH$_2$*CH_2CH_2Me), 4.91-4.99 (m, 2H, N*CH$_2$*CH_2CH_2Me). ^{13}C NMR (75 MHz, C_6D_6) : δ = 2.9 (s, *N-SiMe$_3$*), 4.6 (d, $^2J_{P,C}$ = 14.7 Hz, *N-SiMe$_3$*), 13.4 & 13.6 (s, $NCH_2CH_2CH_2$*Me*), 16.3 (dd, $J_{P,C}$ = 18.2 Hz, $J_{P,C}$ = 12.2 Hz, *P-Me*), 19.9 & 20.2 (s, NCH_2CH_2*CH$_2$*Me), 30.6 (br, NCH_2*CH$_2$*CH_2Me), 46.9 (d, $^3J_{P,C}$ = 5.7 Hz, N*CH$_2$*CH_2CH_2Me), 47.3 (d, $^3J_{P,C}$ = 8.6 Hz, N$CH_2CH_2CH_2$Me), 126.5 (d, $J_{P,C}$ = 1.5 Hz, *P-C* of the middle ring), 128.6 (dd, $J_{P,C}$ = 16.8 Hz , $J_{P,C}$ = 2.8 Hz, *P-C* of the middle ring), 168.5 (br, *C=S*). ^{31}P{^{1}H}-NMR (121.5 Hz, C_6D_6) : δ = -4.7 (d, $^3J_{P,P}$ = 16.6 Hz, *P*-N($SiMe_3$)$_2$), -72.3 (d, $^3J_{P,P}$ = 16.6 Hz, *P*-Me). EI-MS (70 eV): m/z (%) = 147 (100), $[C_5H_{17}NSi_2]^+$, 657.0 (25), $[M]^+$. HRMS: for $C_{29}H_{57}N_5P_2S_2Si_2$ theor./exp. 657.3069/657.3069. IR : ṽ (cm^{-1}) = 2956 (*w*), 2929 (*w*), 2861 (*w*), 1437 (*m*), 1402 (*s*), 1253 (*m*), 1217 (*m*), 887 (*s*), 871(*s*), 842 (*s*). EA: exp. C 52.71, H 8.70, N 10.58, S 9.45. Calc. C 52.93, H 8.73, N 10.64, S 9.74.

3b

Yield: 49.26 mg (0.08 mmol, 40 %), white powder. M.p. 115 °C. ^{1}H NMR(300 MHz, C_6D_6) : δ = 0.87 (m, 12H, $NCH_2CH_2CH_2$*Me*), 1.2-1.4 (m, 12H, *N-^{i}Pr*, N-CH_2-((CH_3)$_2$)$_2$), 1.59 (d, $^3J_{H,H}$ = 14.6 Hz, *P-Me*), 1.68-1.82 (m, 4H, NCH_2CH_2*CH$_2$*Me), 1.94-2.10 (m, 4H, NCH_2CH_2*CH$_2$*Me), 3.22-3.34 (m, 2H, *N-^{i}Pr*, N-*CH$_2$*-Me_2), 3.9-4.1 (m, 4H, NCH_2*CH$_2$*CH_2Me), 4.13-4.31 (m, 4H, NCH_2*CH$_2$*CH_2Me), 4.32-4.43 (m, 4H, N*CH$_2$*CH_2CH_2Me), 4.63-4.9 (m, 4H, N*CH$_2$*CH_2CH_2Me). ^{13}C NMR (75 MHz, C_6D_6) : δ = 13.9&14 (s, $NCH_2CH_2CH_2$*Me*), 20.4 (d, $J_{P,C}$ = 3.1 Hz, *N-^{i}Pr,* N-CH_2-((CH_3)$_2$)$_2$), 30.8 (d, $J_{P,C}$ = 2.2 Hz, NCH_2CH_2*CH$_2$*Me), 31 (s, $J_{P,C}$ = 3.2 Hz, NCH_2*CH$_2$*CH_2Me), 46 &46.1 (s, N*CH$_2$*CH_2CH_2Me), 46.4 (d, $J_{P,C}$ = 10.2 Hz, *N-^{i}Pr,* N-*CH$_2$*-Me_2), 126.8 (d, $J_{P,C}$ = 10.3 Hz, *P-C* of the middle ring), 168.1 (br, *C=S*). ^{31}P{^{1}H}-NMR (121.5 Hz, C_6D_6) : δ = -16.8 (d, $^3J_{P,P}$ = 9.1 Hz, *P*- N-^{i}Pr) & -75.9 (d, $^3J_{P,P}$ = 9.1 Hz, *P*-Me) and -19.6 (d, $^3J_{P,P}$ = 11.1 Hz, *P*- N-^{i}Pr) & -69.4 (d, $^3J_{P,P}$ = 11.1 Hz, *P*-Me) *cis* & *trans* isomers (1:3.1). EI-MS (70 eV): m/z (%) = 497.1 (100), $[C_{23}H_{39}N_4P_2S_2]^+$, 597.2 (22), $[M]^+$. HRMS: for $C_{27}H_{48}N_4OP_2S_2$ theor./exp. 597.3217/597.3216. EA: exp. C 45.83, H 7.81, N 8.16, S 8.73; Calc. C 58.26, H 8.93, N 11.71, S 10.72.

3c

Yield : 83.3 mg (0.146 mmol, 71.2 %), white powder. M.p. 118 °C. ^{1}H NMR(300 MHz, C_6D_6) : δ = 0.79-0.86 (m, 12H, $NCH_2CH_2CH_2$*Me*), 1.0 (s, 9H, *O-^{t}Bu*), 1.20-1.35 (m, 8H, NCH_2CH_2*CH$_2$*Me), 1.46 (d, 3H, $^3J_{H,H}$ = 4.9 Hz, *P-Me*), 1.62-1.72 (m, 2H, NCH_2*CH$_2$*CH_2Me), 1.81-1.89 (m, 2H, NCH_2*CH$_2$*CH_2Me), 1.89-1.97 s(m, 4H, NCH_2*CH$_2$*CH_2Me), 4.10-4.18 (m, 2H, N*CH$_2$*CH_2CH_2Me), 4.18-4.21 (m, 2H, N*CH$_2$*CH_2CH_2Me), 4.21-4.27 (m, 2H, N*CH$_2$*CH_2CH_2Me), 4.77-4.85 (m, 2H, N*CH$_2$*CH_2CH_2Me). ^{13}C NMR (75 MHz, C_6D_6) : δ = 13.9 & 14 (s, $NCH_2CH_2CH_2$*Me*), 18.2 (dd, $J_{P,C}$ = 11.8 Hz, $J_{P,C}$ = 3.3 Hz, *P-Me*), 20.31 & 20.34 (s, NCH_2CH_2*CH$_2$*Me), 30.3 (d, $J_{P,C}$ = 7.8 Hz, NCH_2*CH$_2$*CH_2Me), 31.6 (d, $J_{P,C}$ = 11.3 Hz, NCH_2*CH$_2$*CH_2Me), 46.7 (t, $^3J_{P,C}$ = 10.2 Hz, N*CH$_2$*CH_2CH_2Me), 77.6 (d, $J_{P,C}$ = 10.9 Hz, *O-C*(CH_3)$_3$), 132.3 (d, $J_{P,C}$ = 5.25 Hz, *P-C* of the middle ring), 133.2 (d, $J_{P,C}$ = 4.0 Hz, *P-C* of the middle ring), 167.2 (br, *C=S*). ^{31}P{^{1}H}-NMR (121.5 MHz, C_6D_6) : δ = 25.6 (d, $^3J_{P,P}$ = 7.2 Hz, *P*-O-^{t}Bu) & -79.6 (d, $^3J_{P,P}$ = 7.2 Hz, *P*-Me) and 26.5 (d, $^3J_{P,P}$ = 13.4 Hz, *P*-O-^{t}Bu) & -69.4 (d, $^3J_{P,P}$ = 13.4 Hz, *P*-Me) *cis* & *trans* isomers (1:4.1).EI-MS (70 eV): m/z (%) = 514.1 (100), $[C_{23}H_{40}N_4OP_2S_2]^+$, 570.2 (82), $[M]^+$. HRMS: for $C_{27}H_{48}N_4OP_2S_2$ theor./exp. 570.2744/570.2745. IR : ṽ (cm^{-1}) = 2960 (*w*), 2934 (*w*), 2870 (*w*), 1434 (*w*), 1402 (*s*), 1363(*m*), 1253 (*m*), 1217 (*m*), 1160 (*m*), 903 (*s*), 860 (*m*), 800 (*m*). EA : exp. C 56.56, H 8.50, N 9.24, S 10.76; Calc. C 56.81, H 8.47, N 9.81, S 11.23.

P-P coupled product **4a**

1,4-Diphosphinine **1** (100 mg, 0.206 mmol) was taken in a Schlenk tube and KHMDS (42 mg, 0.206 mmol) was added into it followed by the addition of 10 mL dry Et_2O. A rapid colour change was observed from red to bluish violet. Then, the reaction mixture was stirred for an hour at room temperature. The reaction mixture was brought to -90 °C by keeping it in Dewar bath. I_2 (25.5 mg, 0.103 mmol) was dissolved in 2.5 mL diethyl ether and added drop wise into the reaction mixture. A transient deep green colouration was formed which gradually turned to reddish brown. After stirring for 1 hour, the reaction mixture turned bright orange and the solvent was removed *in vacuo*. The residue was re-dissolved in n-pentane and filtered out via a cannula to remove the potassium iodide salt formed in course of the reaction. Solvent was removed *in vacuo* to obtain a bright orange powder **4a**.

Yield : 111 mg (0.0863 mmol, 42 %), orange powder. M.p. 165 °C. ^{1}H NMR(300MHz, $CDCl_3$) : δ = -0.32 (s, 18H, $N(Si(CH_3)_3)_2$), 0.44 (d, 18H, $^{4}J_{P,H}$ = 2.1 Hz, $N(Si(CH_3)_3)_2$), 0.94 (d, 12H, $^{3}J_{H,H}$ = 21 Hz, $NCH_2CH_2CH_2$*Me*), 1.1 (t, 12H, $^{3}J_{H,H}$ = 21 Hz, $NCH_2CH_2CH_2$*Me*), 1.29-1.40 (m, 8H, NCH_2CH_2*CH_2*Me), 1.44-1.55 (m, 8H, NCH_2CH_2*CH_2*Me), 1.7-1.86 (m, 8H, NCH_2*CH_2*CH_2Me), 1.9-2.1 (m, 8H, NCH_2*CH_2*CH_2Me), 3.01-3.17 (m, 4H, N*CH_2*CH_2CH_2Me), 3.83-4.01 (m, 4H, N*CH_2*CH_2CH_2Me), 4.34-4.55 (m, 8H, N*CH_2*CH_2CH_2Me). ^{13}C NMR (75MHz, $CDCl_3$) : δ = 3.4 (s, *N-SiMe$_3$*), 4.7 (d, $^{2}J_{P,C}$ = 15.7 Hz, *N-SiMe$_3$*), 13.8 & 13.9 (s, $NCH_2CH_2CH_2$*Me*), 20.2 & 20.5 (s, NCH_2CH_2*CH_2*Me), 30.6 & 30.7 (s, NCH_2*CH_2*CH_2Me), 47.3 (t, $^{3}J_{P,C}$ = 4.8 Hz, N*CH_2*CH_2CH_2Me), 47.7 (br. t, N*CH_2*CH_2CH_2Me), 134.8 (br. s, *P-C* of the middle ring), 135.2 (br.s, *P-C* of the middle ring), 167.7 (s, *C=S*). $^{31}P\{^{1}H\}$-NMR (121.5 MHz, $CDCl_3$) : δ = -0.34 (t, $^{3}J_{P,P}$ = 25.6 Hz, *P*-$N(TMS)_2$), -50.9 (d, $^{3}J_{P,P}$ = 25.6 Hz, P-P). IR : ṽ (cm^{-1}) = 2956 (*w*), 2930 (*w*), 2861 (*w*), 1436 (*m*), 1397 (*s*), 1257 (*m*), 1216 (*m*), 796 (*w*), 680 (*w*). UV/Vis (Et_2O): λ_{max} in nm (ε in $Lmol^{-1}cm^{-1}$) = 440 (ε = 9210). EA : exp. C 51.14, H 8.43, N 9.75, S 9.30; Calc. C 52.30, H 8.46, N 10.89, S 9.97.

2. Spectra for compound K[**2a**]

Figure S1 : ^{1}H NMR spectrum of K[**2a**] in Et_2O-*d*10 (300 MHz, 25 °C)

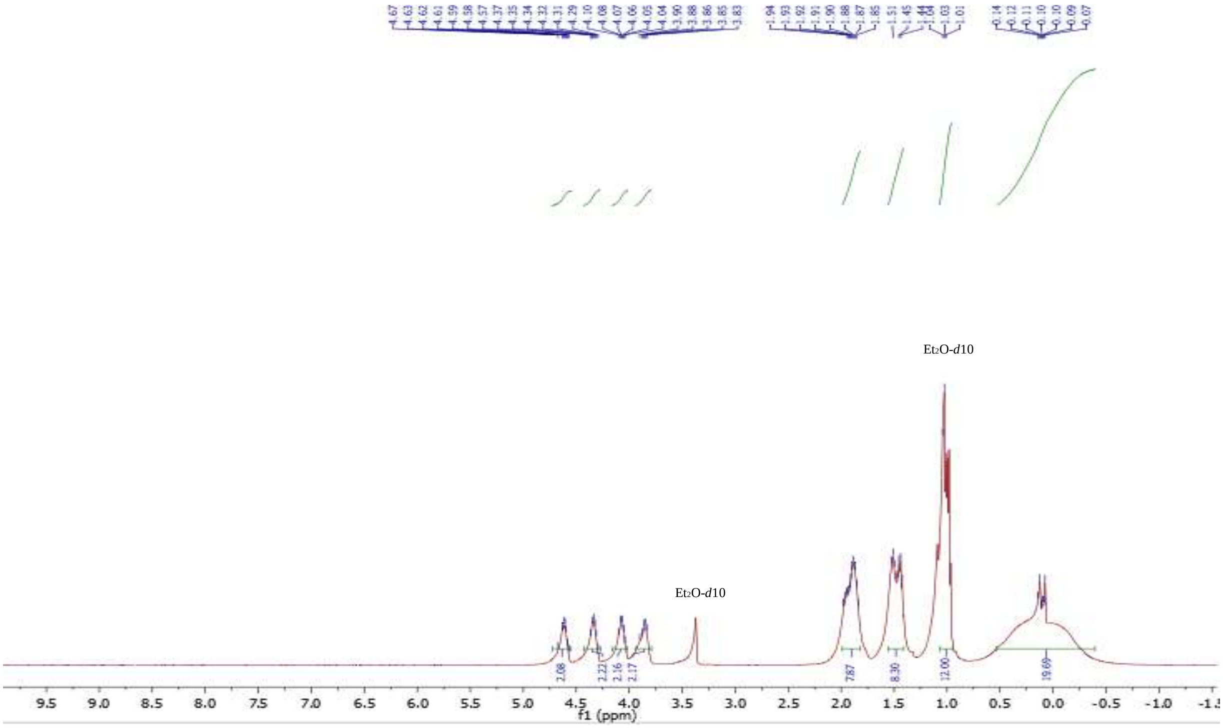

Figure S2 : ^{13}C NMR spectrum of K[**2a**] in Et_2O-*d*10 (75 MHz, 25 °C)

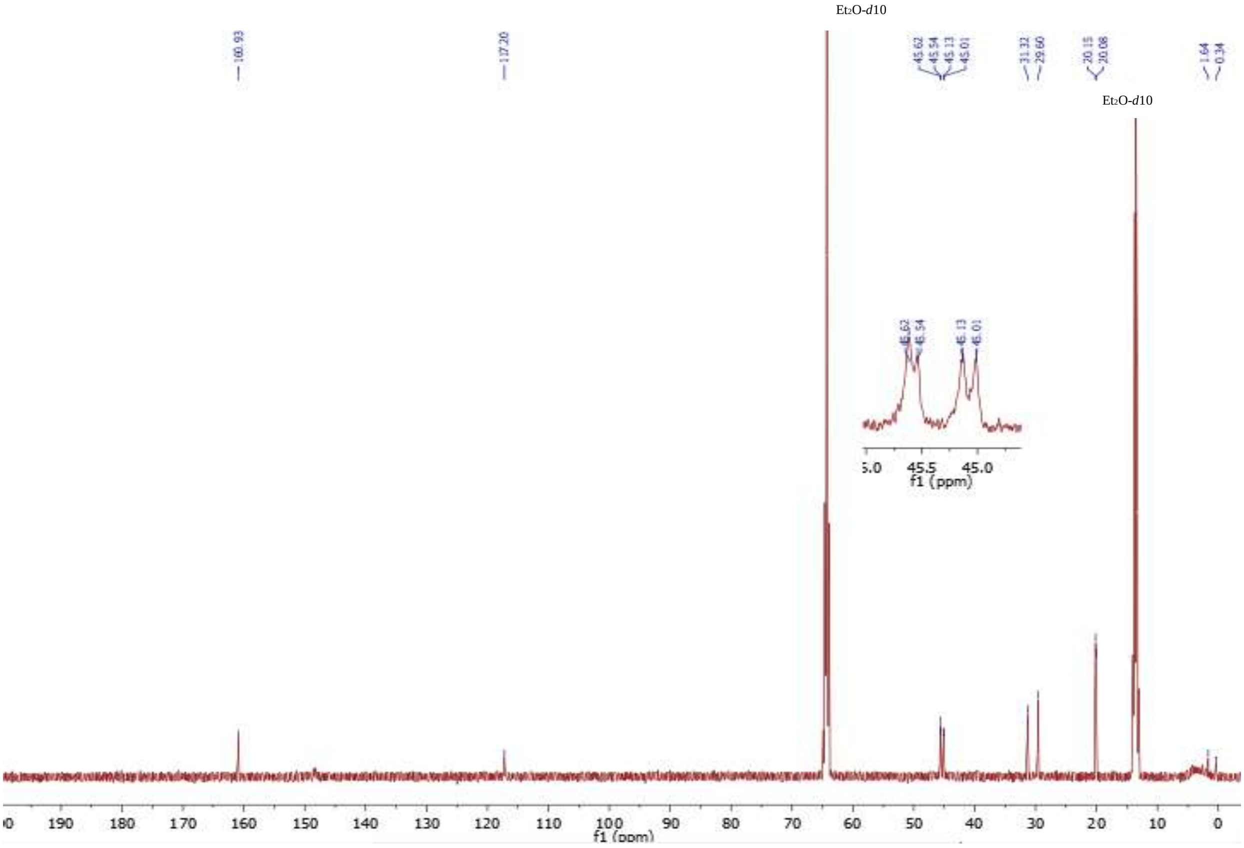

Figure S3 : ^{31}P NMR spectrum of K[**2a**] in Et_2O-*d*10 (121.5 MHz, 25 °C)

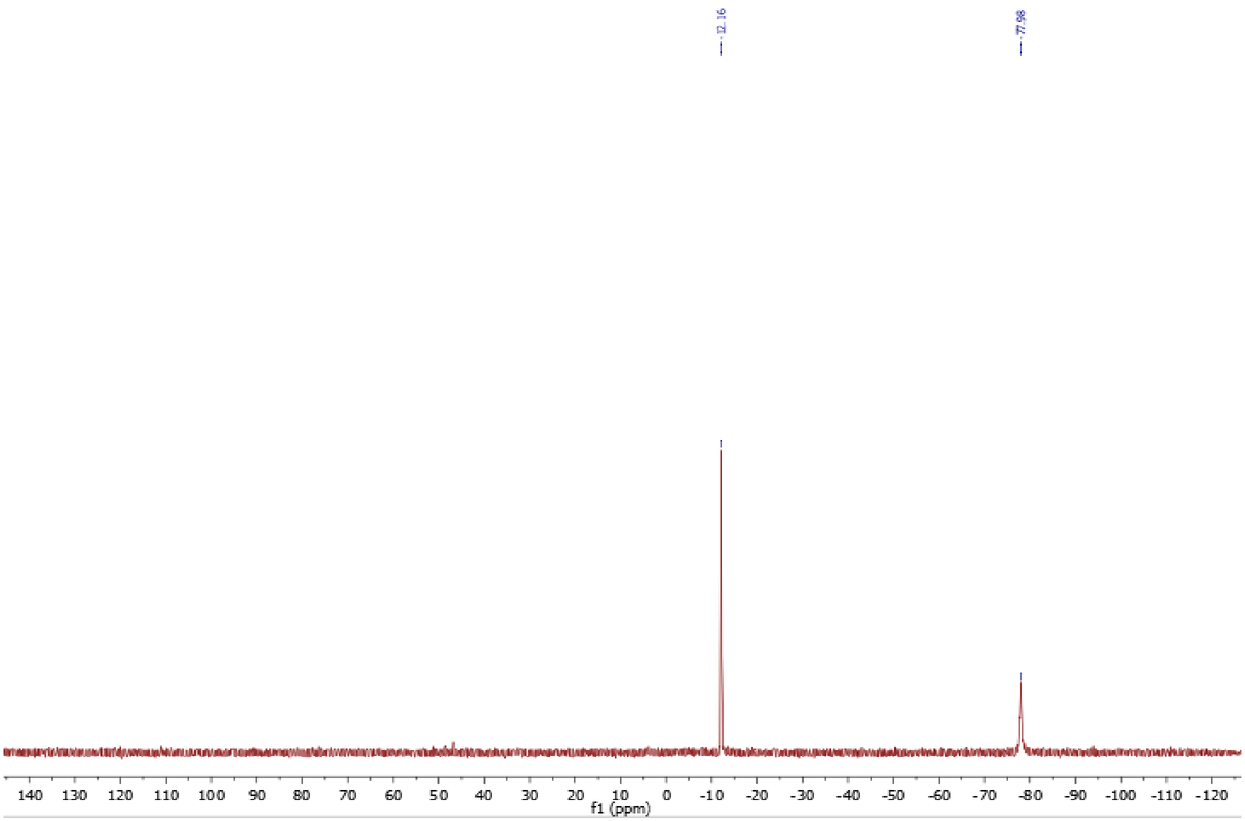

Figure S4 : neg-ESI-MS spectrum of K[**2a**]

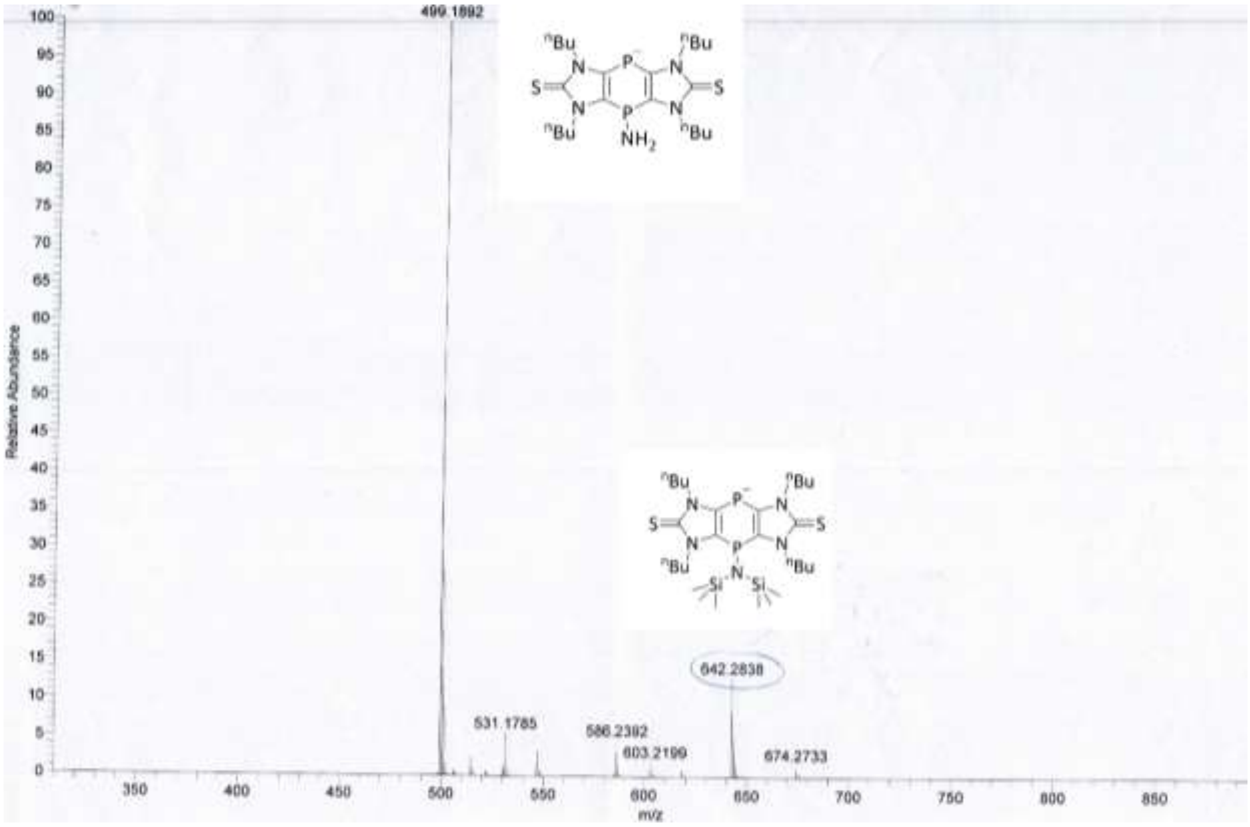

Figure S5 : UV-Vis spectrum of K[**2a**] in (a) Et_2O and (b) CH_3CN

(a)

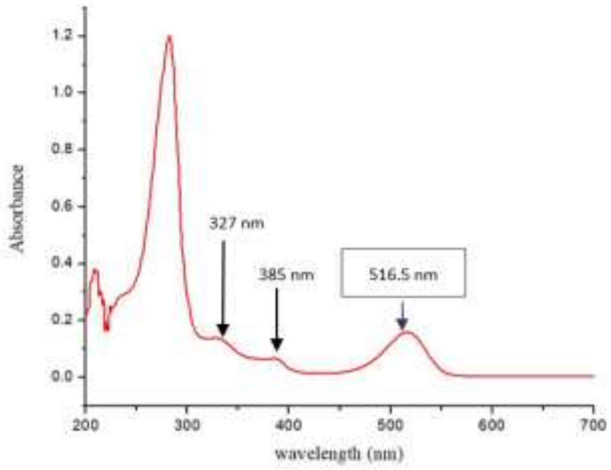

(b)

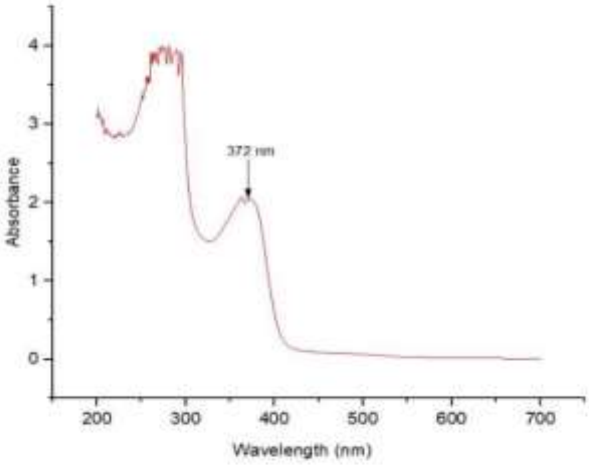

3. Spectra for compound Li[**2b**]

Figure S6 : ^{1}H NMR spectrum of Li[**2b**] in Et_2O-*d*10 (300 MHz, 25 °C)

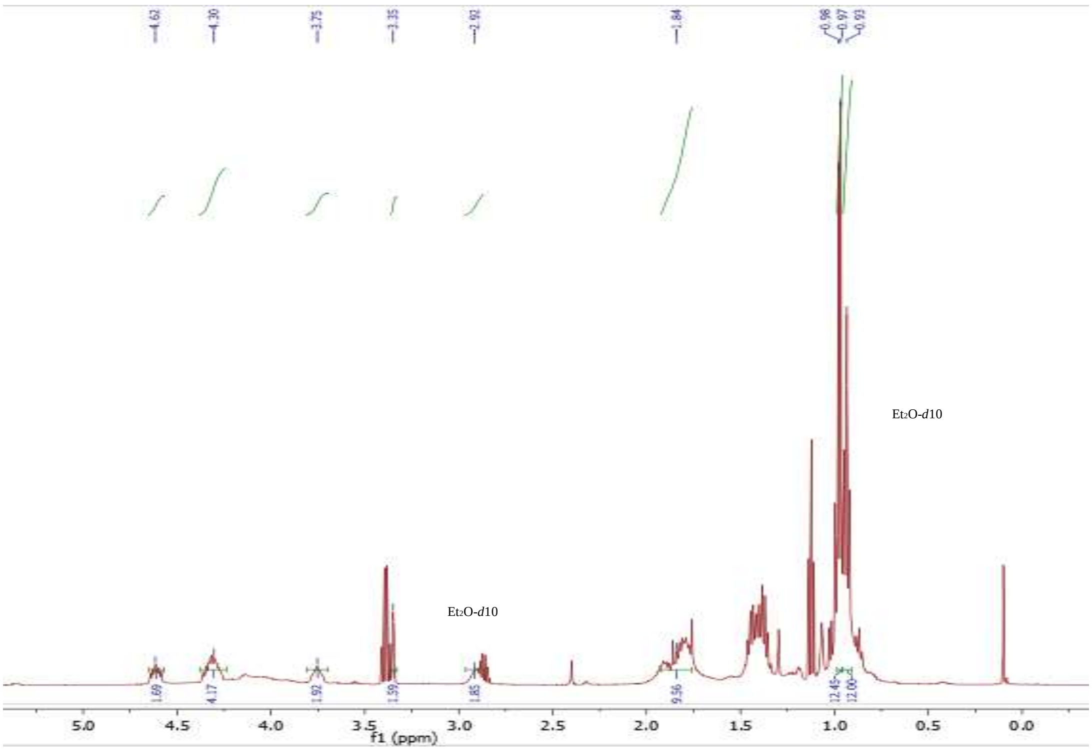

Figure S7 : ^{13}C NMR spectrum of Li[**2b**] in Et_2O-*d*10 (75 MHz, 25 °C)

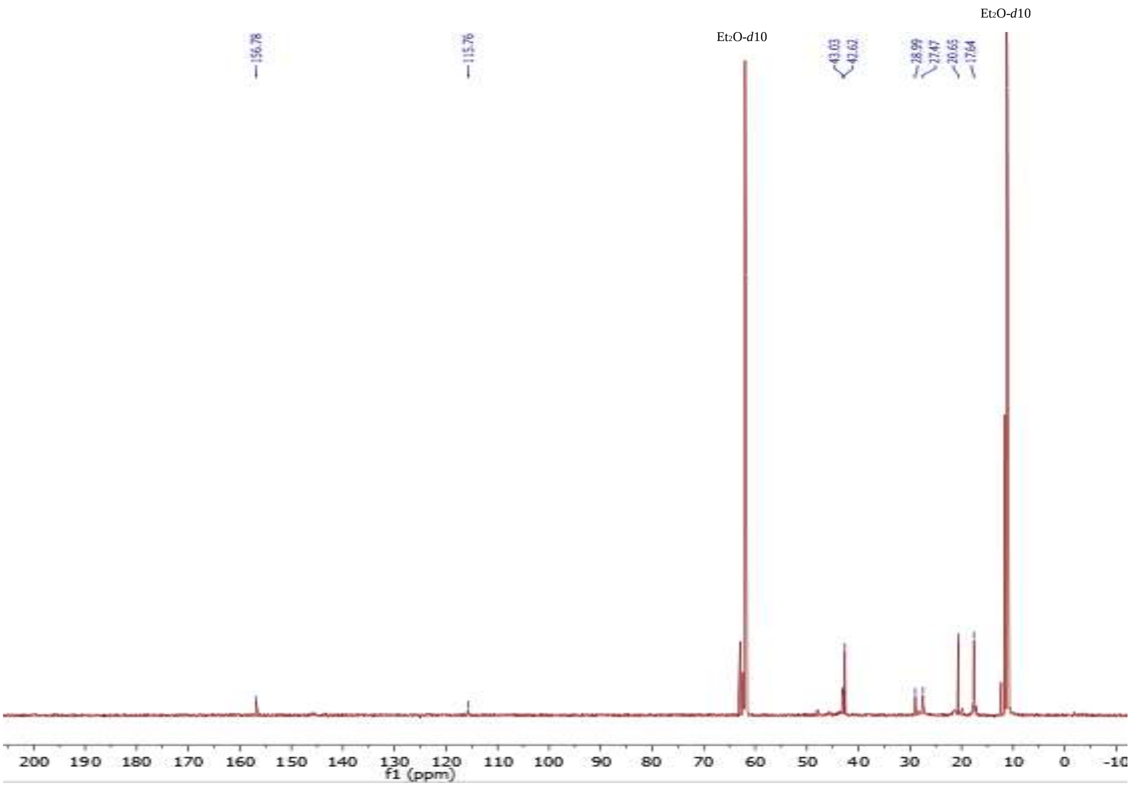

Figure S8 : ^{31}P NMR spectrum of Li[**2b**] in Et_2O-*d*10 (121.5 MHz, 25 °C)

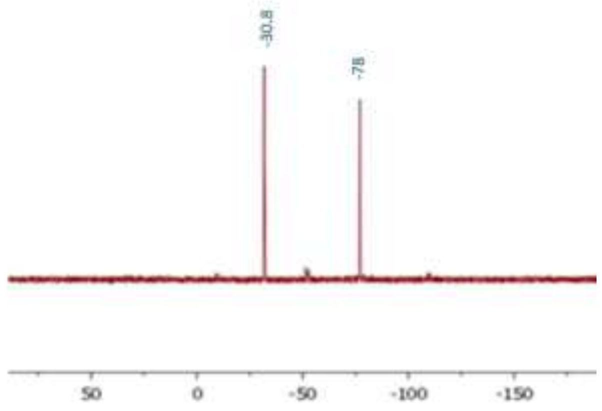

Figure S9 : UV-Vis spectrum of Li[**2b**] in (a) Et_2O (b) CH_3CN.

(a)

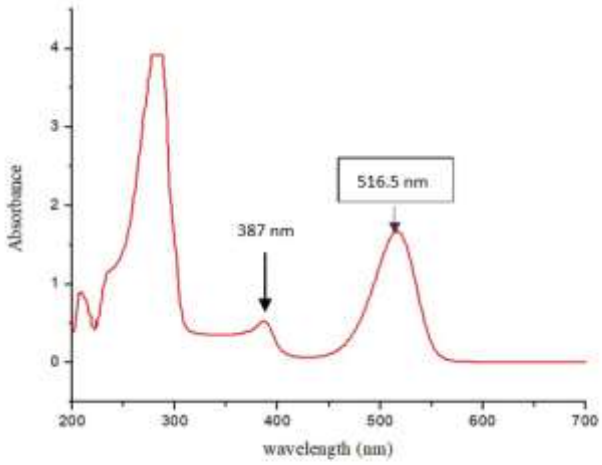

(b)

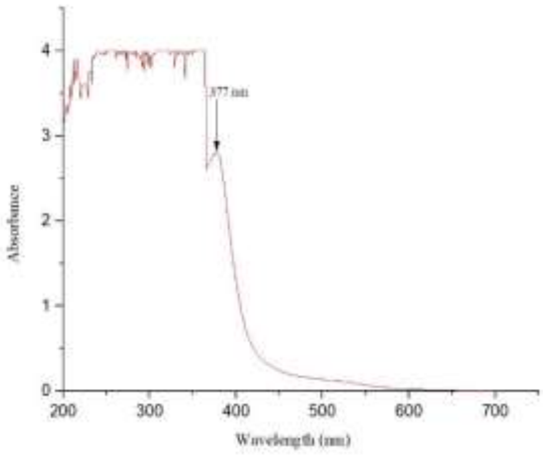

4. Spectra for compound K[**2c**]

Figure S10 : ^{1}H NMR spectrum of K[**2c**] in Et_2O-*d*10 (300 MHz, 25 °C)

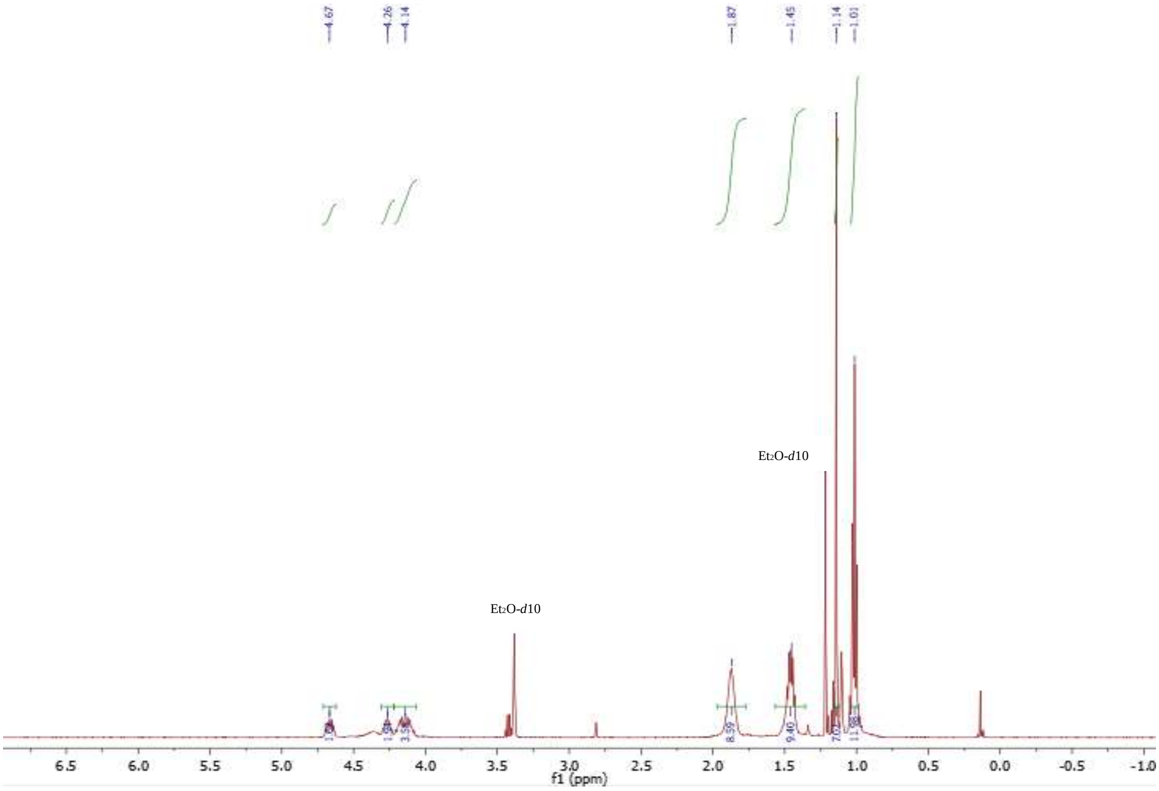

Figure S11 : ^{13}C NMR spectrum of K[**2c**] in Et_2O-*d*10 (75 MHz, 25 °C)

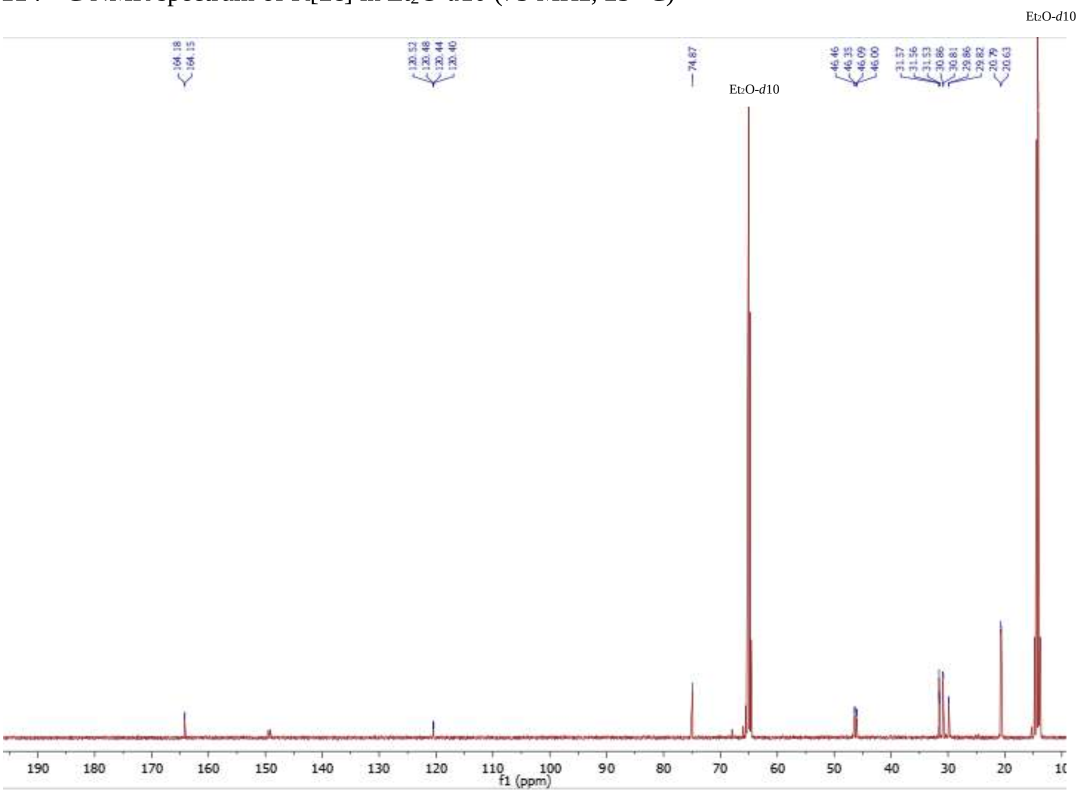

Figure S12 : ^{31}P NMR spectrum of K[**2c**] in Et_2O-*d*10 (121.5 MHz, 25 °C)

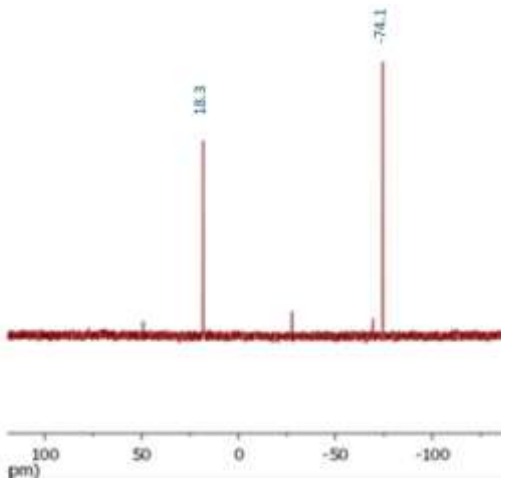

Figure S13 : UV-Vis spectrum of K[**2c**] in (a) Et_2O and (b) CH_3CN

(a)

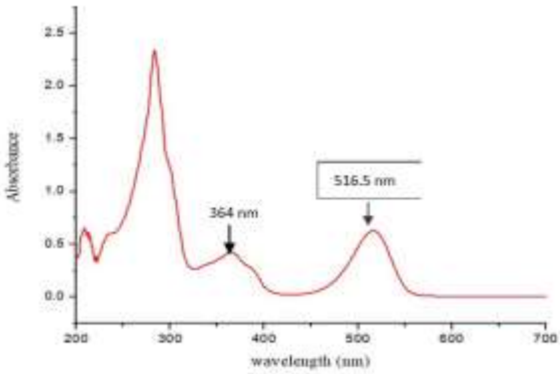

(b)

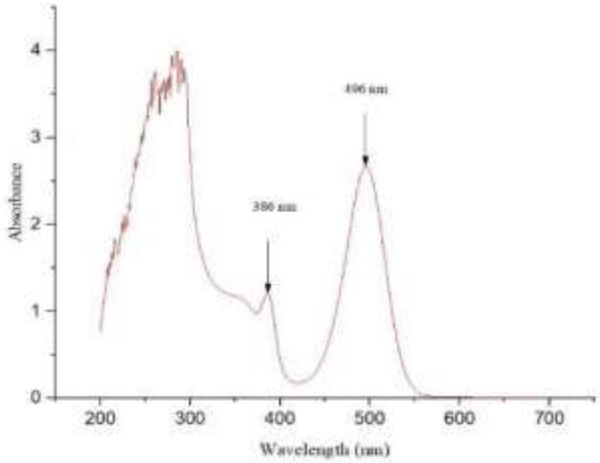

5. Spectra for compound **3a**

Figure S14 : ^{1}H NMR spectrum of **3a** in C_6D_6 (300 MHz, 25 °C)

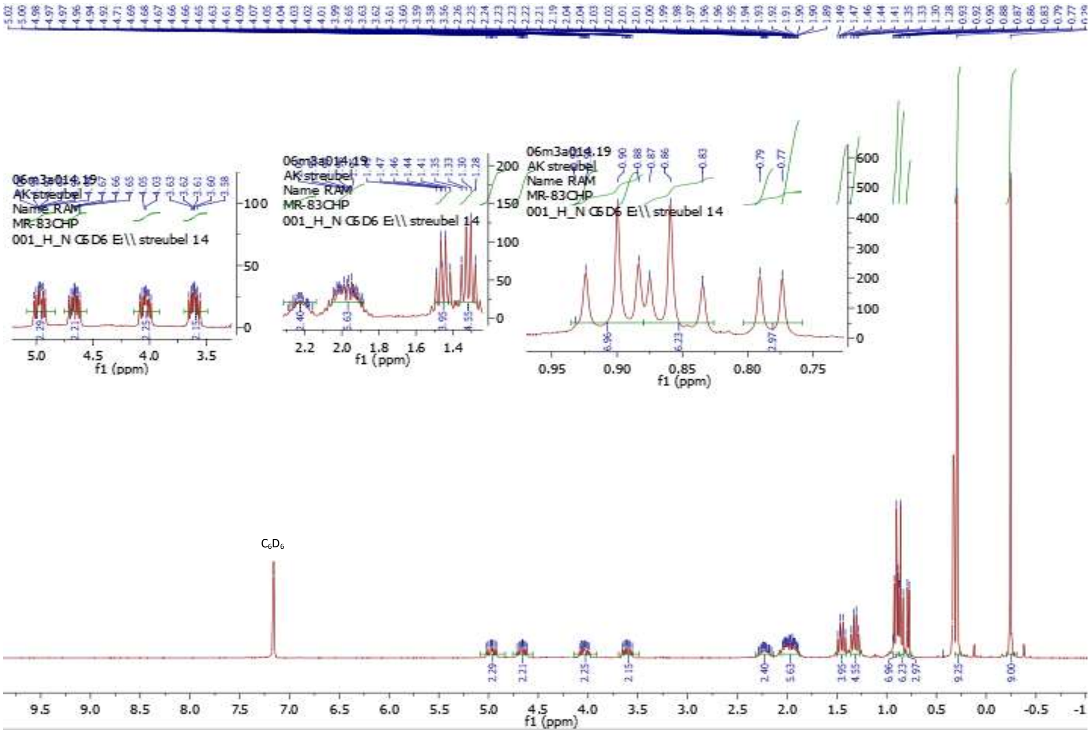

Figure S15 : ^{13}C NMR spectrum of **3a** in C_6D_6 (75 MHz, 25 °C)

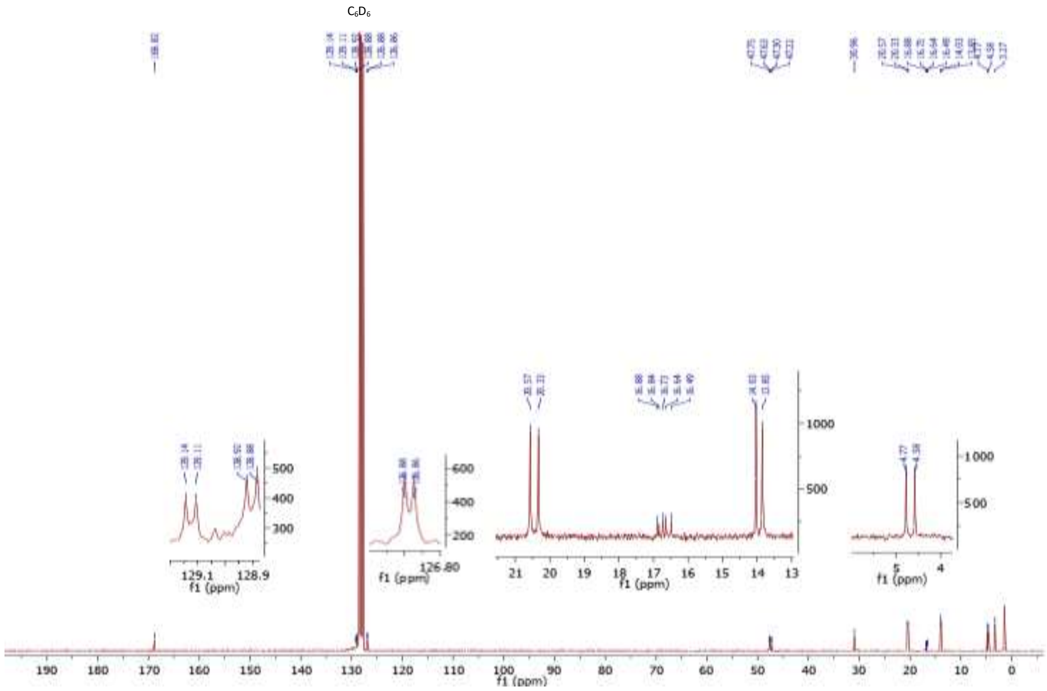

Figure S16 : ^{31}P NMR spectrum of **3a** in C_6D_6 (121.5 MHz, 25 °C)

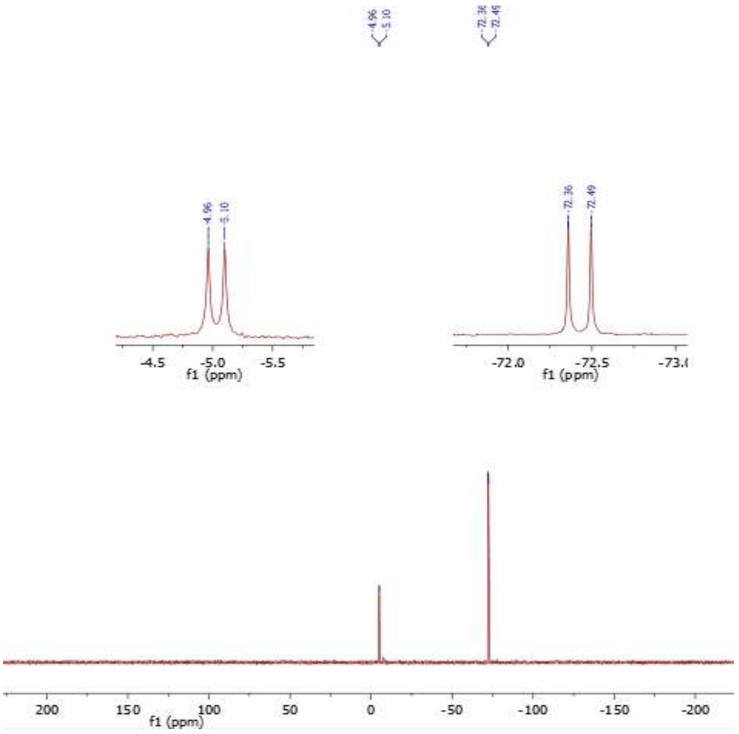

Figure S17 : EI-MS spectrum of **3a**

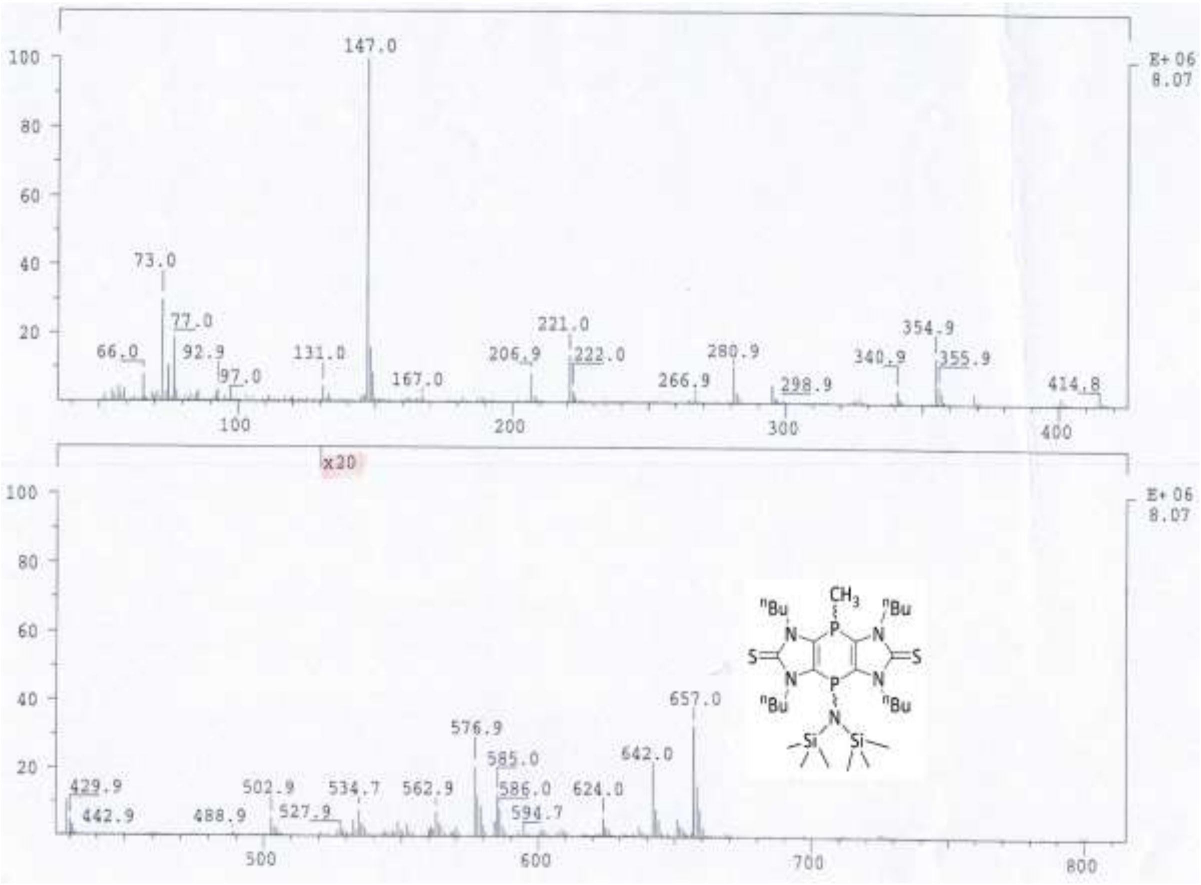

6. Spectra for compound **3b**

Figure S18 : ^{1}H NMR spectrum of **3b** in C_6D_6 (300 MHz, 25 °C)

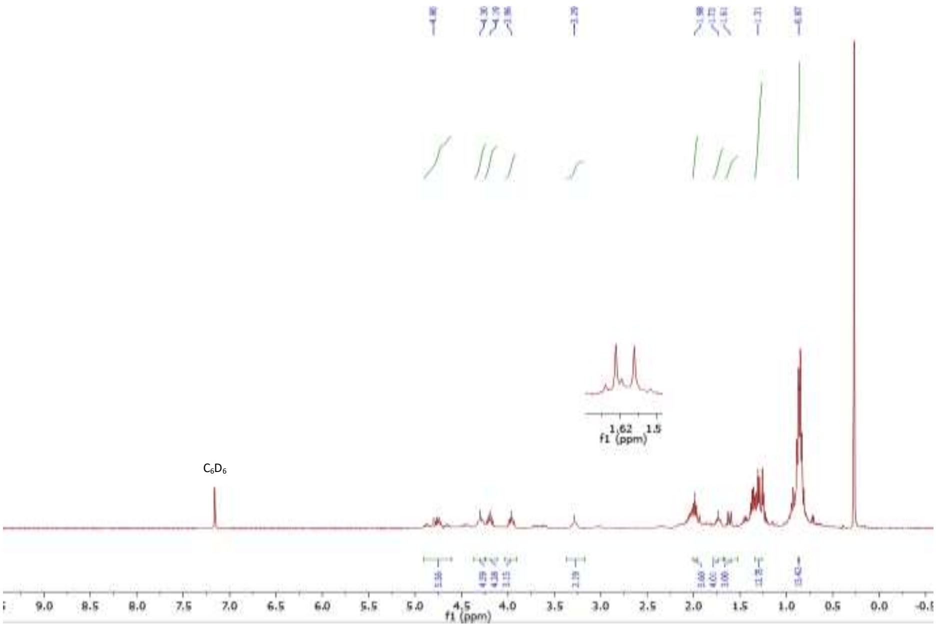

Figure S19 : ^{13}C NMR spectrum of **3b** in C_6D_6 (75 MHz, 25 °C)

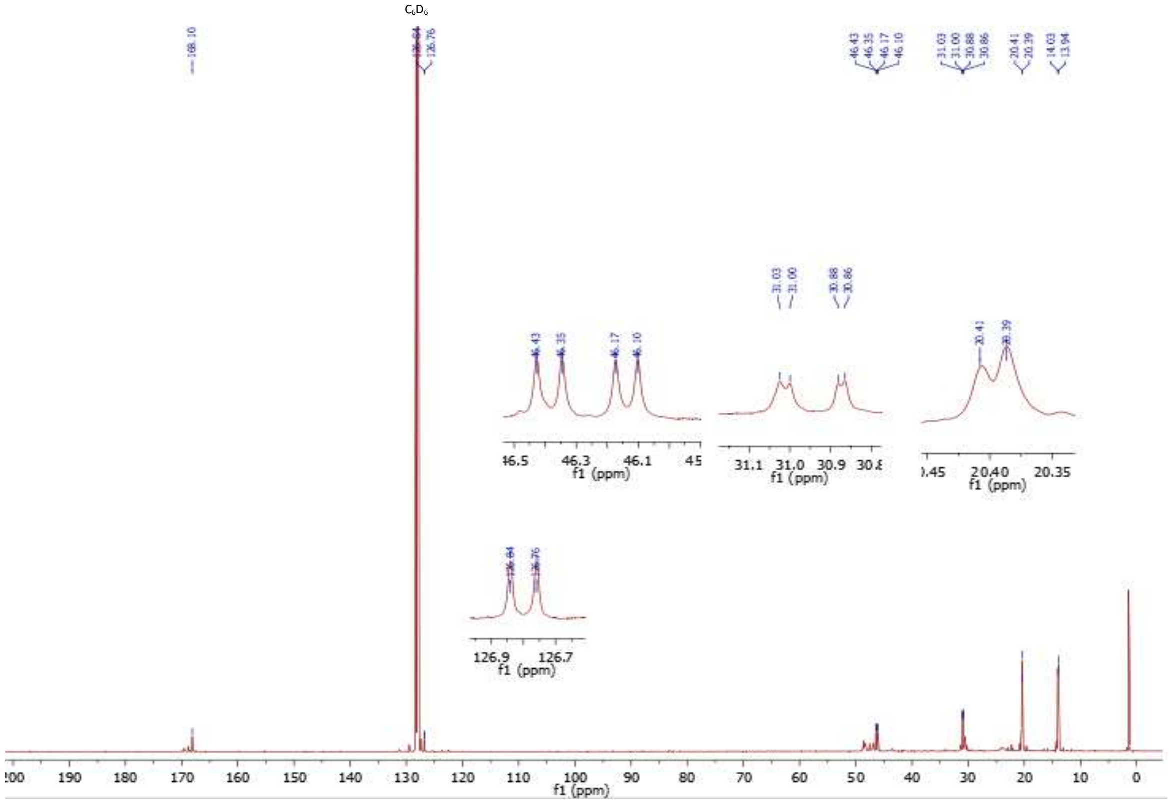

Figure S20 : ^{31}P NMR spectrum of **3b** in C_6D_6 (121.5 MHz, 25 °C)

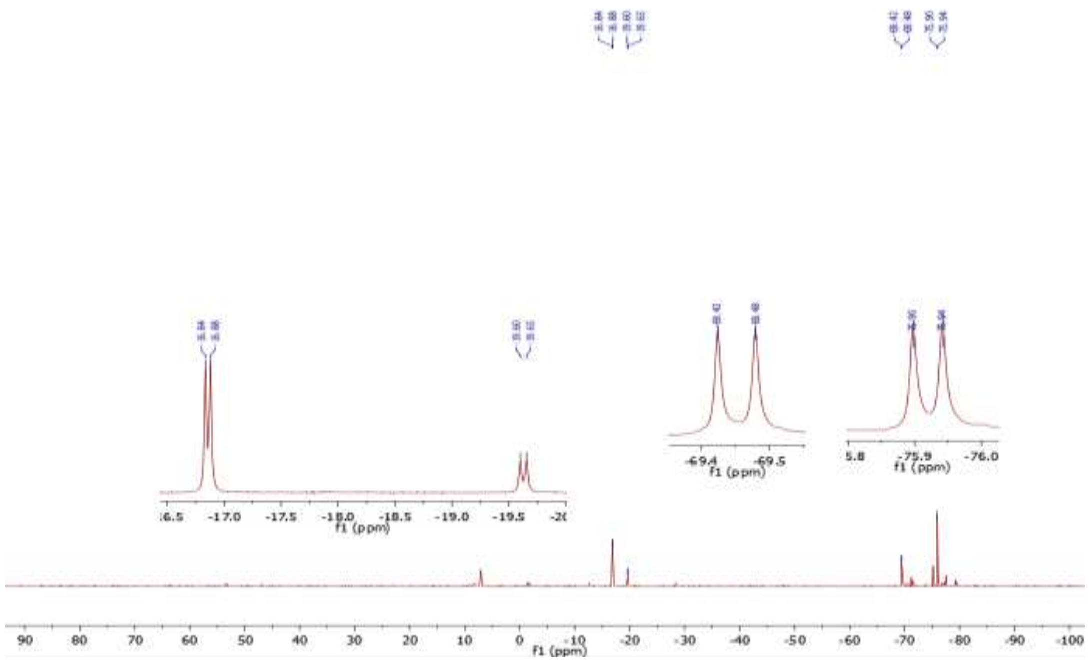

Figure S21 : EI-MS spectrum of **3b**

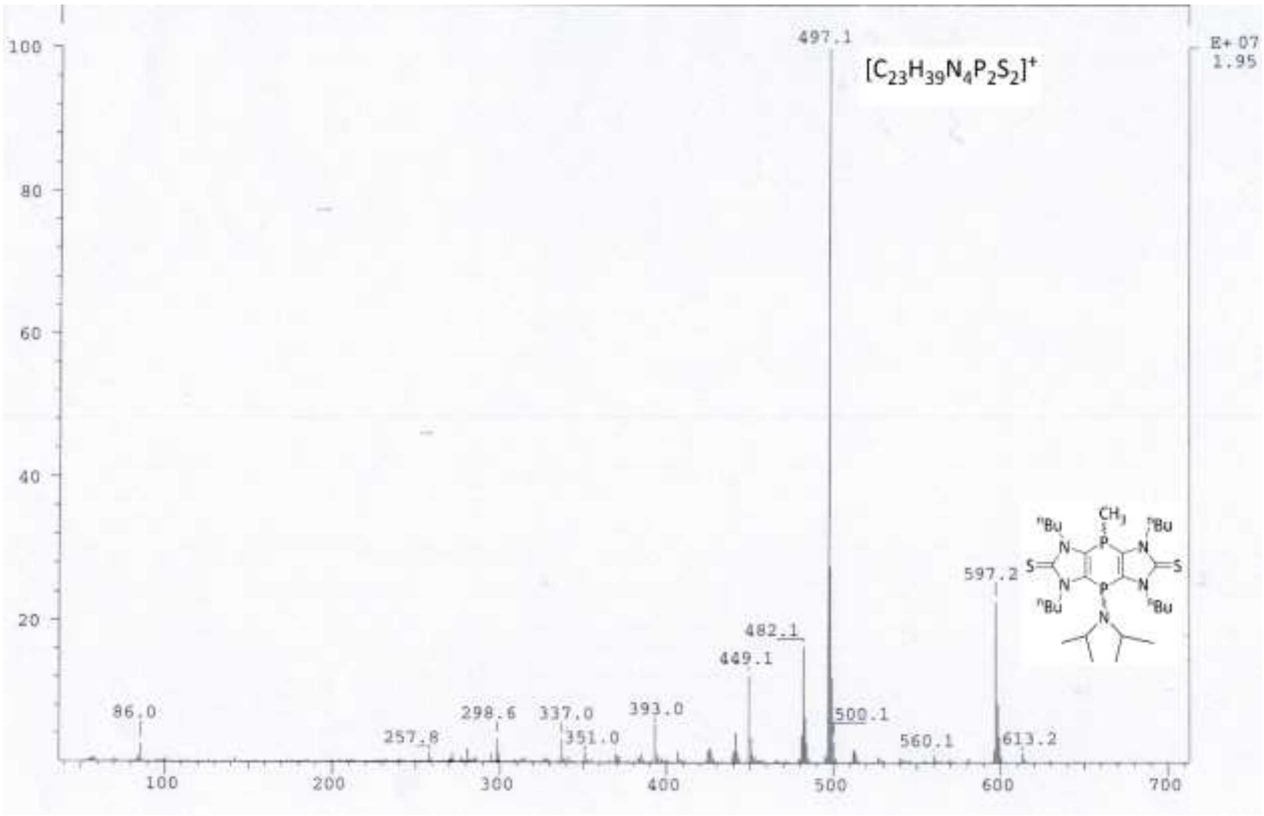

7. Spectra for compound **3c**

Figure S22 : ^{1}H NMR spectrum of **3c** in C_6D_6 (300 MHz, 25 °C)

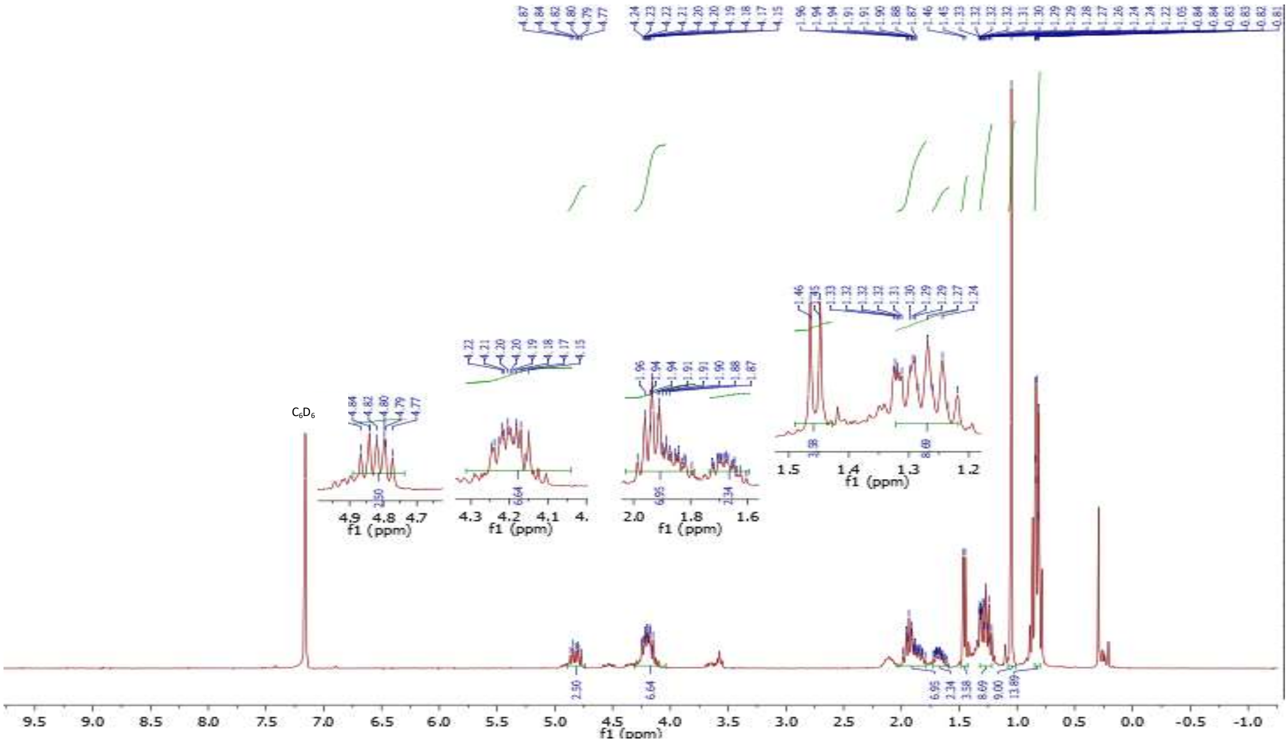

Figure S23 : ^{31}P NMR spectrum of **3c** in C_6D_6 (121.5 MHz, 25 °C)

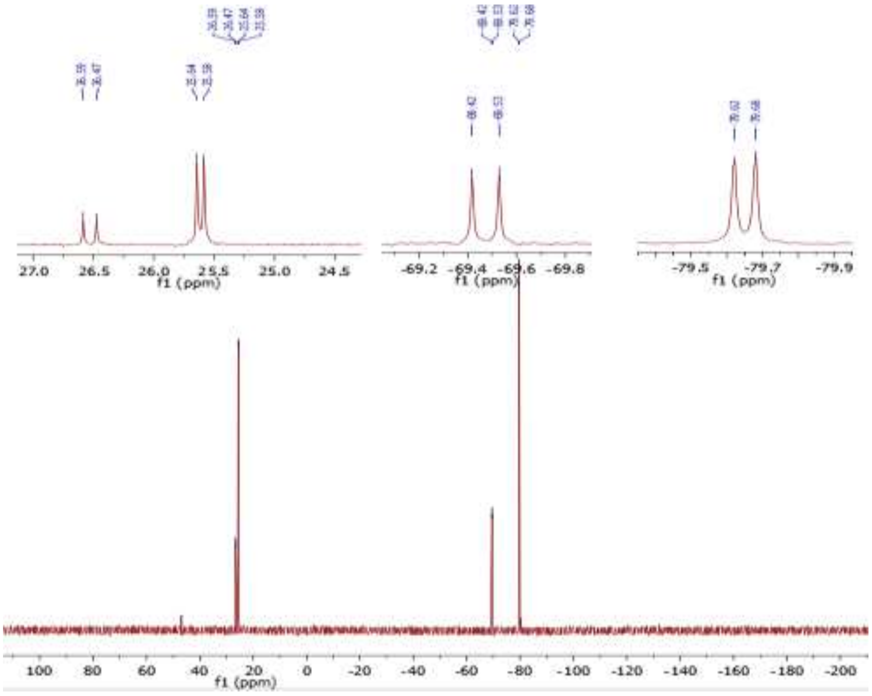

Figure S24 : EI-MS spectrum of **3c**

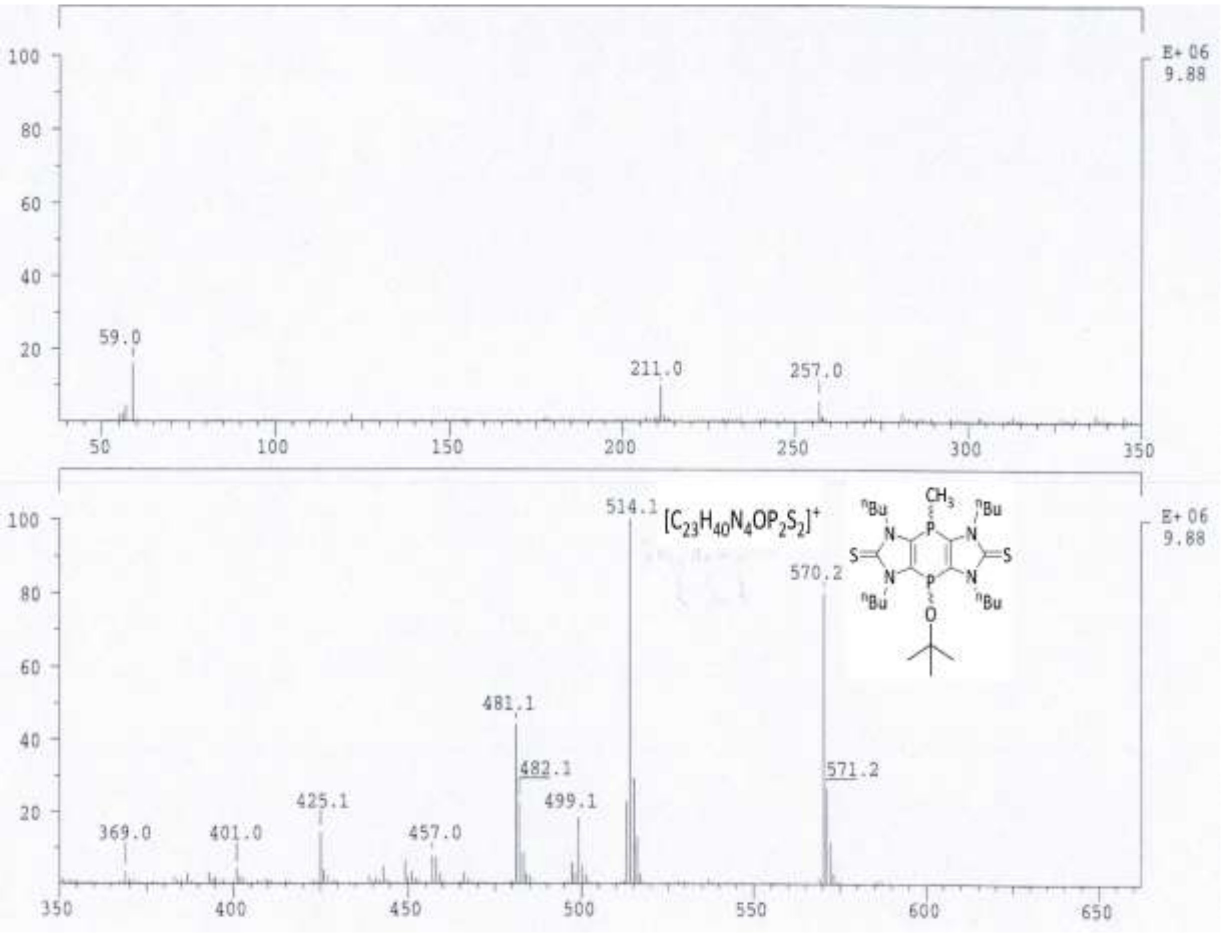

8. Spectra for compound **4a**

Figure S25 : ^{1}H NMR spectrum of **4a** in $CDCl_3$ (300 MHz, 25 °C)

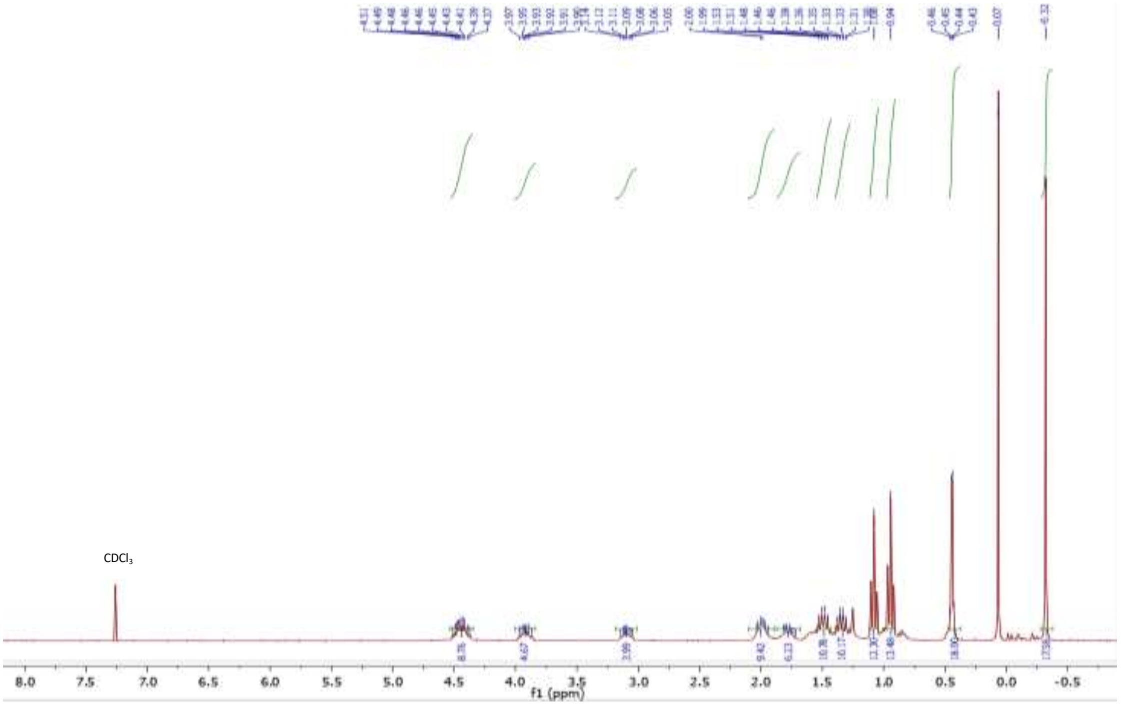

Figure S26 : ^{13}C NMR spectrum of **4a** in $CDCl_3$ (75 MHz, 25 °C)

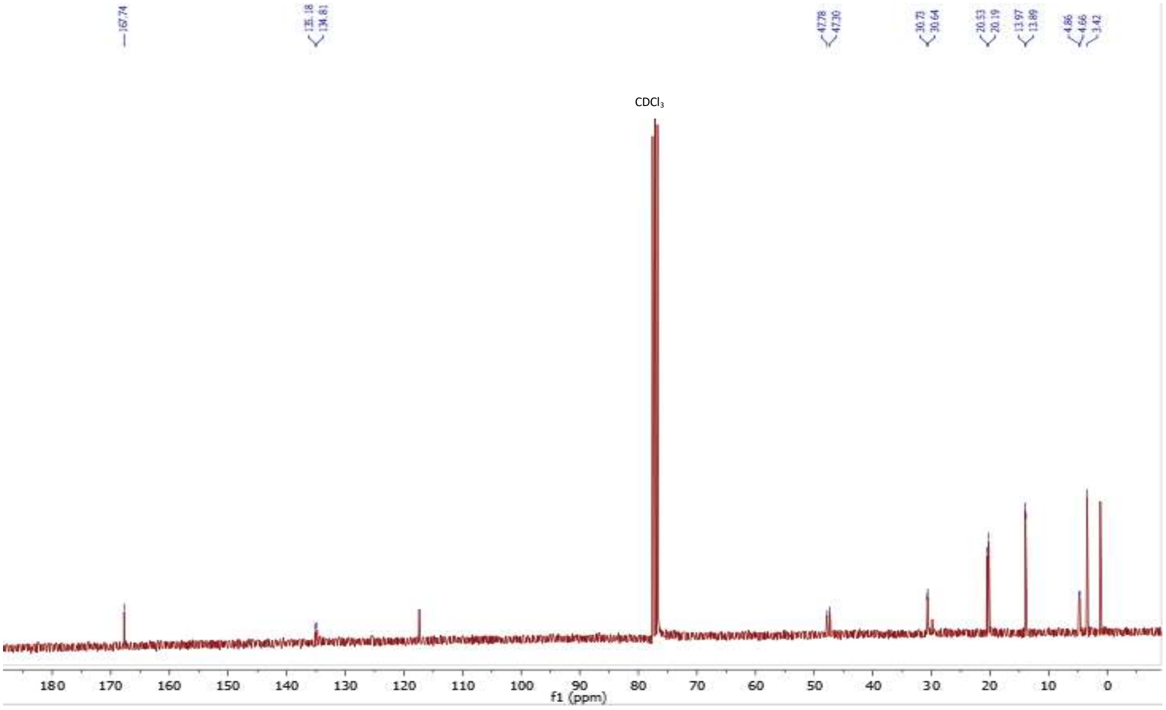

Figure S27 : ^{31}P NMR spectrum of **4a** in $CDCl_3$ (121.5 MHz, 25 °C)

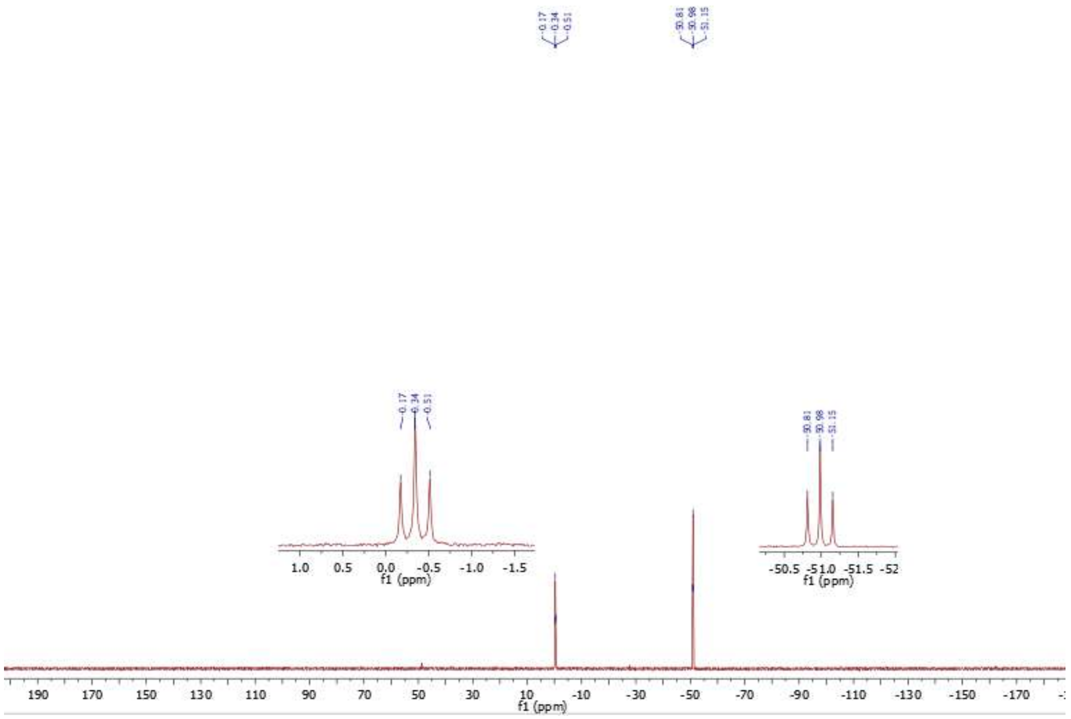

Figure S28 : EI-MS spectrum of **4a**

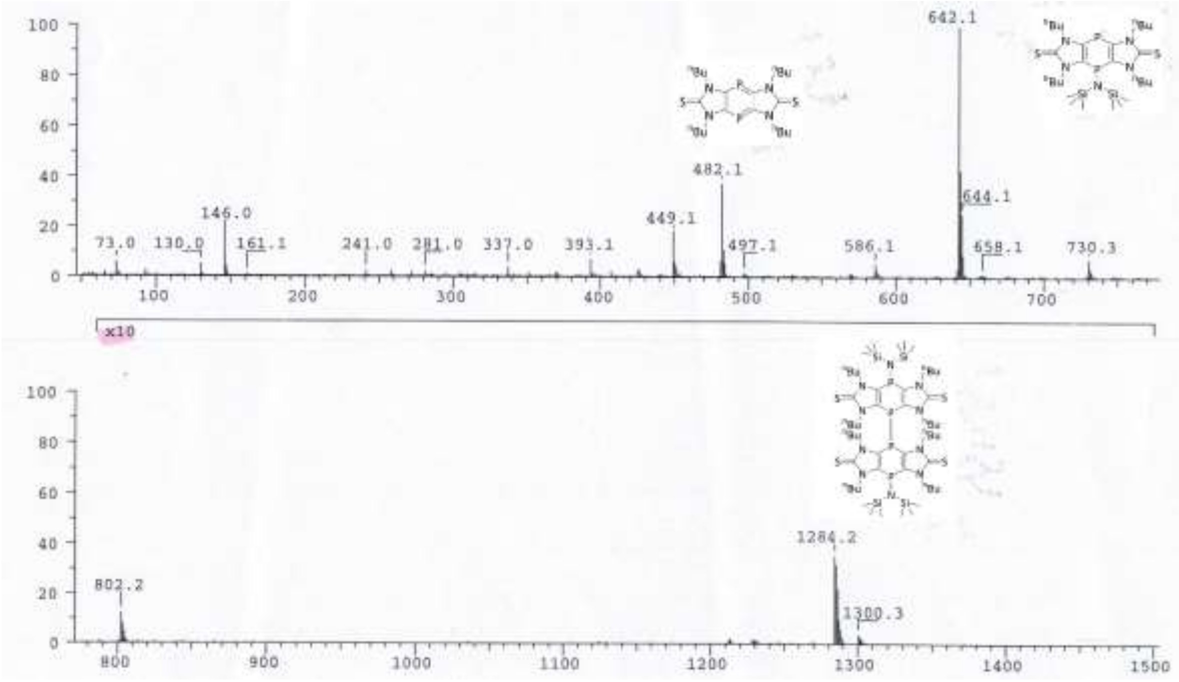

Table S1 : Selected neg. ESI-MS spectrometric data of the anions of M[**2a-c**]**.**

**Compound**	**m/z (%)**	**Ion composition**
K[**2a**]	642.2 (22)	$[M]^+$
Li[**2b**]*	614.287 (90)	$[M+O_2]^+$
K[**2c**]*	499.189 (100)	$[C_{22}H_{37}N_4OP_2S_2]^+$

* Li[**2b**] and K[**2c**] had stability issues while measuring ESI-MS. However, the oxidation product obtained for Li[**2b**], and the fragment obtained for K[**2c**] is also in accordance with the presence of the respective anion.

9. X-ray diffraction studies

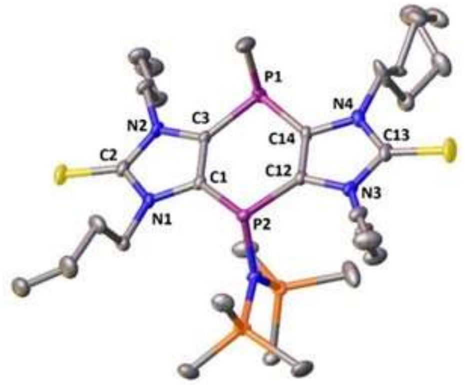

Figure S29 : Molecular structure of *trans*-**3a**; hydrogen atoms are omitted for clarity (50 % probability level). Selected bond lengths [Å] and angles [º]: P1-N5 1.7161(16), P2-C29 1.846(3), P1-C1 1.8240(19), P1-C12 1.816(2), P2-C3 1.803(2), P2-C14 1.8033(19); C1-P1-C12 95.19(9), C3-P2-C14 96.11(9), Σ<°P1 307.86 and Σ<°P2 297.08.

Crystal Data for **3a** : Suitable single-crystals of 3a were obtained from a concentrated diethyl ether solution at 25 °C. Data were collected with a with a Bruker X8-Kappa Apex II diffractometer equipped with a low-temperature device at 100 K by using graphite monochromated Mo Kα radiation (λ = 0.71073 Å). $C_{29}H_{57}N_5Si_2P_2S_2$, M = 658.03, crystal dimensions 0.22 × 0.21 × 0.16 mm^3, triclinic, space group P -1, Z = 2, a = 9.9567(4) Å, b = 10.2653(3) Å, c = 19.9184(7) Å, α = 100.491(2)°, β = 91.546(2)°, γ = 107.701(2)°, V = 1899.64(12) $Å^3$, ρ_{calc} = 1.150 g cm^{-3}, μ = 0.313 mm^{-1}, T = 100 K, transmission factors (min./max.) 0.6717/0.7461, empirical absorption correction, $2\theta_{max}$= 61.014°, no. of unique data 9184 [R_{int} = 0.0490, R_{sigma} = 0.0277], R_1 (for I > 2σ(I)) = 0.0538, wR2 (for all data) = 0.1187, final R = 0.0588, goodness of fit 1.206, ΔF(max./min.) 0.57/-0.30 e $Å^{-3}$.

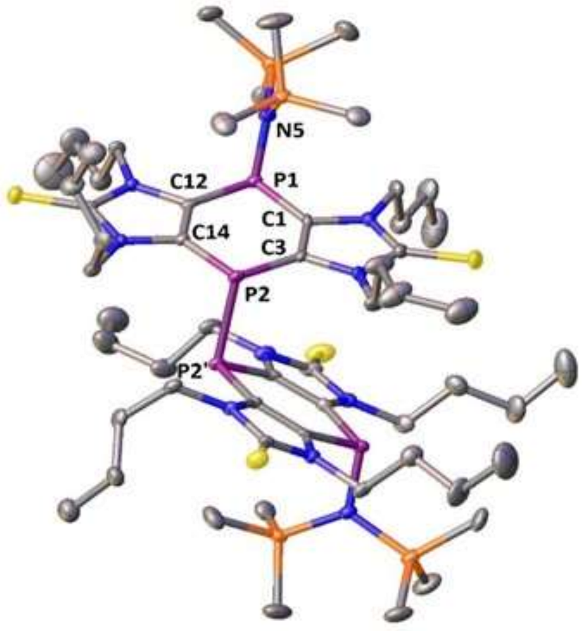

Figure S30 : Molecular structure of compound **4a**; hydrogen atoms are omitted for clarity (50 % probability level). Selected bond lengths [Å] and angles [º]: P1-C1 1.807(4), P1-C12 1.817(4), P2-C3 1.791(4), P2-C14 1.806(4), C12-C14 1.360(5), C1-C3 1.376(5), P1-N5 1.718(3), P2-P2' 2.303(2); C1-P1-C12 94.43(18), C3-P2-C14 96.40(18), Σ<°P1 304.48 and Σ<°P2 300.79.

Crystal Data for **4a** : Suitable single-crystals of 3a were obtained from a concentrated diethyl ether solution at 25 °C. Data were collected with a with a Bruker X8-Kappa Apex II diffractometer equipped with a low-temperature device at 100 K by using graphite monochromated Mo Kα radiation (λ = 0.71073 Å). $C_{56}H_{108}N_{10}P_4S_4Si_4$, M = 1286.00, crystal dimensions 0.18 × 0.12 × 0.1mm^3, monoclinic, space group C2/c, Z = 4, a = 13.0219(16) Å, b = 18.485(2) Å, c = 30.246(4) Å, α = 90°, β = 92.646(4)°, γ = 90°, V = 7273.0(15) $Å^3$, ρ_{calc} = 1.174 g cm^{-3}, μ = 0.325 mm^{-1}, T = 100 K, transmission factors (min./max.) 0.6375/0.7459, empirical absorption correction, $2\theta_{max}$= 60.764°, no. of unique data 8703 [R_{int} = 0.1736, R_{sigma} = 0.1520], R_1 (for I > 2σ(I)) = 0.0886, wR2 (for all data) = 0.1456, final R = 0.1612, goodness of fit 1.058, ΔF(max./min.) 0.58/-0.53 e $Å^{-3}$.

10. Electrochemistry experiments and supplementary results

10.1 Experimental details

The solvent tetrahydrofuran (THF) was first distilled and further purified by recondensation over potassium mirror, and subsequently stored under argon (Ar) in a controlled-atmosphere glove box. Acetonitrile (CH_3CN) for voltammetry was double-distilled, initially, over CaH_2 and then over P_2O_5 and then, degassed. This was subsequently stored under argon (Ar) in a controlled-atmosphere glove box. All solution and sample preparations were undertaken within the same glove box. Solutions for voltammetry were 0.2 M in nBu$_4$NPF$_6$ (purchased from Aldrich and dried for several hours under vacuum before use). After background scans were taken on the solvent/electrolyte solutions, the analytes K[**2a**] or **4a** were added to make a 2.0 mM solution. After all measurements were completed, ferrocene ($C_{10}H_{10}Fe$, hereafter designated as Fc) was added to a concentration of 2.0 mM and served as an internal reference using the ferrocene/ferrocenium ($Fc^{+/0}$) redox couple, set to 0 V according to IUPAC recommendations.[2] Voltammograms were obtained using a Pine Instruments, Inc., WaveNano potentiostat/galvanostat connected to platinum (Pt) or gold (Au) screen-printed electrodes on rugged ceramic substrates. The patterned electrodes formed an inner working disk and outer auxiliary ring separated by an Ag/AgCl spot (for further information, consult the website at https://www.pineresearch.com/shop/products/electrodes/screen-printed-electrodes/ceramic/). Experiments were controlled and data were processed using AfterMath software (https://pineresearch.com/shop/kb/knowledge-category/downloads/).

Background scans established an electrochemical window from -3.2 to +0.06 V (3.8 V wide) in THF/[^{n}Bu$_4$N][PF$_6$] and -3.3 to +2.2 V (5.5 V wide; all potentials mentioned are corrected to $Fc^{+/0}$) and identified the anodic and cathodic limits with respect to the nominal voltage of the solid silver 'dot' reference. Next, open circuit potential measurements were taken to establish the starting point of CV experiments. Careful CV scans were then taken in the anodic and cathodic directions to encounter the most accessible processes, and only after these were investigated thoroughly were scans taken to higher positive and negative potentials. Only at the end of the investigation is the Fc reference compound added to the mixture, and then scans are carefully repeated at the standard scan rate of 0.2 $V{\cdot}s^{-1}$ so as to include the Fc+/0 signal within the scan windows and potentials transferred to the key processes (**I** for K[**2a**] or **III** for **4a**). Considerable caution is required to protect the integrity of the electrode surfaces which cannot be polished between scans and are cleaned after use by immersion in an ultrasonic bath containing suitable detergents.

Simulation of voltammograms was undertaken with the help of CH Instruments CV simulation and fitting program as supplied with the model 630E Electrochemical Analyzer.

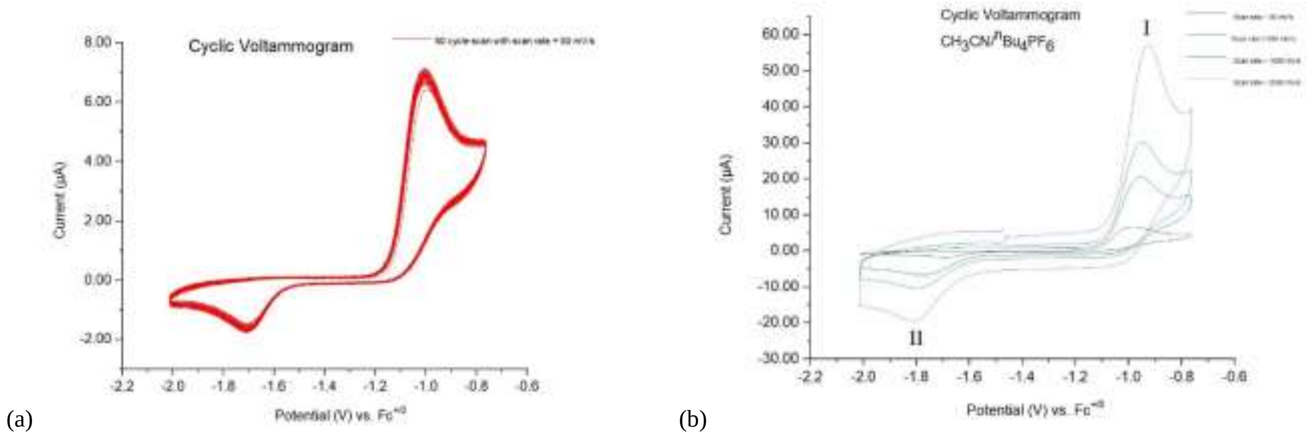

Figure S31. (a) Multicycle CV scans of 2.0 mM K[**2a**] (initial scan anodic; v = 50 mV/s) with 50 repeats. (b) Stacked plots of 2.0 mM K[**2a**] with v = 50, 500, 1000, 2500 mV/s (0.1 M ^{n}Bu$_4$NPF$_6$/CH_3CN).

Figure S31a demonstrates that the redox cycling of solutions of K[**2a**] in 0.1 M ^{n}Bu$_4$NPF$_6$/CH_3CN is robust over many cycles. Similar repeats were done at numerous scan rates. Figure S31b shows the expected scan rate dependence for EC type processes (chemically irreversible) with the concomitant drift of the peak potentials to higher anodic and lower cathodic potentials.

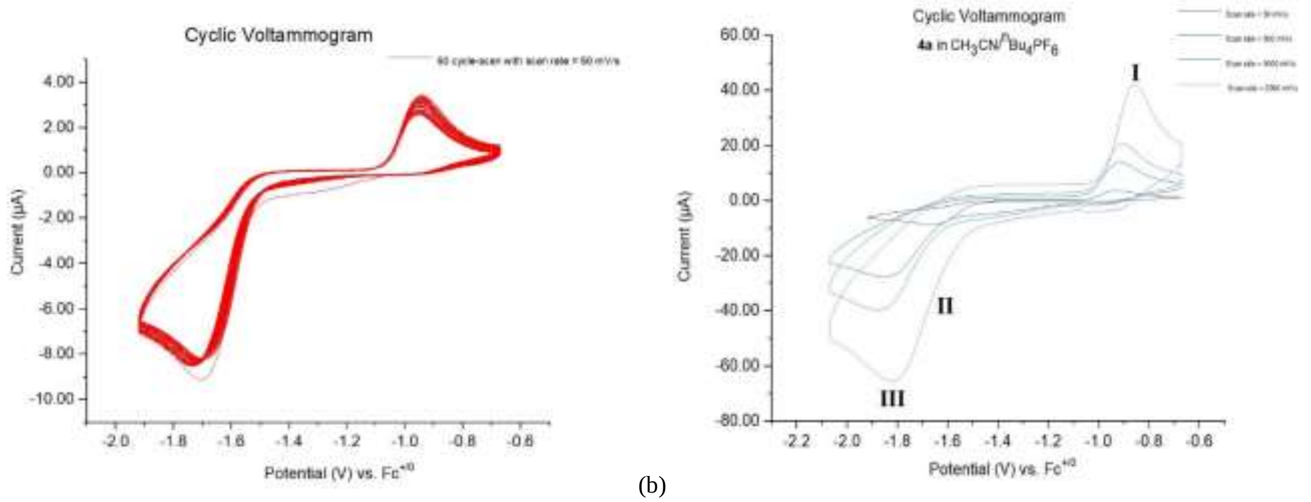

(a) (b)

Figure S32. (a) Multicycle CV scans (0.1 M $^{n}Bu_4NPF_6/CH_3CN$) of 2.0 mM **4a** (initial scan cathodic; *v* = 50 mV/s) with 50 repeats. (b) Stacked plots of **4a** with *v* = 50, 500, 1000, 2500 mV/s.

Figure S32a demonstrates that the redox cycling of solutions of **4a** in 0.1 M $^{n}Bu_4NPF_6/CH_3CN$ is also robust over many cycles. Here too, similar repeats were done at numerous scan rates. Figure S32b shows the expected scan rate dependence for EC type processes (chemically irreversible) with the concomitant drift of the peak potentials to higher anodic and lower cathodic potentials. At 2 V/s and higher, the buried cathodic process II becomes more evident, but scans to even higher scan rates become quite distorted as the capacitive currents start to dominate.

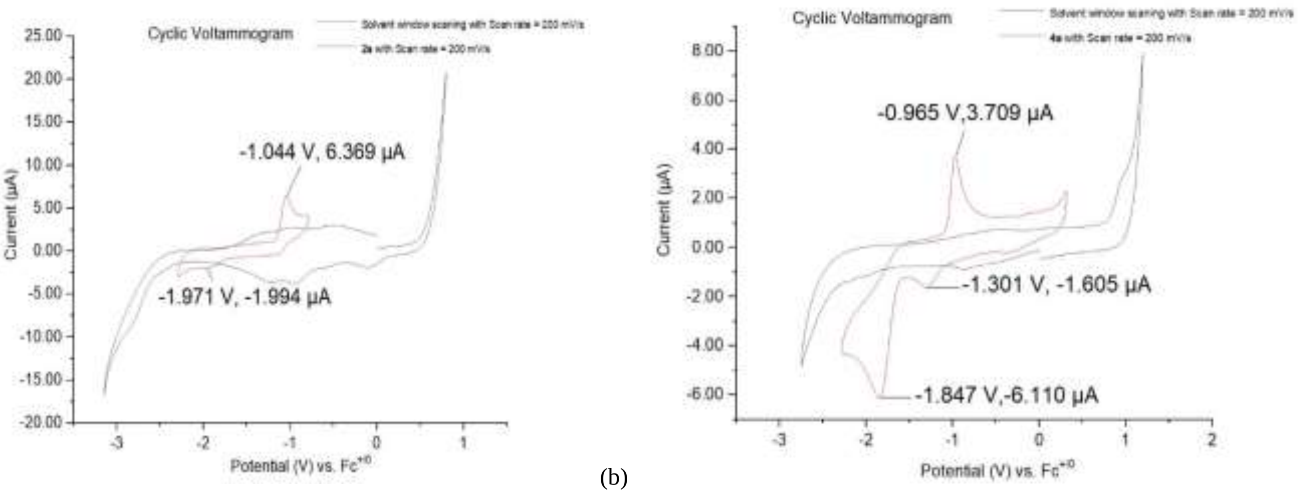

(a) (b)

Figure S33. CVs at a Pt WE (0.1 M $^{n}Bu_4NPF_6$/THF) solution of (a) 2.0 mM K[**2a**] with initial scan direction anodic (*v* = 200 mV/s); (b) 2.0 mM **4a** with initial scan direction cathodic (*v* = 200 mV/s).

The significance of Figure S33 is to show the low cathodic limiting currents available (blue traces) when THF was used as solvent. The basic processes in the voltammetry of K[**2a**] and **4a** are clearly visible (red traces) although the scans appear less stable than when measured in CH_3CN. After confirming the essential similarity of the voltammetry in THF, further work concentrated on the $^{n}Bu_4NPF_6/CH_3CN$ medium.

Digital CV simulations employing the proposed mechanism.

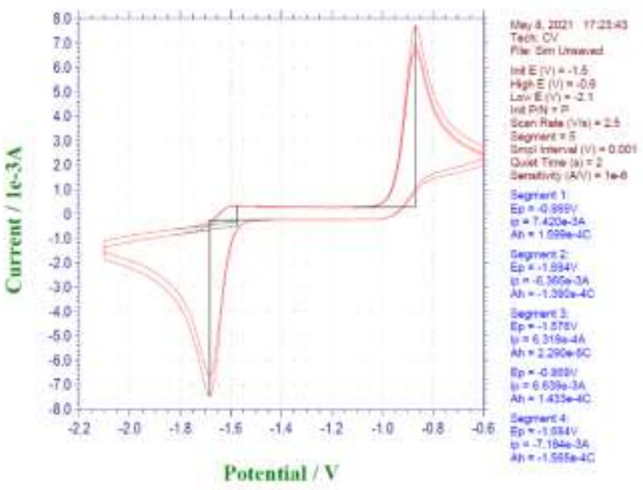

$$\text{EI}: \mathbf{2a}^- \underset{}{\overset{k_I^\circ}{\rightleftharpoons}} e^- + \mathbf{2a}^\bullet \qquad \text{(fast)}$$

$$\text{CI}: \mathbf{2a}^\bullet + \mathbf{2a}^\bullet \underset{k_{-I}}{\overset{k_I}{\rightleftharpoons}} \mathbf{4a} \qquad \text{(strongly product favoured)}$$

$$\text{EII}: \quad \mathbf{4a} + e^- \overset{k_{II}^\circ}{\rightleftharpoons} \mathbf{4a}^{\bullet -} \qquad \text{(fast)}$$

$$\text{EIII}: \quad \mathbf{4a}^{\bullet -} + e^- \overset{k_{III}^\circ}{\rightleftharpoons} \mathbf{4a}^{2-} \qquad \text{(fast)}$$

$$\text{CIII}: \quad \mathbf{4a}^{2-} \underset{k_{-III}}{\overset{k_{III}}{\rightleftharpoons}} \mathbf{2a}^- + \mathbf{2a}^- \qquad \text{(strongly product favoured)}$$

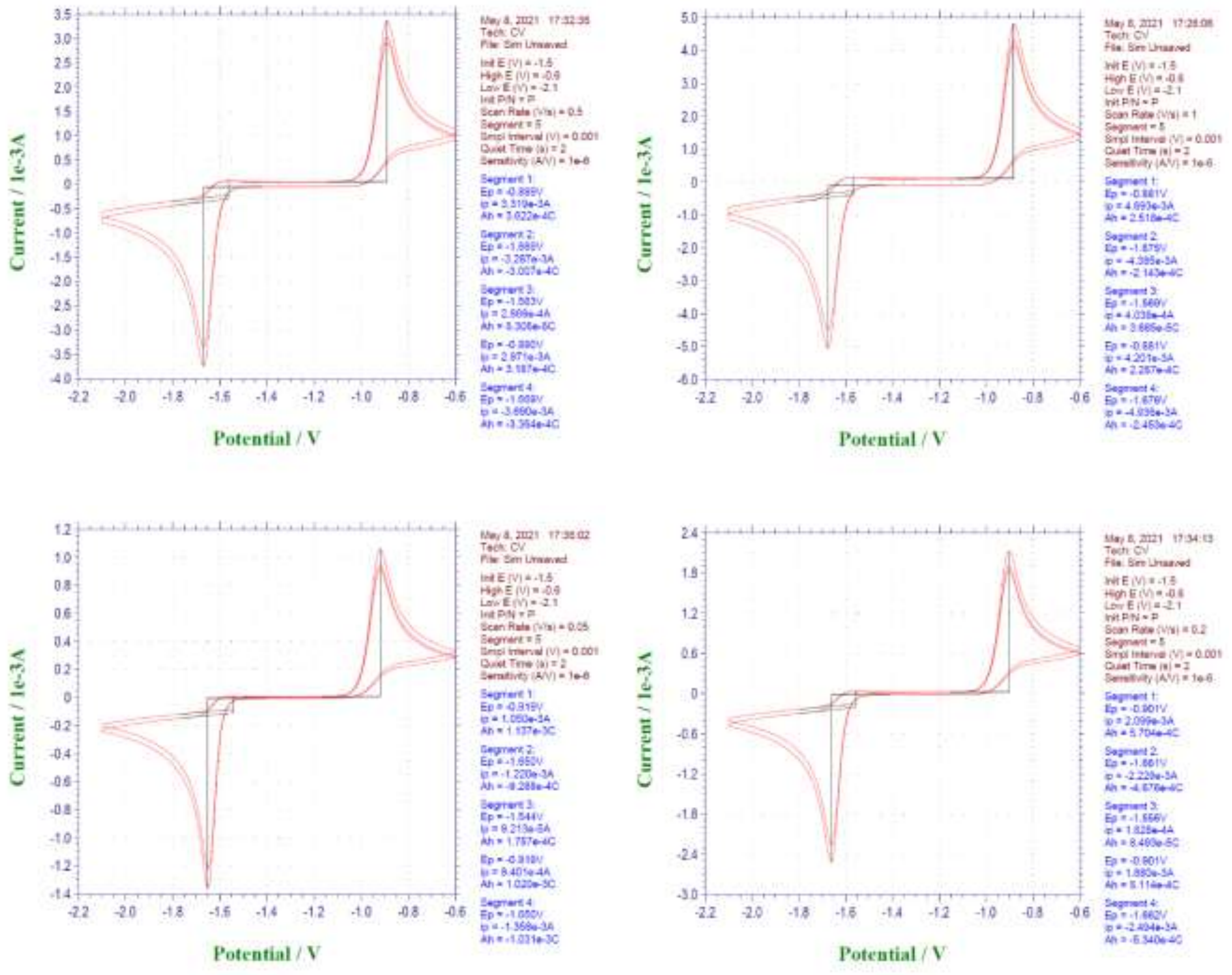

Figure S34. CV simulations with K[**2a**] as the bulk analyte (2 mM) with v = 50, 500, 1000, 2500 mV/s (increasing from bottom left). Constants employed: k_I°= 1000 s^{-1}, k_{II}°= 1000 s^{-1}, k_{III}°= 1000 s^{-1}, k_I= 10^6 M^{-1} s^{-1}, k_{III}°= 10^6 s^{-1}.

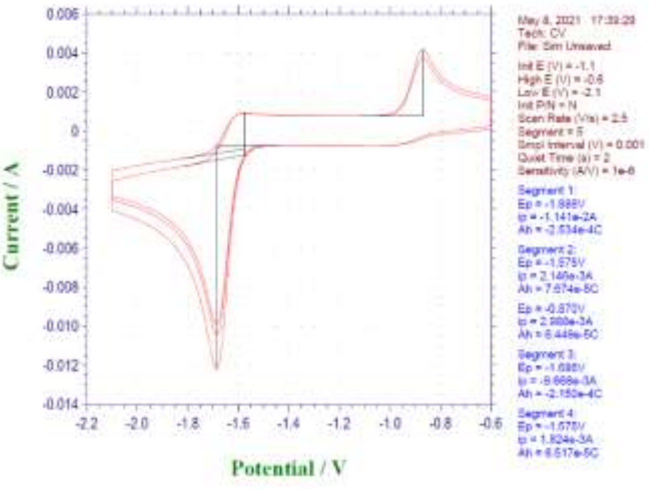

$$\text{EI}: \mathbf{2a}^- \overset{k_I^\circ}{\rightleftharpoons} e^- + \mathbf{2a}^\bullet \qquad \text{(fast)}$$

$$\text{CI}: \mathbf{2a}^\bullet + \mathbf{2a}^\bullet \underset{k_{-I}}{\overset{k_I}{\rightleftharpoons}} \mathbf{4a} \qquad \text{(strongly product favoured)}$$

$$\text{EII}: \mathbf{4a} + e^- \overset{k_{II}^\circ}{\rightleftharpoons} \mathbf{4a}^{\bullet -} \qquad \text{(fast)}$$

$$\text{EIII}: \mathbf{4a}^{\bullet -} + e^- \overset{k_{III}^\circ}{\rightleftharpoons} \mathbf{4a}^{2-} \qquad \text{(fast)}$$

$$\text{CIII}: \mathbf{4a}^{2-} \underset{k_{-III}}{\overset{k_{III}}{\rightleftharpoons}} \mathbf{2a}^- + \mathbf{2a}^- \qquad \text{(strongly product favoured)}$$

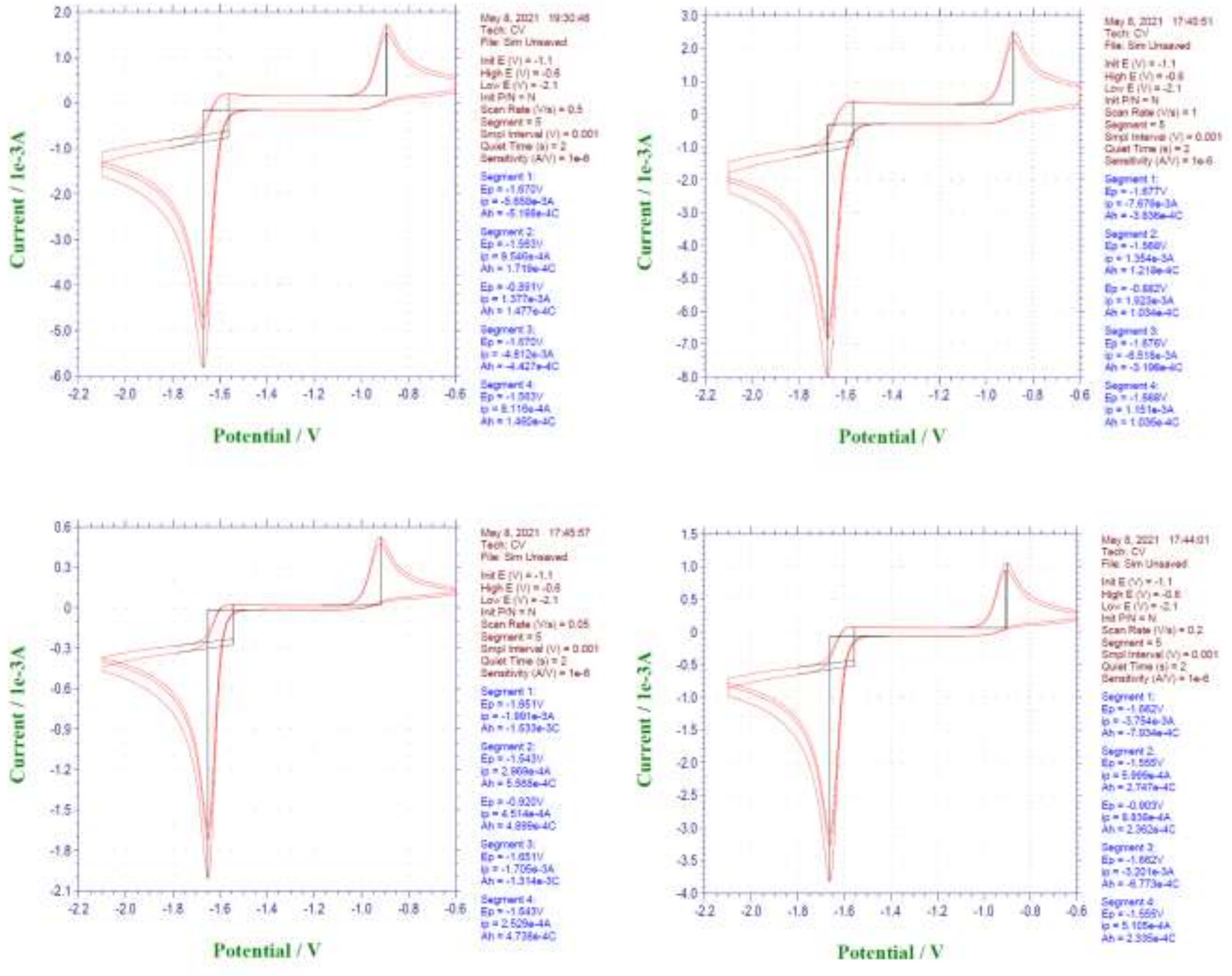

Figure S35. CV simulations with **4a** as the bulk analyte (2 mM) with v = 50, 500, 1000, 2500 mV/s (increasing from bottom left). Constants employed: k_I°= 1000 s^{-1}, k_{II}°= 1000 s^{-1}, k_{III}°= 1000 s^{-1}, k_I= 10^6 M^{-1} s^{-1}, k_{III}°= 10^6 s^{-1}.

11. Computational Details

All calculations were carried out with the Gaussian 09 program package.[1] Full geometry optimization calculations were performed at the M06-2X/6-311+G** level, followed by calculation of the second derivatives at the optimized structures to establish the nature of the stationary points obtained. The Gibbs free energies were calculated based on the harmonic vibrational frequencies (atmospheric pressure, 298.15 K). All of the tricyclic compounds were calculated with methyl substituents at the nitrogen atoms to reduce the computational time and they were labelled by the special character ‘. According to our previous results on similar systems the B3LYP/6-31G* level of theory describes properly the energy level of the orbitals, furthermore, the TD DFT calculations were in excellent agreement with the experiments for the different neutral tricyclic systems (references 20 and 21 in the main body of the text). In case of the description of the ionpairs (K[**2a’**], Li[**2b’**], K[**2c’**]) calculations were carried out with the CAM-B3LYP method as well, which contains long-range corrections, which provided good agreement with the experimental results.

Compound K[**2a’**]$^-$

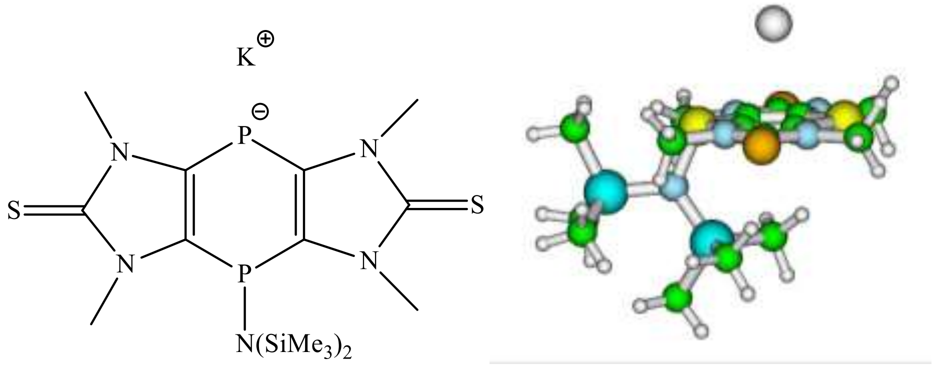

Table S2. TD-DFT results at B3LYP/6-31G*//M06-2X/6-311+G** level of theory (first 4 excited state) calculated for the contact ion pair K[**2a’**]**:**

excited state	wavelength	oscillator strength	transition	contribution
1	663 nm	0.0094	HOMO-LUMO	0.70474
2	490 nm	0.0039	HOMO-1-LUMO	0.70437
3	414 nm	0.0000	HOMO-2-LUMO	0.70391
4	403 nm	0.0004	HOMO-3-LUMO	0.70361

Table S3. TD-DFT results at cam-B3LYP/6-31G*//M06-2X/6-311+G** level of theory (first 4 excited state) calculated for the contact ion pair K[**2a’**]:

excited state	wavelength	oscillator strength	transition	contribution
1	523 nm	0.0171	HOMO-LUMO	0.68460
			HOMO-LUMO+3	0.13519
2	372 nm	0.0028	HOMO-1-LUMO	0.65763
			HOMO-1-LUMO+3	0.10860
			HOMO-LUMO+2	0.19603
3	327 nm	0.0370	HOMO-4-LUMO	-0.19815
			HOMO-LUMO+1	0.64523
			HOMO-LUMO+7	0.12519
4	320 nm	0.0009	HOMO-1-LUMO	-0.20499
			HOMO-LUMO+2	0.64541

Anion 2a’⁻

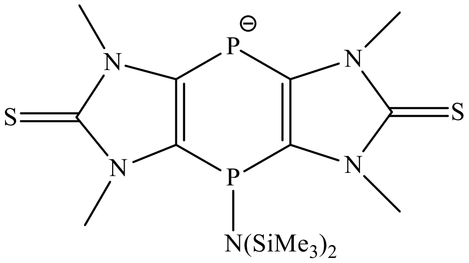

Table S4. NICS(0) and NICS(1) values of the **anion 2a’⁻** (N-Me derivative)-

	middle ring	outer ring
NICS(0)	-3.4	-9.4
NICS(1)	-4.0 (-2.7)	-7.0 (-8.2)

Table S5. TD-DFT results at B3LYP/6-311G**//M06-2X/6-311+G** level of theory (first 4 excited state) calculated for the **anion 2a’⁻.**

excited state	wavelength	oscillator strength	transition	Contribution
1	354 nm	0.1137	HOMO-LUMO	0.69524
2	326 nm	0.1735	HOMO-1-LUMO	0.10945
			HOMO-LUMO+1	0.22399
			HOMO-LUMO+2	0.65285
3	319 nm	0.0048	HOMO-LUMO+1	0.66265
			HOMO-LUMO+2	-0.22230
4	308 nm	0.0017	HOMO-LUMO+3	0.69029

Compound Li[**2b’**]

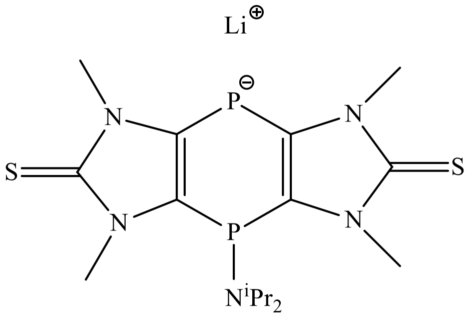

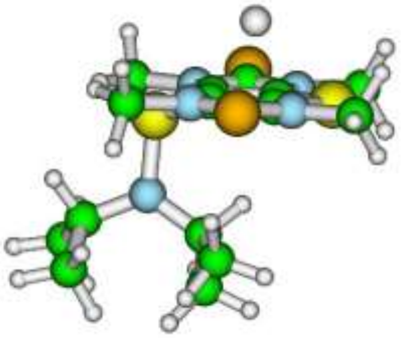

Table S6. TD-DFT results at B3LYP/6-31G*//M06-2X/6-311+G** level of theory (first 4 excited state) calculated for the contact ion pair Li[**2b’**]:

excited state	wavelength	oscillator strength	transition	contribution
1	440 nm	0.0171	HOMO-LUMO	0.69744
2	395 nm	0.0161	HOMO-1-LUMO	0.70307
3	352 nm	0.0000	HOMO-2-LUMO	0.70228
4	345 nm	0.0004	HOMO-3-LUMO	0.70170

Table S7. TD-DFT results at cam-B3LYP/6-31G*//M06-2X/6-311+G** level of theory (first 3 excited state) calculated for the contact ion pair Li[**2b'**] **:**

excited state	wavelength	oscillator strength	transition	contribution
1	393 nm	0.0231	HOMO-4-LUMO	0.15155
			HOMO-LUMO	0.68258
2	326 nm	0.0166	HOMO-1-LUMO	0.67955
			HOMO-LUMO+3	0.11274
3	283 nm	0.1594	HOMO-4-LUMO+2	0.13395
			HOMO-1-LUMO+1	-0.14579
			HOMO-LUMO+2	0.65274

Anion 2b'⁻

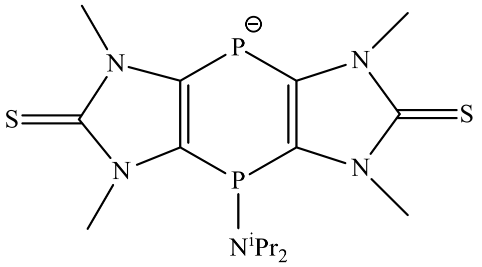

Table S8. NICS(0) and NICS(1) values of N-Me derivative **anion 2b'⁻**.

	middle ring	outer ring
NICS(0)	-4.2	-8.8
NICS(1)	-5.3 (-3.1)	-6.9 (-7.3)

Table S9. TD-DFT results at B3LYP/6-311G**//M06-2X/6-311+G** level of theory (first 4 excited state) calculated for **anion 2b'⁻**

excited state	wavelength	oscillator strength	transition	contribution
1	366 nm	0.1501	HOMO-LUMO	0.69593
2	348 nm	0.0685	HOMO-LUMO+1	0.27295
			HOMO-LUMO+2	0.63652
3	338 nm	0.0052	HOMO-LUMO+1	0.64631
			HOMO-LUMO+2	-0.27205
4	319 nm	0.0774	HOMO-LUMO+3	0.69343

Compound K[**2c'**]

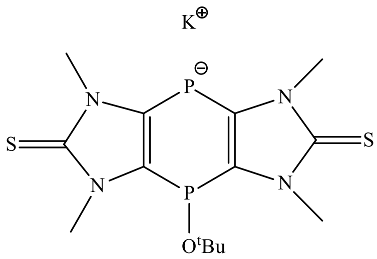

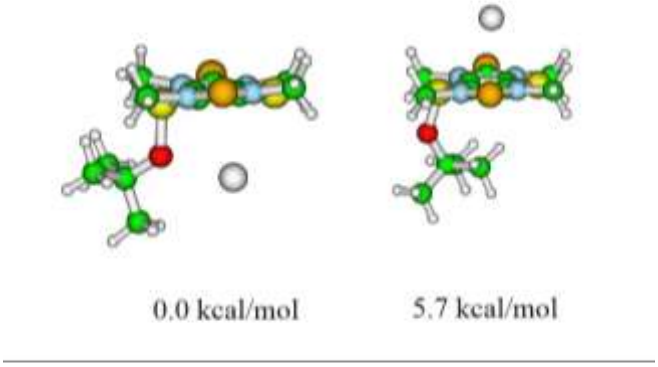

Figure S36. Possible orientation of the potassium ion and the relative energies of the systems at M06-2X/6-311+G** level of theory.

Table S10. TD-DFT results at B3LYP/6-31G*//M06-2X/6-311+G** level of theory (first 4 excited state) calculated for K[**2c'**] **.**

excited state	wavelength	oscillator strength	transition	contribution
1	519 nm	0.0082	HOMO-LUMO	0.70338
2	444 nm	0.0026	HOMO-1-LUMO	0.70401
3	376 nm	0.0003	HOMO-3-LUMO	-0.10131
			HOMO-2-LUMO	0.69677
4	368 nm	0.0006	HOMO-3-LUMO	0.69638
			HOMO-2-LUMO	0.10165

Table S11. TD-DFT results at cam-B3LYP/6-31G*//M06-2X/6-311+G** level of theory (first 4 excited state) calculated for the K[**2c'**]**.**

excited state	wavelength	oscillator strength	transition	contribution
1	419 nm	0.0178	HOMO-LUMO	0.67546
			HOMO-LUMO+4	-0.14105
2	339 nm	0.0036	HOMO-1-LUMO	0.65584
			HOMO-1-LUMO+4	-0.11876
			HOMO-LUMO+1	-0.19461
3	309 nm	0.1723	HOMO-LUMO+2	-0.19110
			HOMO-LUMO+3	0.63703
			HOMO-LUMO+4	-0.12491
4	305 nm	0.0059	HOMO-1-LUMO	0.15612
			HOMO-1-LUMO+3	0.20313
			HOMO-LUMO+1	0.56494
			HOMO-LUMO+6	-0.28714

Anion 2c’⁻

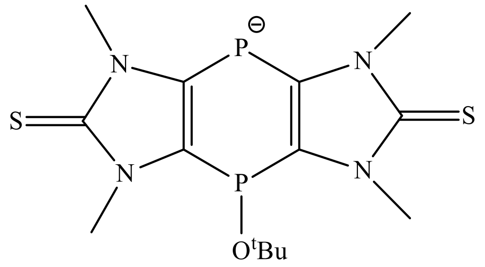

Table S12. NICS(0) and NICS(1) values of the **anion 2c’⁻** (N-Me derivative).

	middle ring	outer ring
NICS(0)	-5.2	-9.0
NICS(1)	-4.4 (-6.0)	-7.1 (-7.5)

Table S13. TD-DFT results at B3LYP/6-311G**//M06-2X/6-311+G** level of theory (first 4 excited state) calculated for **anion 2c’⁻**

excited state	wavelength	oscillator strength	transition	contribution
1	354 nm	0.1235	HOMO-LUMO	0.69214
2	325 nm	0.0990	HOMO-1-LUMO	-0.18985
			HOMO-LUMO+1	0.66483
3	301 nm	0.0048	HOMO-LUMO+1	-0.11398
			HOMO-LUMO+2	0.68490
4	294 nm	0.4978	HOMO-1-LUMO	0.65771
			HOMO-LUMO+1	0.17021
			HOMO-LUMO+2	0.12292

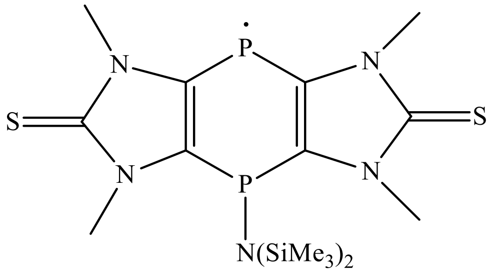

Table S14. TD-DFT results at B3LYP/6-311G**//M06-2X/6-311+G** level of theory (first 7 excited state):

excited state	wavelength	oscillator strength	transition	contribution
1	1005 nm	0.0871	β-HOMO-β-LUMO	0.99153
2	682 nm	0.0000	β-HOMO-3-β-LUMO	-0.13499
			β-HOMO-1-β-LUMO	0.98415
3	658 nm	0.0047	β-HOMO-3-β-LUMO	0.40247
			β-HOMO-2-β-LUMO	0.89461

			β-HOMO-1-<br>β-LUMO	0.13453
4	656 nm	0.0042	β-HOMO-3-<br>β-LUMO	0.90050
			β-HOMO-2-<br>β-LUMO	-0,40329
5	453 nm	0.0125	α-HOMO-<br>α-LUMO+1	0.20231
			β-HOMO-4-<br>β-LUMO	0.96571
6	401 nm	0.0117	α-HOMO-<br>α-LUMO	-0.21044
			β-HOMO-5-<br>β-LUMO	0.95549
7	389 nm	0.0079	α-HOMO-1-<br>α-LUMO+1	0.17393
			α-HOMO-<br>α-LUMO	0.88698
			β-HOMO-5-<br>β-LUMO	0.25533

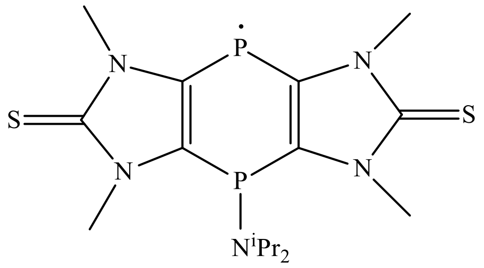

Table S15. TD-DFT results at B3LYP/6-311G**//M06-2X/6-311+G** level of theory (first 7 excited state):

excited state	Wavelength	oscillator strength	transition	contribution
1	1103 nm	0.0795	β-HOMO-<br>β-LUMO	0.99100
2	723 nm	0.0000	β-HOMO-1-<br>β-LUMO	0.99546
3	692 nm	0.0001	β-HOMO-2-<br>β-LUMO	0.99477
4	681 nm	0.0083	β-HOMO-3-<br>β-LUMO	0.98440
5	536 nm	0.0100	β-HOMO-4-<br>β-LUMO	0.99197
6	431 nm	0.0096	α-HOMO-<br>α-LUMO	-0.17077
			β-HOMO-5-<br>β-LUMO	0.97277
7	369 nm	0.0085	α-HOMO-<br>α-LUMO	0.19317
			β-HOMO-6-<br>β-LUMO	0.63261
			β-HOMO-<br>β-LUMO+1	0.25874

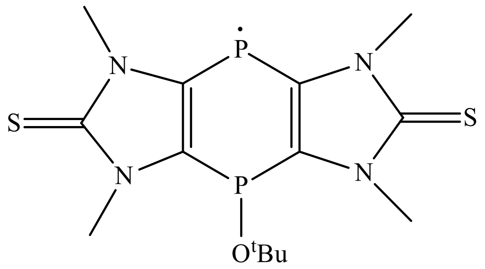

Table S16. TD-DFT results at B3LYP/6-311G**//M06-2X/6-311+G** level of theory (first 7 excited state):

excited state	Wavelength	oscillator strength	transition	contribution
1	1144 nm	0.0798	β-HOMO-β-LUMO	0.99213
2	749 nm	0.0002	β-HOMO-2-β-LUMO	-0.24751
			β-HOMO-1-β-LUMO	0.96531
3	719 nm	0.0001	β-HOMO-3-β-LUMO	0.22918
			β-HOMO-2-β-LUMO	0.93757
			β-HOMO-1-β-LUMO	0.25061
4	702 nm	0.0086	β-HOMO-3-β-LUMO	0.95913
			β-HOMO-2-β-LUMO	-0.22658
5	458 nm	0.0060	α-HOMO-α-LUMO	-0.19226
			β-HOMO-4-β-LUMO	0.96863
7	379 nm	0.0144	α-HOMO-1-α-LUMO	0.31690
			α-HOMO-α-LUMO+2	0.16144
			β-HOMO-5-β-LUMO	0.63202

Compound 4a’

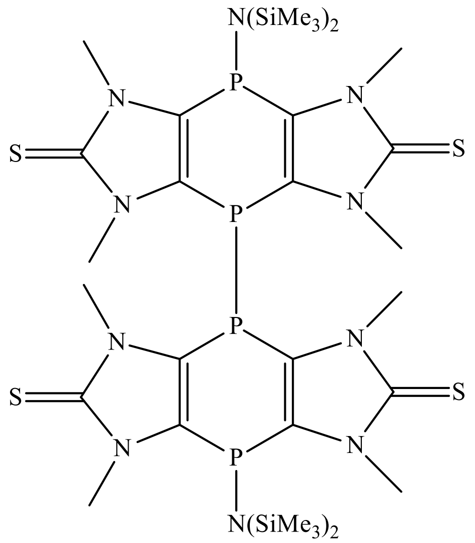

Table S17. TD-DFT results at B3LYP/6-311G**//M06-2X/6-311+G** level of theory (first 4 excited state):

excited state	wavelength	oscillator strength	transition	contribution
1	509 nm	0.1335	HOMO-LUMO	0.70062
2	486 nm	0.0065	HOMO-1-LUMO	0.70413
3	468 nm	0.0079	HOMO-2-LUMO	0.70107
4	421 nm	0.0124	HOMO-3-LUMO	0.69936

Compound 4b'

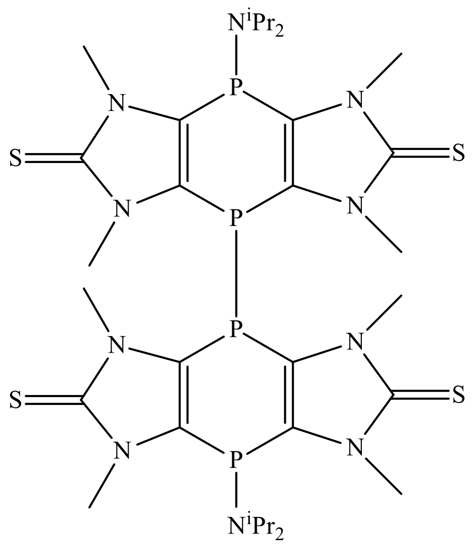

Table S18. TD-DFT results at B3LYP/6-311G**//M06-2X/6-311+G** level of theory (first 4 excited state):

excited state	wavelength	oscillator strength	transition	contribution
1	511 nm	0.1263	HOMO-LUMO	0.70189
2	482 nm	0.0091	HOMO-1-LUMO	0.70413
3	462 nm	0.0112	HOMO-2-LUMO	0.70183
4	418 nm	0.0107	HOMO-3-LUMO	0.69863

Compound 4c’

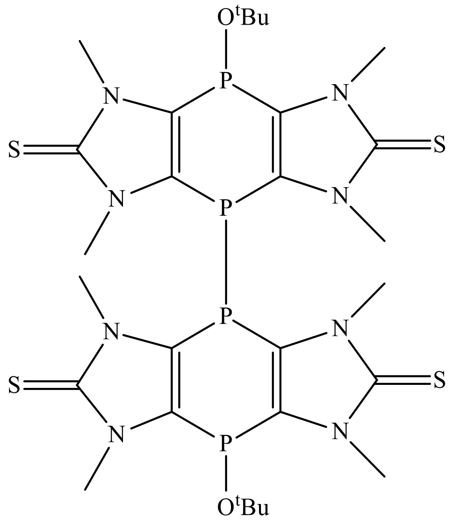

Table S19. TD-DFT results at B3LYP/6-311G**//M06-2X/6-311+G** level of theory (first 4 excited state):

excited state	wavelength	oscillator strength	transition	contribution
1	520 nm	0.0088	HOMO-LUMO	0.70487
2	497 nm	0.0810	HOMO-2-LUMO	0.50096
			HOMO-1-LUMO	-0.49651
3	493 nm	0.0651	HOMO-2-LUMO	0.49642
			HOMO-1-LUMO	0.50010
4	434 nm	0.0110	HOMO-3-LUMO	0.70048

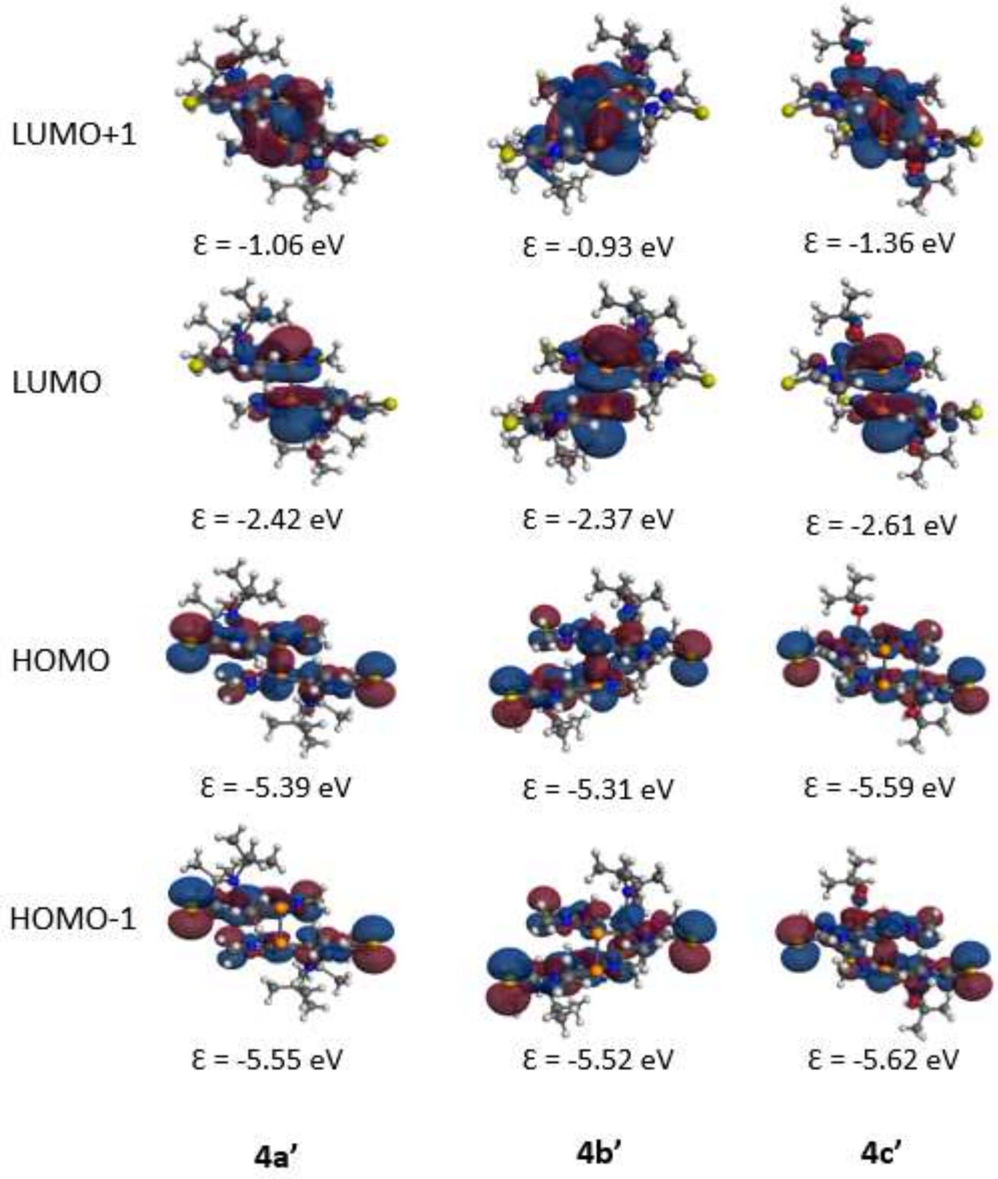

Figure S37. Kohn-Sham molecular orbitals of **4a' 4b'** and **4c'** at B3LYP/6-311G**//M06-2X/6-311+G** level of theory

Table S20. The inversion barrier for **3a' 3b' 3c'** (inversion at >P(N(SiMe$_3$)$_2$, >P(NiPr$_2$) >POtBu, respectively) at M06-2X/6-311+G** level of theory

Compound	ΔE#
**3a'**	88.5 kcal/mol
**3b'**	46.0 kcal/mol
**3c'**	73.1 kcal/mol

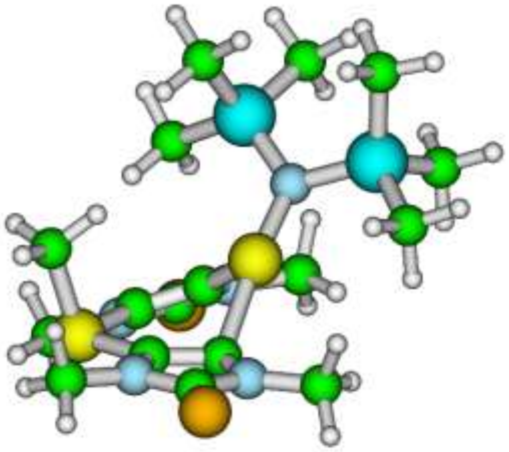

Figure S38. Optimized structure of TS of the >P($NSiMe_3$)$_2$ inversion in case of **3a'**

XYZ coordinates and total energies of the investigated system

Anion 2a'$^{-}$

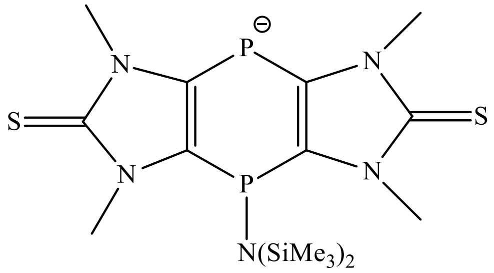

E(M06-2X/6-311+G**) = -2959.661095

N	2.375633	-2.528285	-0.084969
C	1.065993	-2.067485	-0.234674
C	1.173150	-0.805495	-0.781013
N	2.549653	-0.557784	-0.954008
C	3.285491	-1.600073	-0.508529
P	-0.326479	-3.091011	0.240639
C	-1.543104	-1.854416	-0.202299
C	-1.451347	-0.578719	-0.716680
N	-2.767843	-0.088382	-0.814230
C	-3.658089	-1.007563	-0.375659
N	-2.905236	-2.087077	-0.007510
C	-3.118168	1.215833	-1.333337
P	-0.051456	0.447919	-1.183224
N	0.132053	1.657892	0.113752
Si	0.326121	1.184440	1.811576
C	-1.031360	0.042002	2.413714
C	-3.432509	-3.315227	0.545428
S	-5.347177	-0.863461	-0.301911
C	3.127946	0.681716	-1.425866
S	4.975634	-1.717064	-0.472257
C	2.709409	-3.826964	0.457182
Si	0.291812	3.331187	-0.396306
C	0.026082	3.521257	-2.249054
C	-0.982805	4.461685	0.425142
C	2.016278	4.008773	-0.026526
C	2.010030	0.416848	2.149718
C	0.205165	2.721990	2.910556
H	2.439861	1.148104	-2.131889
H	3.309173	1.362794	-0.589725
H	4.075229	0.457463	-1.913280
H	3.793461	-3.912222	0.482019

H	2.296144	-3.919028	1.464403
H	2.280317	-4.609926	-0.172592
H	-2.845081	1.285848	-2.389103
H	-2.586292	1.988972	-0.773426
H	-4.192820	1.342324	-1.219170
H	-4.518086	-3.249990	0.537750
H	-3.067332	-3.444928	1.567300
H	-3.096056	-4.160187	-0.059595
H	2.194052	0.403224	3.228922
H	2.070248	-0.612666	1.790428
H	2.821485	0.983212	1.683002
H	-1.086254	0.113352	3.505221
H	-2.006349	0.323931	2.004292
H	-0.836615	-1.000182	2.152006
H	0.431113	2.397499	3.931696
H	0.902983	3.524431	2.661795
H	-0.806882	3.133271	2.913653
H	2.008753	5.103368	-0.022998
H	2.399511	3.671314	0.939598
H	2.724675	3.683949	-0.793389
H	0.138138	4.582243	-2.498122
H	0.745445	2.950629	-2.838499
H	-0.972969	3.202511	-2.555957
H	-1.203435	5.300698	-0.242197
H	-1.920131	3.932453	0.617117
H	-0.632434	4.874026	1.372565

anion 2b’⁻

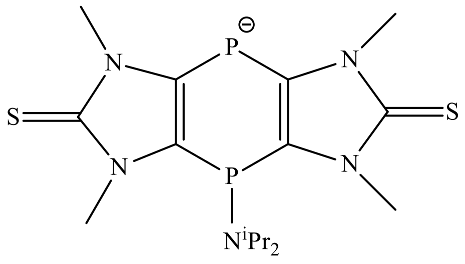

E(M06-2X/6-311+G**) = -2378.150218

N	2.601552	-1.885104	0.154767
C	1.260851	-1.517674	0.019049
C	1.260248	-0.370521	-0.746269
N	2.609151	-0.081466	-1.034041
C	3.429195	-1.007193	-0.487272
P	-0.048816	-2.483997	0.764232
C	-1.363773	-1.472394	0.080849

C	-1.371971	-0.307505	-0.661776
N	-2.730032	-0.011645	-0.915782
C	-3.536225	-0.941323	-0.362801
N	-2.701533	-1.834524	0.246006
C	-3.244696	1.099553	-1.687535
P	-0.038850	0.819116	-1.111293
N	0.065470	1.943840	0.206101
C	0.075420	3.367529	-0.130795
C	1.200230	4.165155	0.537196
C	-3.132144	-3.016042	0.958737
S	-5.230111	-0.975931	-0.431708
C	3.049537	1.062067	-1.801882
S	5.123400	-1.076715	-0.577157
C	3.036836	-3.056276	0.882313
C	0.178561	1.432905	1.580520
C	-0.776342	2.099232	2.573649
C	1.616177	1.407243	2.122987
C	-1.275826	4.072275	0.074019
H	2.669280	1.979672	-1.344411
H	4.137551	1.065198	-1.801824
H	2.674296	0.994811	-2.825201
H	4.118797	-3.124663	0.795908
H	2.745045	-2.967002	1.931691
H	2.561846	-3.944756	0.459631
H	-3.857749	1.744179	-1.054197
H	-3.872093	0.723277	-2.497100
H	-2.404143	1.658785	-2.095343
H	-4.217969	-3.060493	0.910176
H	-2.800963	-2.961268	1.998625
H	-2.691909	-3.902554	0.496187
H	-0.137833	0.388925	1.520204
H	-1.262443	5.055350	-0.407671
H	-2.078957	3.480469	-0.368898
H	-1.505188	4.220597	1.130236
H	1.240366	5.170637	0.108407
H	1.034861	4.271827	1.611671
H	2.167678	3.682161	0.387340
H	-0.743996	1.555187	3.521247
H	-0.501065	3.136900	2.780443
H	-1.801336	2.074462	2.196993
H	1.659804	0.757939	3.002229
H	2.302581	1.004107	1.374655
H	1.966909	2.397831	2.421052

H	0.270276	3.394067	-1.209581

anion 2c’⁻

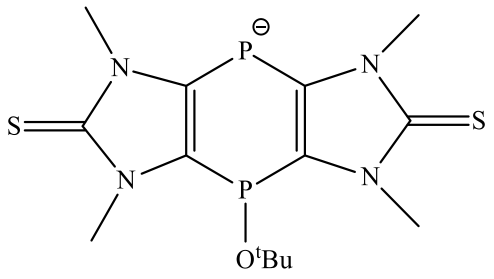

E(M06-2X/6-311+G**) = -2319.437711

N	0.000000	0.000000	0.000000
C	0.000000	0.000000	1.400652
C	1.332151	0.000000	1.787073
N	2.093193	-0.001329	0.592200
C	1.282188	0.006601	-0.498375
P	-1.520883	0.016294	2.342165
C	-0.730044	0.176033	3.939025
C	0.605042	0.197183	4.320519
N	0.615603	0.423298	5.719685
C	-0.650296	0.542788	6.200641
N	-1.472538	0.380691	5.109715
C	1.810546	0.590360	6.526177
P	2.122965	-0.085885	3.399688
O	2.618530	-1.721493	3.650471
C	1.935162	-3.002050	3.725775
C	1.300245	-3.155568	5.115064
C	-2.919652	0.454253	5.154174
S	-1.128153	0.840223	7.805498
C	3.542718	0.041627	0.528849
S	1.742830	0.030719	-2.135897
C	-1.197741	0.019460	-0.816202
C	0.881990	-3.180123	2.627495
C	3.059502	-4.030811	3.545836
H	3.961984	-0.662118	1.249969
H	3.841218	-0.226199	-0.483531
H	3.911528	1.045981	0.758590
H	-0.889496	0.069046	-1.859188
H	-1.788112	-0.885137	-0.644451
H	-1.808693	0.889680	-0.560490
H	2.543677	-0.173500	6.261859
H	1.524383	0.497162	7.572633
H	2.253893	1.577170	6.360981

H	-3.211323	0.673964	6.179777
H	-3.357262	-0.496612	4.837005
H	-3.272621	1.242272	4.482985
H	0.473189	-4.195358	2.675787
H	0.052615	-2.479540	2.738333
H	1.323309	-3.037377	1.638256
H	0.864647	-4.153867	5.230533
H	2.056552	-3.018456	5.892638
H	0.509867	-2.419775	5.268413
H	2.670630	-5.051230	3.631308
H	3.522807	-3.916183	2.562671
H	3.830238	-3.884518	4.306735

compound 3a’

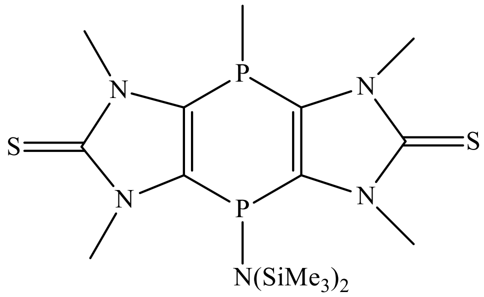

isomer cis:

E(M06-2X/6-311+G**) = -2999.482043

N	-2.682943	-0.011831	-0.807892
C	-1.379190	-0.495494	-0.815661
C	-1.448614	-1.818248	-0.495404
N	-2.791836	-2.115813	-0.291292
C	-3.559310	-0.994071	-0.444463
P	0.021740	0.587508	-1.246025
N	0.066230	1.744234	0.036467
Si	0.245016	3.453020	-0.446551
C	-1.149932	4.498905	0.263774
P	-0.157583	-3.087300	-0.326375
C	1.250429	-1.948744	-0.479735
C	1.308257	-0.626345	-0.801990
N	2.652083	-0.268515	-0.785475
C	3.429749	-1.328113	-0.413568
N	2.557508	-2.371659	-0.264114
C	3.196530	1.050899	-1.044729
S	5.084929	-1.351235	-0.172753
C	2.994627	-3.707901	0.098204
C	-3.359954	-3.403926	0.063190

S	-5.210586	-0.854637	-0.217385
C	-3.110263	1.351739	-1.060654
Si	0.018224	1.293528	1.765608
C	1.559461	0.341673	2.264973
C	-0.049815	2.844555	2.829705
C	-1.536429	0.341601	2.220718
C	1.913021	4.130395	0.110376
C	0.169485	3.620372	-2.314820
H	2.543636	1.576259	-1.742194
H	3.280365	1.621255	-0.117606
H	4.187335	0.930650	-1.479706
H	3.917125	-3.927534	-0.437171
H	3.193502	-3.768844	1.170283
H	2.223593	-4.424022	-0.186727
H	-2.348950	1.864149	-1.646859
H	-3.271407	1.880382	-0.118410
H	-4.050236	1.322043	-1.609610
H	-4.287842	-3.537512	-0.491075
H	-3.587028	-3.441679	1.130751
H	-2.654392	-4.190737	-0.203995
H	1.570149	0.234646	3.354212
H	1.619834	-0.657707	1.831341
H	2.469706	0.879137	1.982090
H	-1.592637	0.294276	3.313278
H	-2.434452	0.861679	1.873749
H	-1.576271	-0.678127	1.839085
H	0.117474	2.520530	3.861963
H	0.700844	3.603635	2.605049
H	-1.035578	3.312275	2.794371
H	1.841248	5.204689	0.303523
H	2.282502	3.653683	1.021048
H	2.668175	3.987893	-0.666925
H	0.305369	4.682075	-2.546818
H	0.951067	3.063539	-2.834632
H	-0.789616	3.311163	-2.736495
H	-1.407728	5.286571	-0.450024
H	-2.050494	3.905723	0.440222
H	-0.875962	4.982362	1.202391
C	-0.167969	-3.310701	1.523816
H	0.646089	-3.982483	1.801762
H	-0.053703	-2.362650	2.048734
H	-1.106718	-3.782653	1.819577

isomer trans:

E(M06-2X/6-311+G**) = -2999.483718

N	-2.687770	-0.199536	-0.617992
C	-1.366783	-0.629704	-0.626006
C	-1.370933	-1.926484	-0.206937
N	-2.693971	-2.254780	0.075303
C	-3.517500	-1.194704	-0.177986
P	-0.025826	0.486025	-1.156521
N	-0.085204	1.779049	-0.017320
Si	-0.061422	3.432019	-0.689378
C	-1.660059	4.364093	-0.366360
P	-0.005672	-3.049519	0.196155
C	1.339776	-1.864388	-0.102571
C	1.332564	-0.580997	-0.567155
N	2.663015	-0.177117	-0.641039
C	3.493986	-1.167019	-0.200474
N	2.668214	-2.207430	0.124534
C	3.166083	1.122049	-1.047458
S	5.160565	-1.113997	-0.072791
C	3.175370	-3.480076	0.606203
C	-3.198906	-3.544525	0.510691
S	-5.180361	-1.141852	0.009059
C	-3.155631	1.134086	-0.949069
Si	0.055858	1.541045	1.750538
C	1.860404	1.444534	2.254582
C	-0.755710	2.982739	2.645728
C	-0.831320	0.014036	2.380801
C	1.393270	4.381434	0.028268
C	0.141556	3.395962	-2.555284
H	2.387067	1.651089	-1.592629
H	3.468704	1.698415	-0.170799
H	4.036079	0.973791	-1.686252
H	3.961381	-3.287465	1.335191
H	2.361866	-4.033344	1.074225
H	3.601789	-4.055846	-0.217756
H	-2.552130	1.534697	-1.765014
H	-3.078161	1.792594	-0.081188
H	-4.196983	1.056387	-1.256309
H	-3.959827	-3.381176	1.272302
H	-2.376783	-4.126071	0.926520
H	-3.654977	-4.076323	-0.326826
H	1.929867	1.381096	3.344829
H	2.360766	0.563587	1.843383
H	2.416582	2.331627	1.941082

H	-0.871718	0.107210	3.471317
H	-1.860081	-0.067676	2.019778
H	-0.302176	-0.911739	2.151497
H	-0.561973	2.849681	3.714822
H	-0.392859	3.973800	2.371401
H	-1.840331	2.963626	2.509740
H	1.396529	5.398129	-0.375840
H	1.375224	4.461632	1.116612
H	2.338833	3.911747	-0.255380
H	0.152062	4.435320	-2.899889
H	1.065409	2.925404	-2.896575
H	-0.687415	2.890384	-3.055262
H	-1.514293	5.413525	-0.641351
H	-2.469513	3.978012	-0.990857
H	-1.990172	4.338222	0.671734
C	0.104003	-4.046600	-1.367962
H	1.003983	-4.663984	-1.333364
H	-0.761830	-4.709616	-1.422694
H	0.131189	-3.401522	-2.245937

TS of the >P-N($SiMe_3$)$_2$ inversion

E(M06-2X/6-311+G**) = -2999.342659

C	-0.205874	2.134850	0.019860
C	0.373800	0.977999	-0.439419
N	1.517559	1.360526	-1.133224
C	1.685026	2.718076	-1.071196
N	0.610477	3.182773	-0.365606
P	-0.157766	-0.752337	-0.196162
N	1.484442	-1.336746	0.184532
Si	1.756539	-3.043324	-0.299882
C	2.153097	-4.157245	1.159476
C	2.369385	0.527628	-1.967118
S	2.946008	3.624381	-1.688776
C	0.459090	4.587047	-0.024243
P	-1.851630	2.359018	0.744672
C	-1.533975	1.774267	2.482965
C	-2.050382	-0.064412	-0.873124
N	-3.076267	-0.971049	-1.070146
C	-4.183127	-0.689647	-0.314429
N	-3.859525	0.451275	0.367381
C	-2.572829	0.859162	0.021819
C	-2.995882	-2.149830	-1.910962

C	-4.767858	1.091083	1.300677
S	-5.601221	-1.565276	-0.217459
Si	2.655960	-0.659682	1.380869
C	4.401315	-1.302166	1.090142
C	2.847972	1.201537	1.487978
C	2.018512	-1.213618	3.060134
C	0.225374	-3.762865	-1.111344
C	3.126062	-3.098953	-1.583177
H	-2.087439	-2.071819	-2.504406
H	-2.975974	-3.055484	-1.301371
H	-3.869682	-2.192789	-2.561591
H	-4.497490	2.142844	1.399760
H	-5.781158	1.008757	0.910237
H	-4.732106	0.598761	2.275315
H	1.782581	-0.294782	-2.370932
H	3.210019	0.122856	-1.399188
H	2.752124	1.153781	-2.770820
H	1.219528	4.874524	0.703953
H	-0.537791	4.742992	0.383336
H	0.597111	5.186256	-0.923106
H	2.704290	-0.895209	3.850562
H	1.047316	-0.748706	3.255352
H	1.890239	-2.293636	3.134895
H	3.451217	1.366504	2.387830
H	3.392054	1.644452	0.651113
H	1.907894	1.739308	1.626326
H	5.020558	-0.935478	1.915242
H	4.503280	-2.387054	1.062086
H	4.825642	-0.895008	0.168456
H	2.338750	-5.165803	0.777310
H	3.024395	-3.861767	1.744469
H	1.294385	-4.216037	1.833670
H	0.453347	-4.801077	-1.375018
H	-0.644740	-3.767583	-0.452188
H	-0.048258	-3.245451	-2.033820
H	3.313659	-4.138984	-1.867435
H	2.823957	-2.563710	-2.487422
H	4.067735	-2.672291	-1.236837
H	-2.482880	1.698504	3.016572
H	-1.036681	0.802988	2.483526
H	-0.911551	2.511384	2.993625

compound 3b'

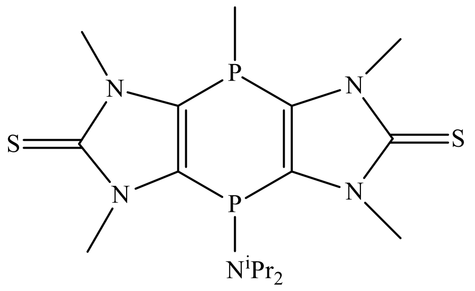

isomer cis:

E(M06-2X/6-311+G**) = -2417.972146

N	2.658299	-1.860930	-0.101790
C	1.326808	-1.476188	-0.210313
C	1.314588	-0.225378	-0.747830
N	2.643928	0.136298	-0.947352
C	3.484862	-0.860516	-0.536538
P	-0.035252	-2.598800	0.208035
C	-1.387458	-1.449121	-0.185060
C	-1.384116	-0.187143	-0.707113
N	-2.719216	0.167953	-0.888116
C	-3.548290	-0.830652	-0.466275
N	-2.716058	-1.838434	-0.063845
C	-3.227936	1.417014	-1.423925
P	-0.024513	0.957689	-1.132560
N	0.020297	2.038201	0.186036
C	0.173126	3.473032	-0.140922
C	1.256072	4.185638	0.672498
C	-3.213475	-3.096573	0.462190
S	-5.220850	-0.822422	-0.431415
C	3.089939	1.388495	-1.531886
S	5.159088	-0.882640	-0.542575
C	3.160730	-3.117729	0.423034
C	-0.001316	1.527334	1.568779
C	-1.007801	2.241355	2.470907
C	1.380691	1.428678	2.229846
C	-1.139730	4.261842	-0.086526
H	2.660103	2.226294	-0.981014
H	4.175448	1.415621	-1.460896
H	2.786304	1.443681	-2.578678
H	3.994958	-3.445286	-0.195525
H	3.518125	-2.988510	1.446660
H	2.363270	-3.860275	0.390325
H	-3.568524	2.066611	-0.615128

H	-4.074641	1.195049	-2.072082
H	-2.438391	1.904968	-1.993408
H	-4.074545	-3.403360	-0.129502
H	-3.531476	-2.977879	1.500232
H	-2.428291	-3.849109	0.389366
H	-0.368838	0.502635	1.471928
H	-0.979386	5.269457	-0.479037
H	-1.904798	3.779771	-0.695014
H	-1.518140	4.356422	0.931538
H	1.417196	5.180514	0.251485
H	0.958738	4.317871	1.714376
H	2.204636	3.647402	0.653050
H	-1.082058	1.704065	3.419636
H	-0.705299	3.265044	2.699144
H	-1.997133	2.261098	2.009035
H	1.316370	0.775455	3.105164
H	2.116127	1.001571	1.543967
H	1.746854	2.396104	2.574008
H	0.501373	3.489552	-1.185957
C	-0.010146	-2.484624	2.065115
H	0.854614	-3.037724	2.436917
H	0.041996	-1.452820	2.411515
H	-0.909968	-2.960970	2.459202

isomer trans:

E(M06-2X/6-311+G**) = -2417.972477

N	-2.741491	-1.701782	0.280191
C	-1.410176	-1.350995	0.096782
C	-1.394758	-0.159954	-0.571866
N	-2.727919	0.181679	-0.796682
C	-3.564782	-0.761675	-0.277363
P	-0.066722	-2.388883	0.747384
C	-0.122060	-3.771816	-0.495776
P	-0.022998	0.925933	-1.096501
N	0.079830	2.096501	0.137460
C	0.114592	1.689371	1.555057
C	1.521561	1.669200	2.168931
C	-3.231165	1.349652	-1.496659
S	-5.237115	-0.774867	-0.320969
C	-3.250079	-2.898038	0.926543
C	1.301074	-1.420294	0.052540
N	2.629674	-1.794332	0.215901
C	3.462555	-0.884552	-0.378621
N	2.630043	0.069584	-0.893349

C	1.298613	-0.246774	-0.639109
C	3.122379	-2.996087	0.864656
C	3.083121	1.246946	-1.612518
S	5.133784	-0.961395	-0.451113
C	0.186614	3.505020	-0.299875
C	-1.137536	4.275285	-0.240946
C	1.292633	4.292427	0.405516
C	-0.873694	2.454191	2.435311
H	2.738401	2.147539	-1.100907
H	4.170760	1.223101	-1.628457
H	2.695270	1.234420	-2.632205
H	4.008751	-2.740134	1.443024
H	2.348495	-3.385350	1.526258
H	3.399234	-3.746175	0.120781
H	-3.661727	2.058595	-0.787102
H	-4.013587	1.032242	-2.185541
H	-2.413434	1.812653	-2.046205
H	-4.138374	-2.629899	1.496578
H	-2.486610	-3.295328	1.595483
H	-3.529614	-3.646718	0.182233
H	-0.231541	0.652351	1.556105
H	-1.023393	5.240344	-0.741743
H	-1.928862	3.721938	-0.747015
H	-1.454497	4.467284	0.784253
H	1.416317	5.255290	-0.095186
H	1.042326	4.497342	1.447982
H	2.247612	3.765959	0.379484
H	-0.907324	1.988433	3.422973
H	-0.579603	3.496078	2.575505
H	-1.877713	2.426048	2.006473
H	1.504323	1.076570	3.087489
H	2.241350	1.211255	1.486422
H	1.876434	2.666866	2.428677
H	0.466078	3.448172	-1.357640
H	0.755001	-4.406912	-0.357839
H	-1.010892	-4.379382	-0.317071
H	-0.138379	-3.381134	-1.513034

TS of the >P-N(Ipr)$_2$ inversion

E(M06-2X/6-311+G**) = -2417.899109

C	-1.433367	-0.302016	-0.014249
C	-1.394245	-1.663524	0.142894
N	-2.674326	-2.129328	-0.177786
C	-3.504958	-1.094096	-0.467676
N	-2.726997	0.036262	-0.371053
P	-0.069299	-2.733408	0.771919
C	-0.096183	-2.252801	2.566796
C	-3.083582	-3.520604	-0.140044
S	-5.136489	-1.125097	-0.850388
C	-3.280301	1.367271	-0.530702
P	0.009676	0.710863	0.119853
N	0.053359	2.361223	0.195840
C	-0.140687	3.007838	1.532384
C	1.176440	3.347374	2.230408
C	1.330067	-1.723143	0.203823
C	1.411336	-0.363533	0.052541
N	2.699366	-0.065526	-0.359578
C	3.434731	-1.220596	-0.484982
N	2.583499	-2.228155	-0.162333
C	3.002317	-3.618167	-0.141643
S	5.044881	-1.355788	-0.926260
C	3.230964	1.254285	-0.627910
C	0.342852	3.151249	-1.045268
C	-0.918849	3.625652	-1.764945
C	1.331190	4.301007	-0.842852
C	-1.106749	4.191486	1.509175
H	2.787531	1.960918	0.071263
H	4.310277	1.214549	-0.489891
H	3.017684	1.558216	-1.656026
H	3.464580	-3.865211	-1.096924
H	3.739022	-3.774037	0.648324
H	2.128095	-4.243806	0.026144
H	-4.200111	1.435239	0.050532
H	-3.522548	1.557149	-1.577720
H	-2.549971	2.090161	-0.175530
H	-4.092807	-3.576196	-0.542443
H	-3.080944	-3.890566	0.887791
H	-2.402623	-4.121091	-0.743011
H	-0.622325	2.233451	2.132689
H	-0.637022	4.171013	-2.668640
H	-1.528702	2.774850	-2.069335

H	-1.522704	4.290361	-1.145430
H	1.577873	4.708678	-1.825496
H	0.919491	5.117199	-0.249316
H	2.259223	3.969973	-0.378393
H	-1.268939	4.523918	2.536855
H	-0.725313	5.043780	0.947484
H	-2.075150	3.907405	1.094258
H	0.977919	3.603692	3.273897
H	1.844544	2.482339	2.221299
H	1.686343	4.194701	1.772454
H	0.832638	2.438843	-1.711162
H	0.769419	-2.698261	3.061343
H	-0.071582	-1.168706	2.690139
H	-1.003998	-2.653651	3.022497

compound 3c’

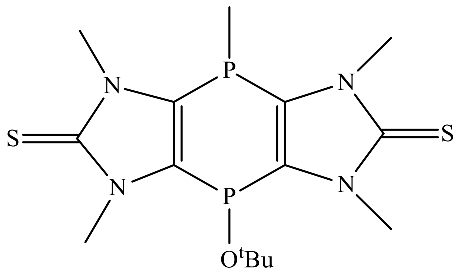

isomer cis:

E(M06-2X/6-311+G**) = -2359.251904

N	-0.010139	-0.039128	-0.043311
C	-0.015352	0.066528	1.341618
C	1.273785	-0.080723	1.758828
N	2.043606	-0.282438	0.617085
C	1.257790	-0.284791	-0.498764
P	-1.537445	0.420151	2.268722
C	-0.817022	0.227465	3.927645
C	0.479261	0.087219	4.333623
N	0.470628	0.156904	5.724589
C	-0.801610	0.308719	6.192953
N	-1.587583	0.380200	5.072567
C	1.615023	0.058687	6.616163
P	2.046584	0.076366	3.402415
O	2.735455	-1.390651	3.687466
C	2.229873	-2.758252	3.729100
C	1.657069	-3.020463	5.118011
C	-3.027990	0.546494	5.136163
S	-1.292978	0.375174	7.788290

C	3.475653	-0.527846	0.548561
S	1.740143	-0.546833	-2.077383
C	-1.153290	0.064523	-0.931492
C	1.185964	-3.024102	2.651683
C	3.473524	-3.604320	3.489092
H	3.892065	-0.513966	1.553628
H	3.651923	-1.500685	0.086406
H	3.946470	0.236502	-0.069869
H	-0.841087	0.585646	-1.835280
H	-1.514797	-0.927362	-1.210846
H	-1.941844	0.629831	-0.433977
H	2.501181	-0.190385	6.034793
H	1.423572	-0.719887	7.355512
H	1.757742	1.005574	7.138108
H	-3.259868	1.213189	5.965102
H	-3.518645	-0.412880	5.313942
H	-3.378933	0.984497	4.201323
H	0.912637	-4.081473	2.683479
H	0.287308	-2.432063	2.825399
H	1.572139	-2.799804	1.654537
H	1.385482	-4.074455	5.212663
H	2.402296	-2.787998	5.881329
H	0.761087	-2.422927	5.300192
H	3.225780	-4.666702	3.543900
H	3.888056	-3.390651	2.501837
H	4.231301	-3.379092	4.241220
C	-2.436155	-1.199329	2.090692
H	-2.799987	-1.287645	1.065657
H	-1.807052	-2.055192	2.329384
H	-3.303746	-1.188211	2.752881

isomer trans:

E(M06-2X/6-311+G**) = -2359.253401

N	0.011751	-0.120026	-0.099110
C	0.009686	0.037421	1.280741
C	1.303913	-0.067739	1.695542
N	2.074708	-0.279874	0.557211
C	1.290066	-0.293177	-0.560939
P	-1.533401	0.167449	2.225501
C	-0.771648	0.256428	3.872918
C	0.531561	0.172370	4.270986
N	0.533313	0.296287	5.658204
C	-0.736729	0.448579	6.131091
N	-1.538274	0.412212	5.019406

C	1.686327	0.241962	6.542372
P	2.088080	0.063994	3.333967
O	2.676794	-1.442908	3.635681
C	2.066534	-2.766025	3.723911
C	1.477561	-2.938827	5.120383
C	-2.980149	0.566456	5.094137
S	-1.212769	0.641125	7.720722
C	3.521448	-0.427638	0.512413
S	1.772819	-0.462316	-2.152723
C	-1.134053	-0.033786	-0.986299
C	1.008441	-2.999667	2.650603
C	3.246431	-3.707975	3.517997
H	3.866358	-0.862993	1.449446
H	3.767147	-1.084943	-0.319955
H	3.997715	0.540974	0.349092
H	-1.011006	-0.766926	-1.781735
H	-2.040134	-0.250407	-0.420293
H	-1.195445	0.959525	-1.436237
H	2.563637	-0.051263	5.968037
H	1.494896	-0.489033	7.328489
H	1.844966	1.216820	7.005202
H	-3.341888	0.013221	5.959622
H	-3.429396	0.167275	4.184856
H	-3.246315	1.618102	5.219721
H	0.703665	-4.048104	2.689727
H	0.121146	-2.386611	2.816336
H	1.402928	-2.796534	1.652144
H	1.146905	-3.971346	5.255188
H	2.234569	-2.718677	5.876081
H	0.615971	-2.286677	5.276964
H	2.924596	-4.746813	3.618205
H	3.670742	-3.566933	2.521801
H	4.021423	-3.505026	4.258853
C	-1.924947	1.966371	1.967040
H	-2.234118	2.113596	0.930489
H	-2.759372	2.246900	2.612315
H	-1.060215	2.592178	2.186449

TS of the >P-O^{t}Bu inversion

E(M06-2X/6-311+G**) = -2359.136832

N	0.000000	0.000000	0.000000
C	0.000000	0.000000	1.371457
C	1.329273	0.000000	1.792770

N	2.088937	0.050935	0.616227
C	1.289097	0.033668	-0.485309
P	-1.596854	0.081783	2.236605
C	-2.044709	-1.723677	2.250570
P	1.944440	-0.428332	3.447145
O	3.354303	-1.111480	2.641396
C	4.432107	-1.717918	3.366092
C	4.921546	-0.807540	4.498307
C	3.505327	0.382418	0.472729
S	1.733560	0.069861	-2.090988
C	-1.156368	-0.068603	-0.878255
C	-0.951224	0.297345	3.893221
N	-1.728763	0.347702	5.030999
C	-0.923500	0.529706	6.131727
N	0.343713	0.601899	5.631550
C	0.372429	0.450405	4.245772
C	-3.166947	0.179213	5.119569
C	1.507836	0.784386	6.480792
S	-1.415021	0.616771	7.724154
C	3.960269	-3.063524	3.922132
C	5.554484	-1.929263	2.353794
H	2.315290	1.199967	5.880197
H	1.247056	1.468250	7.287645
H	1.821072	-0.167895	6.915133
H	-3.548442	0.852151	5.885944
H	-3.612268	0.426534	4.154996
H	-3.418670	-0.845599	5.401131
H	3.850410	0.869138	1.379175
H	3.593613	1.050743	-0.382165
H	4.091624	-0.516999	0.299778
H	-0.949910	0.534999	-1.759891
H	-2.028733	0.319707	-0.353824
H	-1.330560	-1.098716	-1.194918
H	5.831445	-1.221668	4.939224
H	5.150226	0.187272	4.106195
H	4.173534	-0.710130	5.286977
H	6.398286	-2.445510	2.818728
H	5.194505	-2.530211	1.516045
H	5.906208	-0.968978	1.969447
H	4.784985	-3.582064	4.417340
H	3.155794	-2.917808	4.646397
H	3.589194	-3.691126	3.109189
H	-2.933858	-1.857471	2.868651

H	-2.284707	-2.038126	1.233696
H	-1.225411	-2.325298	2.643752

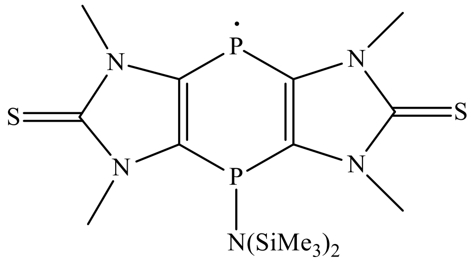

E(M06-2X/6-311+G**) = -2959.562175

N	2.595938	-2.454801	-0.075113
C	1.273230	-2.055952	-0.245943
C	1.314138	-0.720842	-0.602639
N	2.649723	-0.366106	-0.672934
C	3.454916	-1.419515	-0.317672
P	-0.069932	-3.201677	-0.029777
C	-1.382618	-2.045029	-0.321103
C	-1.376460	-0.704093	-0.650404
N	-2.689681	-0.281276	-0.653706
C	-3.534700	-1.307771	-0.299373
N	-2.717170	-2.387612	-0.119927
C	-3.139502	1.075916	-0.904435
P	-0.017679	0.394572	-1.126859
N	-0.042946	1.676363	0.019926
Si	0.088246	1.393775	1.783693
C	-0.828328	-0.138558	2.347000
C	-3.170395	-3.706769	0.279328
S	-5.192489	-1.224286	-0.118327
C	3.187877	0.940517	-1.004538
S	5.119231	-1.395130	-0.196276
C	3.007010	-3.789082	0.319530
Si	0.021070	3.339243	-0.626932
C	0.220838	3.319489	-2.492765
C	-1.554865	4.299065	-0.281789
C	1.497562	4.237012	0.109938
C	1.889607	1.246898	2.285182
C	-0.695914	2.829183	2.710360
H	2.430005	1.509699	-1.539894
H	3.481341	1.470240	-0.096134
H	4.067292	0.803762	-1.631985
H	4.090122	-3.785250	0.419241
H	2.546119	-4.051609	1.273509

H	2.707391	-4.512294	-0.441422
H	-2.544617	1.505358	-1.712815
H	-3.026423	1.687133	-0.006192
H	-4.188152	1.034264	-1.191234
H	-4.257572	-3.691851	0.302123
H	-2.782238	-3.950086	1.270651
H	-2.823068	-4.449260	-0.441068
H	1.956622	1.144002	3.372515
H	2.371801	0.369970	1.844304
H	2.463117	2.133520	2.003090
H	-0.834820	-0.115520	3.441937
H	-1.869310	-0.162733	2.013003
H	-0.339595	-1.064341	2.040181
H	-0.526639	2.656480	3.777926
H	-0.294351	3.815343	2.474361
H	-1.777671	2.851120	2.554472
H	1.532606	5.258790	-0.279419
H	1.472560	4.302551	1.199250
H	2.431737	3.744576	-0.172875
H	0.262094	4.361181	-2.827520
H	1.129950	2.825158	-2.840302
H	-0.623357	2.844091	-2.997167
H	-1.406421	5.337495	-0.594479
H	-2.390461	3.904452	-0.865097
H	-1.847616	4.309960	0.767784

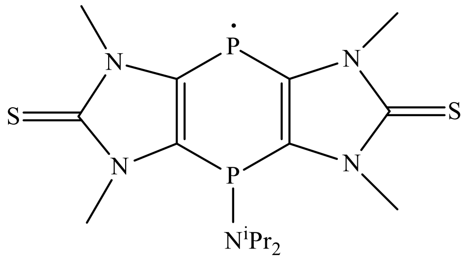

E(M06-2X/6-311+G**) = -2378.050349

N	2.582579	-2.018496	0.108793
C	1.257166	-1.622338	-0.050990
C	1.289892	-0.383412	-0.659839
N	2.621878	-0.072482	-0.858868
C	3.435740	-1.068005	-0.375733
P	-0.089992	-2.639159	0.496467
C	-1.403702	-1.557784	-0.013207
C	-1.404334	-0.303951	-0.594780
N	-2.729215	0.053447	-0.766495

C	-3.567664	-0.923800	-0.289349
N	-2.738522	-1.912026	0.160310
C	-3.232217	1.286612	-1.344147
P	-0.036779	0.783731	-1.101193
N	0.040635	1.959801	0.126121
C	0.194508	3.364846	-0.310218
C	1.372811	4.089127	0.342264
C	-3.188753	-3.163227	0.740368
S	-5.236007	-0.874608	-0.270659
C	3.163643	1.150683	-1.424864
S	5.104282	-1.066978	-0.385578
C	3.000540	-3.271920	0.707325
C	0.079192	1.542797	1.544371
C	-0.866040	2.346289	2.436480
C	1.488647	1.474306	2.149497
C	-1.084364	4.201513	-0.188820
H	3.675728	1.721729	-0.648063
H	3.887576	0.895111	-2.198064
H	2.350927	1.737257	-1.850414
H	4.088124	-3.286640	0.718427
H	2.617859	-3.341967	1.727276
H	2.624330	-4.110395	0.118065
H	-3.571312	1.962016	-0.556489
H	-4.078371	1.048121	-1.986854
H	-2.438052	1.753349	-1.925188
H	-4.276287	-3.149763	0.752110
H	-2.807917	-3.260087	1.758850
H	-2.834332	-4.001262	0.137295
H	-0.304033	0.518151	1.542955
H	-0.947126	5.151695	-0.711609
H	-1.930728	3.684855	-0.641389
H	-1.332910	4.427112	0.848000
H	1.527803	5.045763	-0.161813
H	1.181296	4.303179	1.395464
H	2.291238	3.505534	0.268044
H	-0.917175	1.872374	3.419332
H	-0.516509	3.369852	2.585696
H	-1.872802	2.374393	2.014727
H	1.449684	0.900024	3.078853
H	2.188054	0.973151	1.477292
H	1.885179	2.461012	2.389686
H	0.417048	3.300100	-1.380690

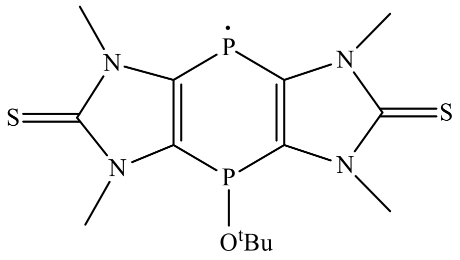

E(M06-2X/6-311+G**) = -2319.331072

N	0.028395	0.002997	0.036364
C	0.014388	0.082433	1.425601
C	1.337654	0.080012	1.825425
N	2.105619	0.014082	0.681995
C	1.309789	-0.058364	-0.437242
P	-1.498137	0.081447	2.347804
C	-0.769541	0.082444	3.969755
C	0.544341	0.072545	4.399594
N	0.514867	0.108842	5.781582
C	-0.777038	0.116399	6.243085
N	-1.555408	0.104703	5.117072
C	1.658091	0.102572	6.680314
P	2.093042	0.218241	3.468849
O	2.907626	-1.193680	3.679872
C	2.479307	-2.591862	3.676992
C	1.947840	-2.936963	5.064153
C	-3.006285	0.122527	5.121384
S	-1.252415	0.127552	7.840957
C	3.555783	-0.072046	0.614253
S	1.832489	-0.207055	-2.013166
C	-1.159724	-0.029049	-0.795427
C	1.435052	-2.881472	2.605155
C	3.767815	-3.351461	3.389571
H	3.978785	0.264495	1.559159
H	3.860373	-1.102401	0.420379
H	3.903552	0.556169	-0.203915
H	-0.836810	-0.076351	-1.833009
H	-1.760000	-0.908543	-0.554054
H	-1.751457	0.873261	-0.630998
H	2.568356	-0.028206	6.097223
H	1.549785	-0.718740	7.389309
H	1.693659	1.039878	7.236374
H	-3.333140	0.118925	6.158875
H	-3.390610	-0.760709	4.608007
H	-3.369382	1.022546	4.621516

H	1.238678	-3.956193	2.596981
H	0.491974	-2.370480	2.808616
H	1.791334	-2.595869	1.612251
H	1.758373	-4.010884	5.129095
H	2.684753	-2.671225	5.824859
H	1.010573	-2.417892	5.275518
H	3.584474	-4.427939	3.408773
H	4.154074	-3.078634	2.405659
H	4.522410	-3.108241	4.139235

compound 4a'

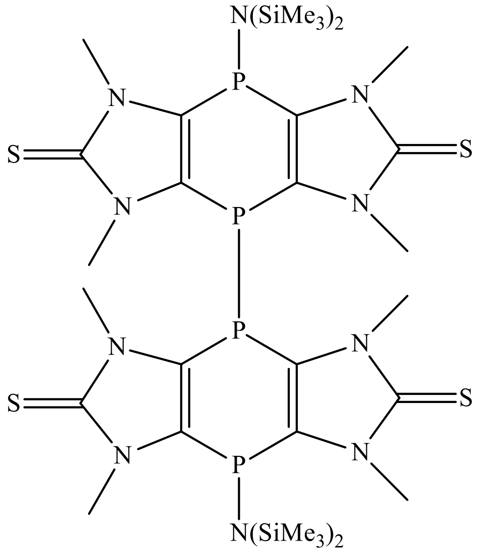

E(M06-2X/6-311+G**) = -5919.201046

C	-2.220974	2.195714	0.261562
C	-3.018540	1.163570	0.651293
N	-3.951488	1.695407	1.532393
C	-3.752083	3.039607	1.707392
N	-2.681581	3.339560	0.904591
P	-2.871957	-0.636063	0.416321
N	-4.191788	-1.100209	-0.588795
Si	-4.491401	-0.400659	-2.206274
C	-4.431879	1.474782	-2.193378
C	-4.965530	0.926919	2.234854
S	-4.615945	4.080696	2.692884
C	-2.123828	4.671522	0.742648
P	-0.794185	2.262516	-0.846325
C	-0.788854	0.516247	-1.306714
C	-1.481016	-0.530538	-0.764291
N	-0.962684	-1.687207	-1.319618
C	0.005829	-1.392046	-2.241720
N	0.126218	-0.028630	-2.208611

C	-1.407164	-3.035769	-1.009152
S	0.843360	-2.460447	-3.223066
C	0.975833	0.694110	-3.145827
P	0.794605	2.263498	0.846264
C	2.221338	2.195943	-0.261441
N	2.681362	3.339170	-0.906001
C	3.750884	3.038446	-1.709864
N	3.950442	1.694436	-1.533799
C	3.018401	1.163430	-0.651236
C	2.123647	4.671254	-0.744847
S	4.613826	4.078739	-2.696951
C	4.963399	0.925090	-2.236898
P	2.871627	-0.635987	-0.415321
N	4.192042	-1.099765	0.589315
Si	4.492798	-0.399374	2.206251
C	4.433614	1.476081	2.192867
C	1.481305	-0.529613	0.766056
N	0.962206	-1.685965	1.321287
C	-0.006162	-1.390273	2.243248
N	-0.125032	-0.026643	2.210957
C	0.789940	0.517649	1.308594
C	1.405315	-3.034760	1.009930
C	-0.972570	0.696410	3.149715
S	-0.845510	-2.458239	3.223527
Si	5.169359	-2.477270	-0.002656
C	6.980726	-1.992202	-0.186639
C	4.551566	-3.014407	-1.685626
C	5.024129	-3.929464	1.188089
Si	-5.169250	-2.477587	0.003096
C	-6.980833	-1.992781	0.185735
C	-4.552503	-3.014123	1.686744
C	-5.022800	-3.930115	-1.187107
C	-3.270364	-1.038559	-3.484982
C	-6.220303	-0.874033	-2.771083
C	3.272658	-1.036595	3.486034
C	6.222061	-0.872786	2.769964
H	-4.515361	0.364456	3.057039
H	-2.109308	4.938030	-0.315575
H	-1.555950	-3.122626	0.071168
H	0.540922	0.649762	-4.146147
H	4.511670	0.360597	-3.056844
H	2.108671	4.938086	0.313277
H	1.553327	-3.121263	-0.070555

H	-0.538051	0.647927	4.150038
H	0.631603	-3.724305	1.342804
H	2.336225	-3.257939	1.534229
H	-1.953151	0.221639	3.172765
H	-1.067462	1.733837	2.830459
H	5.442249	0.239003	-1.538634
H	5.693712	1.624460	-2.638389
H	1.110510	4.710045	-1.152274
H	2.761409	5.362032	-1.292542
H	4.898949	-3.616407	2.227767
H	5.922525	-4.551252	1.136895
H	4.173650	-4.563484	0.924677
H	7.107389	-0.929161	-0.406116
H	7.412570	-2.554568	-1.019754
H	7.568906	-2.219355	0.703473
H	3.497096	-3.298610	-1.701972
H	5.144423	-3.882554	-1.992687
H	4.682293	-2.238788	-2.445018
H	6.409017	-1.947527	2.810620
H	6.343529	-0.484360	3.786013
H	6.992585	-0.412052	2.148554
H	4.940244	1.905477	1.324591
H	4.948535	1.831372	3.091177
H	3.413931	1.863283	2.222743
H	2.297002	-0.550695	3.414547
H	3.668900	-0.821889	4.483396
H	3.115278	-2.116765	3.426211
H	-0.633815	-3.725916	-1.341592
H	-2.337829	-3.257927	-1.534338
H	1.954909	0.216318	-3.170214
H	1.074262	1.730455	-2.823993
H	-5.442341	0.239095	1.536907
H	-5.697207	1.626663	2.633202
H	-1.110519	4.710316	1.149609
H	-2.761336	5.362505	1.290385
H	-4.896474	-3.617326	-2.226724
H	-5.921265	-4.551878	-1.136736
H	-4.172606	-4.564082	-0.922631
H	-7.107877	-0.929736	0.404963
H	-7.413062	-2.555118	1.018678
H	-7.568423	-2.220254	-0.704683
H	-3.498003	-3.298232	1.703812
H	-5.145437	-3.882269	1.993603

H	-4.683798	-2.238336	2.445848
H	-6.407392	-1.948757	-2.811501
H	-6.340962	-0.485929	-3.787351
H	-6.991211	-0.412964	-2.150388
H	-4.939118	1.904468	-1.325595
H	-4.945902	1.830036	-3.092215
H	-3.412089	1.861769	-2.222467
H	-2.294729	-0.552693	-3.412987
H	-3.665888	-0.824244	-4.482717
H	-3.113174	-2.118709	-3.424581

compound 4b'

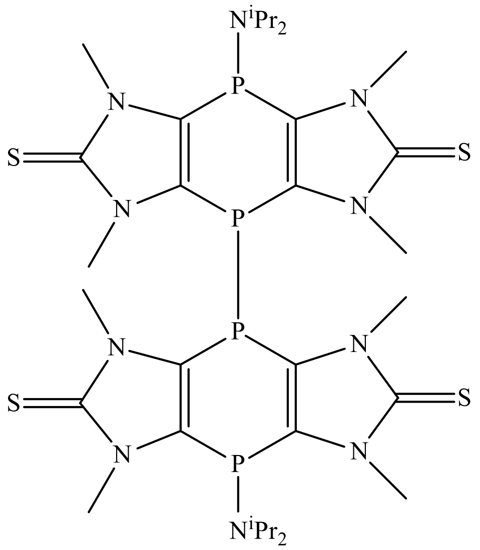

E(M06-2X/6-311+G**) = -4756.180284

C	2.226873	1.923924	-0.067242
C	3.080704	0.895817	0.201086
N	4.103534	1.425699	0.980021
C	3.912469	2.764206	1.193062
N	2.749067	3.063293	0.539678
P	2.930856	-0.904511	-0.055079
N	4.107224	-1.340888	-1.195525
C	4.260716	-0.826568	-2.578532
C	4.195936	0.693310	-2.690422
C	5.253055	0.700580	1.490204
S	4.876242	3.816243	2.066284
C	2.163349	4.392887	0.550491
P	0.655707	1.981979	-0.958676
C	0.586127	0.236631	-1.415007
C	1.362937	-0.807981	-0.997451
N	0.757948	-1.965096	-1.460696

C	-0.355818	-1.670512	-2.201988
N	-0.465919	-0.307553	-2.154062
C	1.237020	-3.312055	-1.199366
S	-1.361923	-2.737207	-3.015993
C	-1.453868	0.415469	-2.944368
P	-0.655792	1.982073	0.958476
C	-2.227004	1.923923	0.067137
C	-3.080823	0.895787	-0.201109
N	-4.103666	1.425629	-0.980066
C	-3.912676	2.764148	-1.193092
N	-2.749265	3.063281	-0.539738
P	-2.930851	-0.904538	0.055040
N	-4.107045	-1.341024	1.195627
C	-4.260424	-0.826708	2.578661
C	-3.303240	-1.493855	3.570498
C	-5.253141	0.700465	-1.490289
S	-4.876537	3.816093	-2.066335
C	-2.163475	4.392841	-0.550545
C	-1.362876	-0.807901	0.997299
N	-0.757871	-1.964983	1.460613
C	0.355827	-1.670325	2.201984
N	0.465854	-0.307364	2.154051
C	-0.586142	0.236757	1.414884
C	-1.236880	-3.311978	1.199328
C	1.453761	0.415723	2.944343
S	1.361874	-2.736948	3.016146
C	-5.011402	-2.488570	0.917876
C	-4.605794	-3.354936	-0.271256
C	-6.456226	-2.017132	0.720360
C	5.011627	-2.488374	-0.917691
C	4.606024	-3.354782	0.271419
C	6.456398	-2.016825	-0.720072
C	3.303878	-1.494036	-3.570492
C	-4.196108	0.693199	2.690493
H	-1.636901	4.570437	-1.490703
H	-1.473561	4.485062	0.288375
H	-2.964952	5.123968	-0.454484
H	1.189325	0.360932	4.001814
H	2.428059	-0.051409	2.804360
H	1.489295	1.455377	2.620131
H	-0.493831	-4.006765	1.586024
H	-2.189433	-3.476268	1.707147
H	-1.364608	-3.444408	0.121319

H	-5.590445	1.200661	-2.396438
H	-4.958412	-0.325840	-1.714194
H	-6.061379	0.701980	-0.756404
H	-2.428122	-0.051775	-2.804455
H	-1.489529	1.455107	-2.620118
H	-1.189383	0.360740	-4.001832
H	0.494115	-4.006896	-1.586242
H	2.189697	-3.476204	-1.706998
H	1.364575	-3.444533	-0.121341
H	6.061195	0.701953	0.756211
H	5.590484	1.200913	2.396231
H	4.958337	-0.325688	1.714309
H	1.637890	4.570945	1.491176
H	2.964709	5.123978	0.453135
H	1.472457	4.484708	-0.287662
H	-4.973718	-3.124050	1.813121
H	-7.140679	-2.867922	0.760520
H	-6.767561	-1.297621	1.479302
H	-6.562833	-1.546663	-0.259218
H	-5.300844	-4.195126	-0.332928
H	-4.658122	-2.805656	-1.213745
H	-3.595717	-3.753184	-0.182208
H	4.974085	-3.123859	-1.812939
H	5.301127	-4.194924	0.333125
H	4.658283	-2.805494	1.213908
H	3.595975	-3.753097	0.182345
H	7.140902	-2.867579	-0.760173
H	6.767746	-1.297311	-1.479004
H	6.562908	-1.546338	0.259514
H	5.272855	-1.116998	-2.873073
H	3.587799	-1.227573	-4.591574
H	3.343563	-2.582717	-3.489472
H	2.273245	-1.168750	-3.417825
H	4.537172	0.987940	-3.685479
H	3.177175	1.063155	-2.566462
H	4.835638	1.175024	-1.947434
H	-5.272405	-1.117467	2.873410
H	-4.537479	0.987794	3.685512
H	-3.177448	1.063342	2.566549
H	-4.835911	1.174677	1.947438
H	-3.587109	-1.227437	4.591606
H	-3.342639	-2.582550	3.489516
H	-2.272720	-1.168284	3.417706

compound 4c’

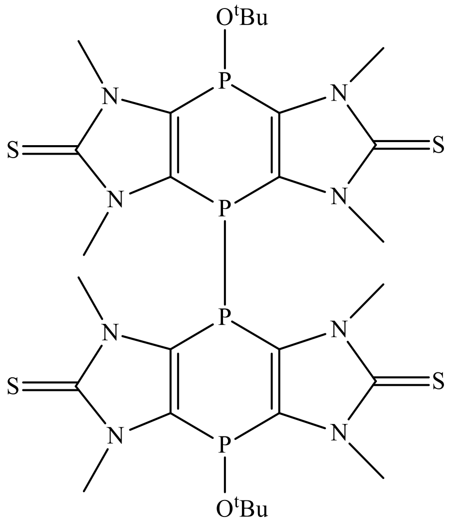

E(M06-2X/6-311+G**) = -4638.731827

N	-3.996019	1.148733	1.211406
C	-3.045732	0.691782	0.301295
C	-2.227248	1.750007	0.028004
N	-2.695155	2.831583	0.761370
C	-3.780878	2.464041	1.512812
P	-3.045879	-1.009365	-0.344258
O	-3.817989	-0.770136	-1.803015
C	-5.057913	-1.450244	-2.125231
C	-5.446772	-0.878603	-3.481553
P	-0.700603	1.861247	-0.939527
C	-0.592735	0.122439	-1.407578
C	-1.354283	-0.938724	-1.008382
N	-0.710674	-2.084158	-1.450839
C	0.429723	-1.762012	-2.131110
N	0.488943	-0.393488	-2.112055
C	-1.174148	-3.444789	-1.221003
S	1.546163	-2.803931	-2.826101
C	1.407283	0.353684	-2.961710
C	-2.128246	4.168755	0.814473
S	-4.623914	3.419089	2.593511
C	-4.994520	0.351866	1.908060
P	0.700603	1.861312	0.939454
C	2.227241	1.750003	-0.028080
C	3.045735	0.691767	-0.301291
N	3.996012	1.148656	-1.211441
C	3.780847	2.463934	-1.512964
N	2.695129	2.831526	-0.761538
P	3.045882	-1.009341	0.344354

O	3.818039	-0.770017	1.803068
C	5.057990	-1.450079	2.125276
C	4.816967	-2.953400	2.218705
C	4.994547	0.351740	-1.907991
S	4.623872	3.418907	-2.593739
C	2.128187	4.168680	-0.814736
C	0.592744	0.122534	1.407622
N	-0.488941	-0.393350	2.112119
C	-0.429752	-1.761874	2.131208
N	0.710653	-2.084063	1.450972
C	1.354287	-0.938654	1.008484
C	-1.407275	0.353867	2.961743
C	1.174078	-3.444714	1.221158
S	-1.546233	-2.803751	2.826195
C	6.119487	-1.124338	1.078745
C	5.446868	-0.878384	3.481570
C	-6.119451	-1.124507	-1.078741
C	-4.816832	-2.953560	-2.218605
H	-5.741510	-3.467255	-2.491153
H	4.731133	-0.701618	-1.818083
H	2.388536	-0.113269	-2.915645
H	1.467257	1.387115	-2.623015
H	1.053327	0.323116	-3.993965
H	-2.942721	4.891842	0.798206
H	-1.558363	4.303801	1.736018
H	-1.483296	4.315894	-0.051950
H	-4.994195	0.648469	2.956962
H	-5.988317	0.533367	1.496950
H	-4.731147	-0.701501	1.818156
H	-0.327701	-4.112733	-1.368237
H	-1.555018	-3.525495	-0.200367
H	-1.957231	-3.703362	-1.935784
H	1.558120	4.303582	-1.736188
H	1.483404	4.315939	0.051792
H	2.942658	4.891776	-0.798735
H	4.994317	0.648322	-2.956899
H	5.988317	0.533213	-1.496803
H	1.957126	-3.703318	1.935964
H	0.327597	-4.112620	1.368374
H	1.554972	-3.525446	0.200535
H	-2.388529	-0.113086	2.915706
H	-1.053314	0.323346	3.993997
H	-1.467247	1.387283	2.623000

H	-4.066742	-3.164548	-2.984194
H	-4.475465	-3.357075	-1.261225
H	-6.367415	-1.345497	-3.838663
H	-5.602758	0.198632	-3.404019
H	-4.652264	-1.063536	-4.206195
H	-7.090826	-1.512778	-1.392659
H	-5.874598	-1.577980	-0.114811
H	-6.201919	-0.041599	-0.955169
H	6.367542	-1.345229	3.838664
H	5.602807	0.198856	3.404000
H	4.652390	-1.063329	4.206242
H	7.090874	-1.512615	1.392620
H	5.874592	-1.577798	0.114819
H	6.201953	-0.041430	0.955183
H	5.741673	-3.467051	2.491243
H	4.066908	-3.164390	2.984323
H	4.475590	-3.356961	1.261347

References:

1. Gaussian 09, Revision E.01, M. J. Frisch, G. W. Trucks, H. B. Schlegel, G. E. Scuseria, M. A. Robb, J. R. Cheeseman, G. Scalmani, V. Barone, B. Mennucci, G. A. Petersson, H. Nakatsuji, M. Caricato, X. Li, H. P. Hratchian, A. F. Izmaylov, J. Bloino, G. Zheng, J. L. Sonnenberg, M. Hada, M. Ehara, K. Toyota, R. Fukuda, J. Hasegawa, M. Ishida, T. Nakajima, Y. Honda, O. Kitao, H. Nakai, T. Vreven, J. A. Montgomery, Jr., J. E. Peralta, F. Ogliaro, M. Bearpark, J. J. Heyd, E. Brothers, K. N. Kudin, V. N. Staroverov, T. Keith, R. Kobayashi, J. Normand, K. Raghavachari, A. Rendell, J. C. Burant, S. S. Iyengar, J. Tomasi, M. Cossi, N. Rega, J. M. Millam, M. Klene, J. E. Knox, J. B. Cross, V. Bakken, C. Adamo, J. Jaramillo, R. Gomperts, R. E. Stratmann, O. Yazyev, A. J. Austin, R. Cammi, C. Pomelli, J. W. Ochterski, R. L. Martin, K. Morokuma, V. G. Zakrzewski, G. A. Voth, P. Salvador, J. J. Dannenberg, S. Dapprich, A. D. Daniels, O. Farkas, J. B. Foresman, J. V. Ortiz, J. Cioslowski, and D. J. Fox, Gaussian, Inc., Wallingford CT, 2013.
